# Direct Synthesis of 2-Hydroxytrifluoroethylacetophenones via Organophotoredox-Mediated Net-Neutral Radical/Polar Crossover

**DOI:** 10.1021/acs.joc.4c01419

**Published:** 2024-08-01

**Authors:** Albert Gallego-Gamo, Pau Sarró, Yingmin Ji, Roser Pleixats, Elies Molins, Carolina Gimbert-Suriñach, Adelina Vallribera, Albert Granados

**Affiliations:** † Department of Chemistry and Centro de Innovación en Química Avanzada (ORFEO−CINQA), Universitat Autònoma de Barcelona, Cerdanyola del Vallès, 08193 Barcelona, Spain; ‡ Institut de Ciència de Materials de Barcelona (ICMAB-CSIC), Campus UAB, 08193 Bellaterra, Spain

## Abstract

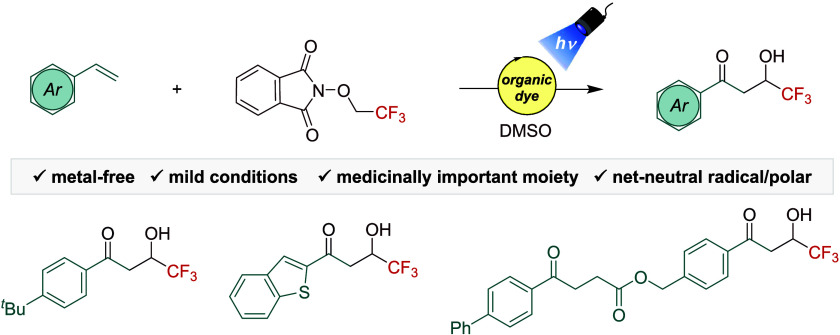

Alkene difunctionalization is a very attractive tool in synthetic organic chemistry. Herein, we disclose an operationally and practically simple method to access 2-hydroxytrifluoroethylacetophenones from styrene derivatives via photoredox catalysis. This light-mediated transformation promotes the generation of the 1-hydroxy-2,2,2-trifluoroethyl carbon-centered radical as key synthon, which undergoes Giese addition with styrenes followed by a Kornblum oxidation process. The presented method is not only mild and cost-effective, but also utilizes an organic photocatalyst and DMSO as oxidant. Experimental investigations support the operative mechanism via net-neutral radical/polar crossover.

## Introduction

Alkene difunctionalization is generally used to introduce two functional groups in an olefin simultaneously and build complex molecular organic skeletons in a single step. Specifically, transition-metal catalyzed^1^ and photoredox^2^ approaches have shown their potential for successful 1,2-difunctionalization reactions. Typically, this process involves the installation of two functional groups across an olefin to generate C–C and C–Z (Z = C, O, N, S or halogen) bonds, allowing prompt formation of elaborated organic skeletons.

Fluorine is one of the most important heteroatoms in pharmaceuticals both as a single atom or within a functional group, constituting a fundamental element in medicinal chemists’ toolbox.^3^ Thus, routine incorporation of such halogenated moieties has been long studied in new drug design programs. The presence of fluorine or fluorinated groups in a bioactive molecule can provide better metabolic stability, lipophilicity, and binding selectivity.^4^ Undoubtedly, the trifluoromethyl group is widely prevalent in many agrochemicals and pharmaceuticals,^5^ and extensive attention has been devoted toward the design and development of efficient synthetic methods to provide access to CF_3_-containing organic architectures. In recent years, developments in radical,^6^ nucleophilic^7^ and electrophilic^8^ approaches have advanced in the field using CF_3_I, Langlois, Ruppert–Prakash, Togni, Umemoto reagents, and others.

**Scheme 1:**
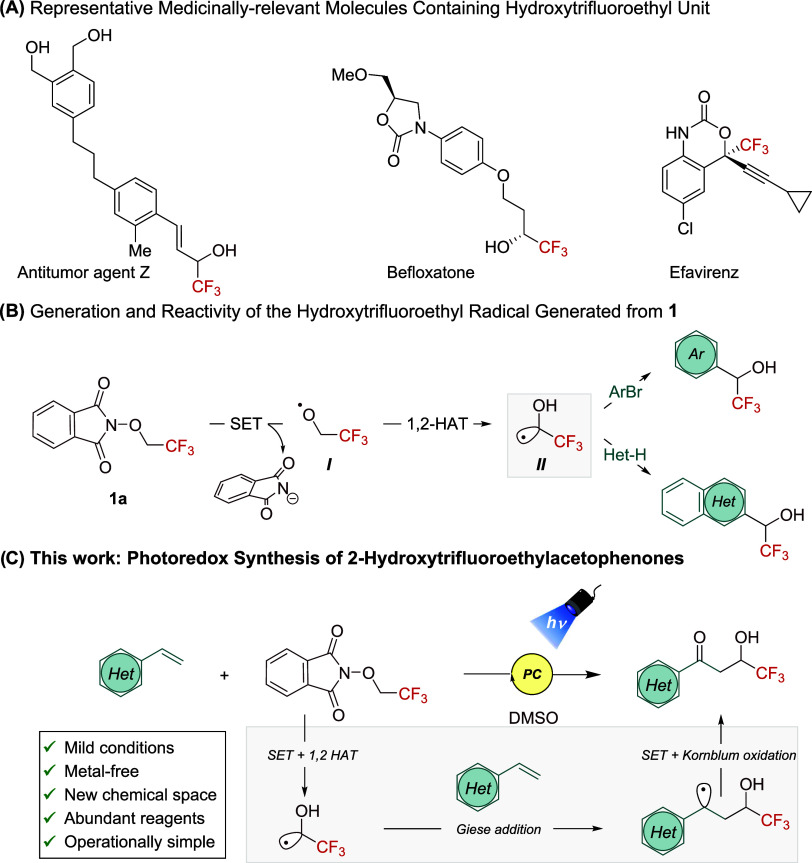
(A) Representative Bioactive Hydroxytrifluoroethyl-Containing Molecules. (B) Generation and Application of the 1-Hydroxy-2,2,2-trifluoroethyl Radical from **1a**. (C) This work PC = Photocatalyst.

Among different fluorine-containing molecules, secondary trifluoromethyl alcohols are a particular and interesting subset of trifluoromethylated molecules that have relevant applications in medicinal and biological chemistry (Scheme 1A).^9^ For example, Befloxatone, antitumor agent Z and Efavirenz are representative examples of bioactive molecules containing such functionality (Scheme 1A).^10^ Typically, accessing hydroxytrifluoroethylated compounds relies on the use of the Ruppert-Prakash^11a^ (TMSCF_3_) reagent, trifluoroacetaldehyde ethyl hemiacetal,^11b^ among others.^7b^ Additionally, these molecules can be accessed by direct reduction of trifluoroacetates.^12^ However, these routes require cryogenic temperatures and present low functional group tolerance. Given the importance of this functional group, the development of synthetic methods for the direct installation of hydroxytrifluoroethyl group in organic molecules have been recently explored, where single electron transfer (SET) approaches have opened a new avenue for the selective introduction of such moieties.^13^

Recently, *N*-trifluoroethoxyphthalimide **1a** (Scheme 1B) as redox-active ether has proved to be an efficient and versatile reagent for organic synthesis. First, Aggarwal presented that upon reductive conditions and in the presence of suitable hydrogen donors, reagent **1a** provides the oxygen-centered radical ***I*** (Scheme 1B), which performs a hydrogen atom transfer (HAT) process on unactivated Csp^3^–H bonds.^14^ Later on, different research groups have shown that radical **I** generated from **1a** can undergo intramolecular 1,2-HAT to produce the synthetically useful carbon-centered radical **II**.^13^ For instance, in 2022 a nickel-catalyzed reductive cross-electrophile coupling between redox-active ether **1a** and haloarenes was reported, where the 1,2-HAT event from **I** to **II** is the key step for the synthesis of α-aryl-α-trifluoromethyl alcohols.^15^ Furthermore, **1a** can efficiently produce the carbon-centered radical **II** under photochemical conditions.^16^ Overall, these methods employing reagent **1a** have shown their efficiency toward the generation of the carbon-centered radical **II** via 1,2-HAT, although to date their application is limited to the synthesis of α-(hetero)aryl-α-trifluoromethyl alcohols. Thus, the exploration of additional organic frames for accommodating radical **II** beyond (hetero)arenes is highly desirable since it would lead to the expedition of new chemical space in organofluorine settings.

Inspired by visible-light promoted preparation of ketones using redox-active species and alkenes,^17^ we decided to explore the photochemical generation of radical **II** and subsequent addition to styrenes. This reaction features both radical and ionic modes of reactivity. The success of this design involves addressing several challenges, from the N–O bond cleavage of **1a** via SET to trigger the single electron reduction and oxidation processes along with the 1,2-HAT event. All these processes must be thrived in a controlled and well-orchestrated manner (Scheme 1C), via photoinduced net-neutral radical polar crossover (RPC).^2b^ Interestingly, this reaction will provide access to a prominent variety of 2-hydroxytrifluoroethylacetophenones in a single step from readily available reagents and mild reaction conditions. Of note, access to β-CF_3_-enones from 2-hydroxytrifluoroethylacetophenones is feasible upon dehydration conditions.^18^

## Results and Discussion

**Scheme 2:**
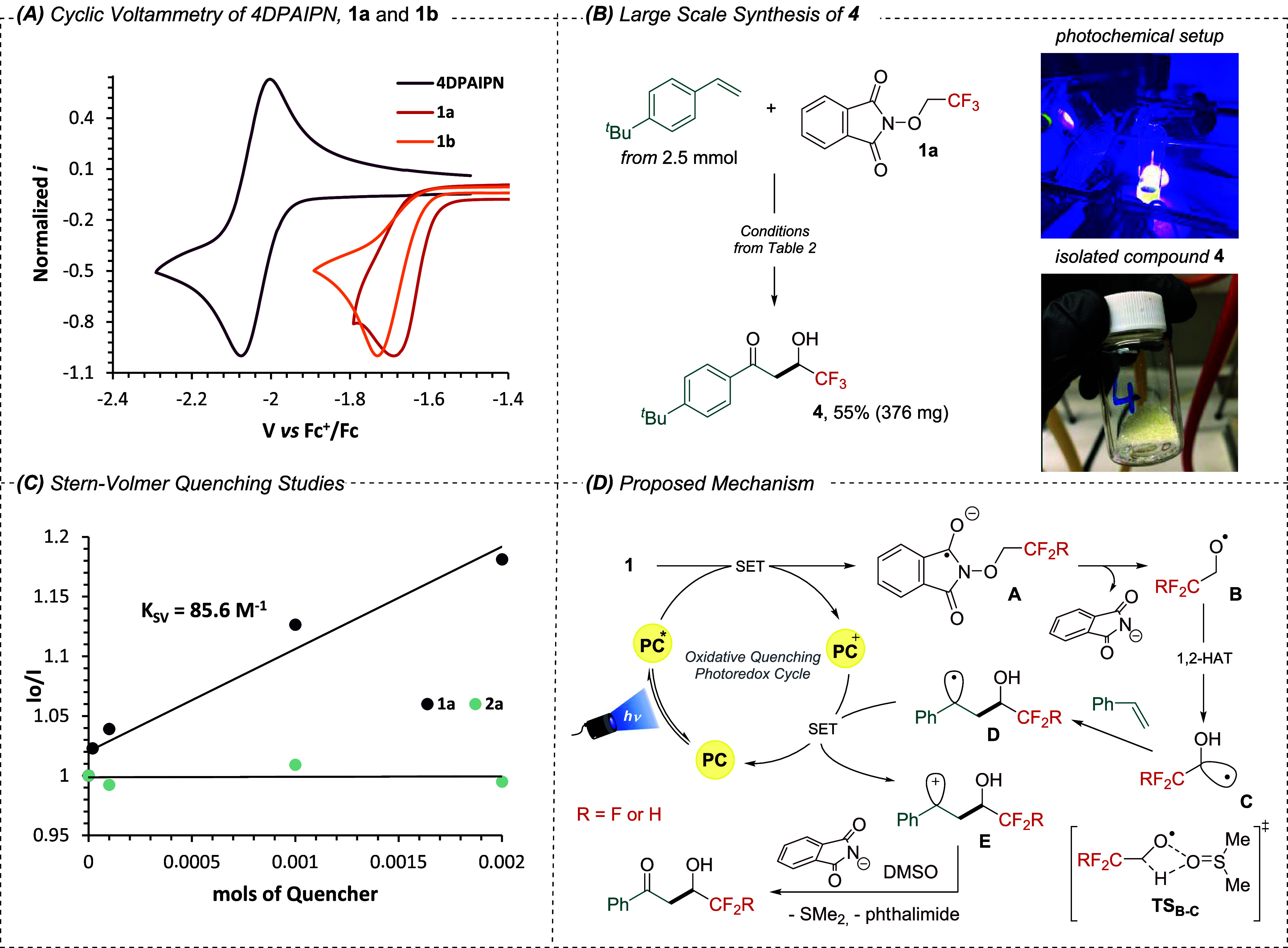
(A) Electrochemical Studies of Compound **1a**, **1b**, and 4DPAIPN. (B) Large Scale Synthesis of 4 from 2.5 mmol of *p*-*tert*-Butylstyrene, Yield Value after Purification Process. (See Supporting Information for Further Information). (C) Stern–Volmer Luminescence Quenching Plot. (D) Proposed Mechanism for the Photoinduced Olefin Difunctionalization via RPC PC = photocatalyst. 1,2-HAT = 1,2-hydrogen atom transfer.

First, the selection of the photocatalyst (PC) to trigger this reaction governed by oxidative quenching photoredox conditions is critical. Thus, we studied the electrochemical properties of fluorinated reagent **1a** (*E*_c_ = −1.63 V vs Fc^+^/Fc, see Scheme 2A)^19^ and the model styrene substrate **2a** (*E*_a_ = 0.81 V vs Fc^+^/Fc). With these results in hand, we selected the highly reducing Ir(ppy)_3_ with *E*_1/2_ = −2.45 V vs Fc^+^/Fc Scheme 2A,^19^ (see Figure S3 in the Supporting Information) as a potential candidate capable of reducing reagent **1a**. To our delight, we observed the product formation (**3**) in 64% yield (Table 1, entry 1) under 427 nm Kessil light irradiation for 24 h and using an excess of radical precursor **1a** (2.0 equiv). Remarkably, the use of DMSO is displaying a good ability to perform the 1,2-HAT process. To date, synthetic methods where a 1,2-HAT event occurs from **1** have been effective only in dimethylacetamide (DMA) or MeOH. The formation of trifluoroacetaldehyde-hydrate as main side product was detected by ^19^F NMR. In our case, (Ir((dF)(CF_3_)ppy)_2_(dtbpy))PF_6_ did not provide better results (Table 1, entry 2), which proved to be suitable in previous photoinduced methods employing reagent **1a**.^13^ Attempts to use organophotocatalysts (Table 1, entries 3–4) only furnished the desired acetophenone derivative **3** when using 1,3-dicyano-2,4,5,6-tetrakis(diphenylamino)-benzene (4DPAIPN) (*E*_1/2_ = −1.99 V vs Fc^+^/Fc). Although organic 1,2,3,5-tetrakis(carbazol-9-yl)-4,6-dicyanobenzene (4CzIPN) may thermodynamically reduce **1a** it can also oxidize styrene **2a**, thus allowing a competition between oxidative and reductive quenching pathways. No differences were observed between Ir(ppy)_3_ and 4DPAIPN, thus we decided to continue with the more accessible organic PC. Lowering or increasing loadings of 4DPAIPN resulted in less efficacy (Table 1, entries 5–6). Interestingly, more energetic wavelength (Table 1, entry 7) resulted in the formation of complex reaction mixtures and product decomposition. In contrast, when using blue Kessil irradiation reagent **1a** was not totally consumed (Table 1, entry 8). As expected, control studies confirmed the necessity of 4DPAIPN and light irradiation for efficient reactivity (Table 1, entries 9–10). Remarkably, we detected that this reaction is completed after only 90 min (Table 1, entry 11) of illumination and DMSO is crucial for success (Table 1, entry 12).

**Table 1:**
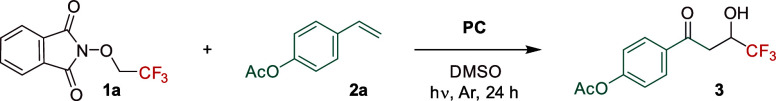

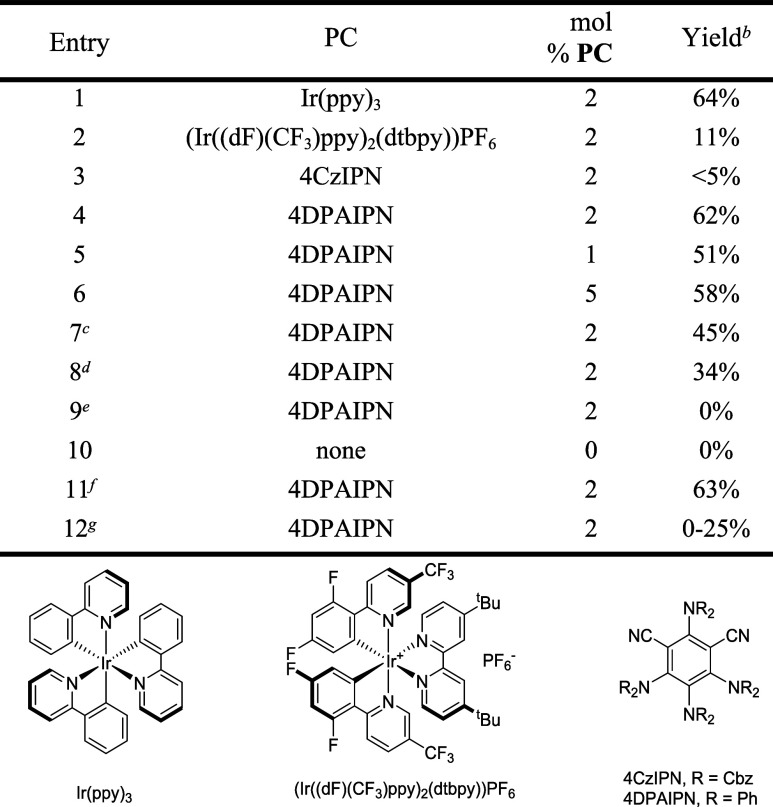
Exploration of the Reaction Conditions^a^

a Reaction conditions: **1a** (0.2 mmol, 2 equiv), **2a** (0.1 mmol, 1 equiv), photocatalysts (**PC**) (indicated amounts) in 0.5 mL of DMSO (*c* = 0.2 M) under violet Kessil lamp irradiation (λ_max_ = 427 nm) at rt for 24 h.

b Yields were determined by ^1^H NMR analysis using 1,3,5-trimethoxybenzene as internal standard.

c Purple Kessil irradiation (λ_max_ = 390 nm).

d Blue Kessil irradiation (λ_max_ = 456 nm).

e No light.

f Irradiation for 90 min ppy = phenylpyridine. dtbpy = di-*tert*-butylpyridine. Cbz = carbazole.

g DMSO in combination with DMA, MeCN or DMF (1:1).

With a suitable set of conditions established, we turned our attention to evaluate the substrate scope of the oxidative hydroxytrifluoroethylation process with a range of commercially available styrenes (Table 2). In general, unsubstituted styrenes, as well as electron-rich and electron-poor groups tethered to the phenyl ring presented comparable reactivity. The reaction is tolerant toward esters, ethers, ketones, bromides, and several heterocycles. Electron-rich 4-substituted styrenes worked well under the optimized reaction conditions, providing the desired acetophenones in yields that ranged from 54 to 66% (**3**–**7**). Unsubstituted styrene and 2-vinylnaphthalene also demonstrated to be suitable substrates for this difunctionalization process (**8**–**9**). The *ortho-* and *para*-substituted acetophenone **10** was isolated in a moderate 45% yield, while bulkier groups in *ortho* position (such as bromide substituent) were not well tolerated. Moreover, 3-fluorostyrene yielded the 2-hydroxytrifluoroethylacetophenone **11** in moderate yield. Of note, the structure of the synthesized products was demonstrated by single-crystal X-ray diffraction study of compound **11**, where linked molecules pairs were found for an expected double hydrogen bond (Table S6 in the Supporting Information). Then, *para*-halogenated styrenes also showed likewise efficacy the unsubstituted or more electronically rich styrenes (**12**–**14**). Interestingly, complete retention of the bromide moiety (**14**) opens new opportunities for further functionalization. Interestingly, difunctionalization of indene and dialin fused rings provided the hydroxytrifluoroethylated carbonyls (**15** and **16**) in moderate yield and diastereomeric ratio. On the other hand, functionalization of disubstituted analogues such as (2-methylprop-1-en-1-yl)benzene did not proceed well. Heterocyclic compounds including benzothiophene (**17** and **18**) and benzofurane (**19**) skeletons were readily incorporated under the optimal reaction conditions. Next, we evaluated the amenability of this process to more architecturally complex alkenes derived from nonsteroidal anti-inflammatory styrene derivatives like ibuprofen (**20**), fenbufen (**21**) or flurbiprofen (**22**). Our investigation revealed that these substrates can accommodate the hydroxytrifluoroethyl group in an efficient manner. Thus, we provide quick access to analogs of such structures with the pharmacologically relevant trifluoroethanol group.

**Table 2:**
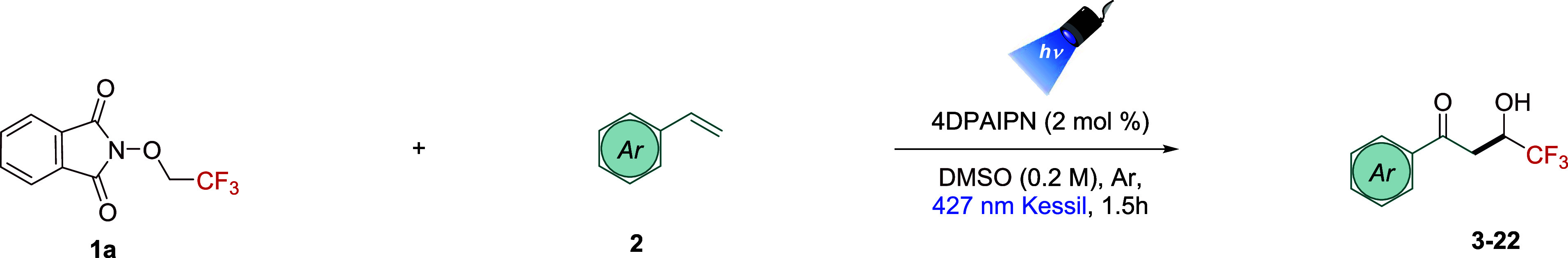

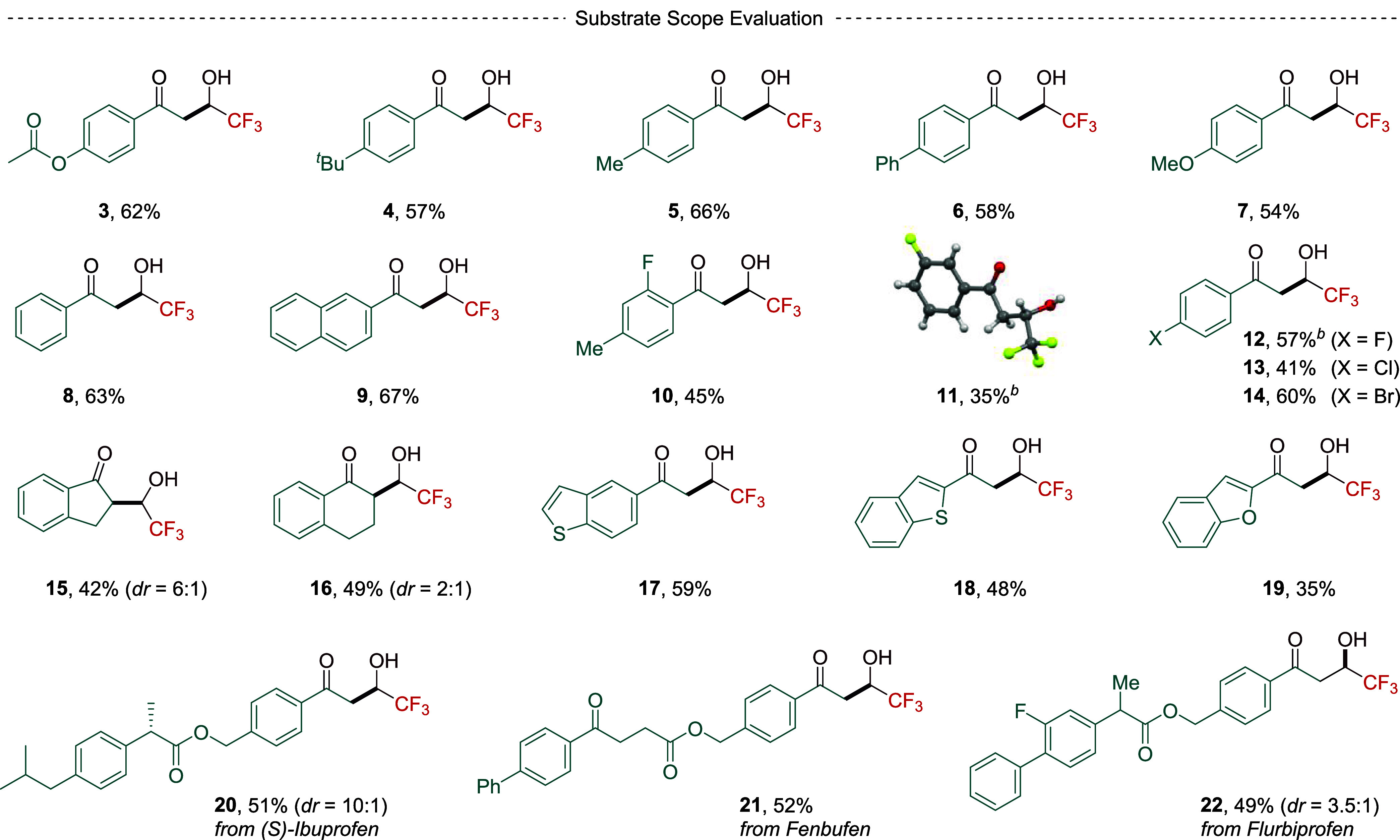
Evaluation of Substrate Scope^a^

a General reaction conditions: **1a** (1.0 mmol, 2 equiv), **2** (0.5 mmol, 1 equiv), DPAIPN (2 mol %) in DMSO (2.5 mL, 0.2 M), under Kessil lamp irradiation (λ_max_ = 427 nm) at rt for 90 min.

b Irradiation for 3 h. Yield values after purification process.

Within the field of drug design, the difluoromethyl (−CF_2_H) group is recently earning considerable attention because it has been proved to be a more metabolically stable bioisostere of thiol and alcohol groups and a good lipophilic hydrogen bond donor.^20^ Despite the significance of this chemical space, the availability of bifunctional CF_2_H sources as effective reagents is limited.^21^ The bench-stable *N*-difluoroethoxyphthalimide **1b** was prepared^22^ and tested in the difunctionalization process (Table 3). First, we detected that this reaction needed longer reaction time to be completed. In the crude ^1^H NMR we were delighted to recognize good reactivity, however, degradation of the desired compound was observed upon purification by flash column chromatography leading to poor yields (see characterization of compound **26** in the Supporting Information). Given this experimental obstacle, we planned to reduce the carbonyl group in a two-step process to yield the corresponding diol. Following this strategy, reagent **1b** provided the desired diols **23**–**25** from low to moderate yields (Table 3). Particularly, reagent **1b** not only can be reduced by 4DPAIPN (*E*_c_= −1.67 V vs Fc^+^/Fc, see Scheme 2A), but also the generated alkoxy radical undergoes 1,2-HAT event efficiently producing the desired difluoromethylene compounds.

**Table 3:**
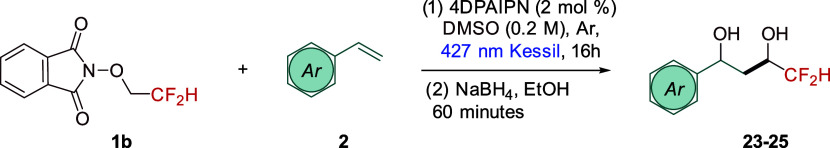

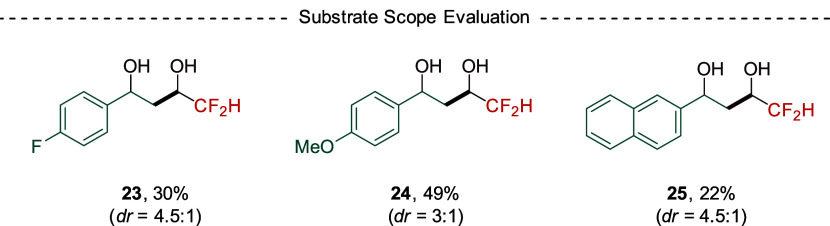
Substrate Scope Using Difluoroethylated Reagent **1b**^a^

a General reaction conditions: (1) **1b** (1.0 mmol, 2 equiv), **2** (0.5 mmol, 1 equiv), 4DPAIPN (2 mol %) in DMSO (2.5 mL, 0.2 M), under violet Kessil lamp irradiation (λ_max_ = 427 nm) at rt for 16 h. (2) NaBH_4_ (1.5 mmol) in EtOH (2.5 mL, 0.2 M) for 60 min under ambient atmosphere. Yield values after purification process.

Next, we probed the scalability of this protocol in a 2.5 mmol scale using 4-*tert*-butylstyrene as a model alkene and reagent **1a**. The reaction scale was increased 5-fold in batch with comparable yield (Scheme 2B) obtaining 376 mg of **4**.

We then endeavored to gain a deeper understanding of the mechanism of this difunctionalization process. We speculated that reagent **1a** undergoes an irreversible and reductive SET process triggered by the PC to afford the radical ion intermediate, where the oxygen centered radical is then generated after mesolytic N–O bond fragmentation. This was experimentally supported by Stern–Volmer luminescence quenching studies. Mixtures of 4-acetoxystyrene model and **1a** with 4DPAIPN revealed that the excited state of the photocatalyst is quenched most effectively by the fluorinated redox active species **1a** rather than by olefinic substrate, with an observed constant *K*_SV_ of 85.6 M^–1^ (Scheme 2C and Supporting Information). Next, a radical trapping experiment with TEMPO revealed the involvement of radical species during the mechanism (see Supporting Information) since no product was formed.

Based on these mechanistic findings, the electrochemical data and related literature,^13^ a plausible mechanism is presented in Scheme 2D. Upon photoexcitation of 4DPAIPN under light irradiation (λ_max_ = 427 nm), a highly reducing excited state *4DPAIPN is generated (*E*^(PC+/*)^ = −1.99 V vs Fc^+^/Fc, see Table S1 in the Supporting Information). Single electron transfer process to redox active species **1** (*E*_a_= −1.63 V vs Fc^+^/Fc for **1a** and *E*_a_ = −1.67 V vs Fc^+^/Fc for **1b**) forms the reduced radical anion species **A**, which delivers the phthalimide anion and the oxygen-centered radical **B** via β-scission. Then, we propose that C(sp^3^)-hybridized radical **C** is formed from **B** by intramolecular 1,2-hydrogen atom transfer (1,2-HAT) event promoted by DMSO (TS_B–C_ in Scheme 2D).^13^ Subsequently, **C** undergoes Giese addition to vinyl arene yielding a relatively stabilized secondary and benzylic radical **D** (*E*_a_ = 0.37 V vs SCE).^23^ This open-shell intermediate is oxidized to carbocation **E** by PC^+^ (*E*^PC+/0^ = 0.63 V vs Fc^+^/Fc), restoring the photocatalytic cycle. Subsequent transformation of intermediate **E** promoted by DMSO and phthalimide anion^17^ provides access to 2-hydroxydifluoro- and 2-hydroxytrifluoroethylacetophenones.

## Conclusions

The presented synthetic method addresses a pressing demand in the synthesis of fluorinated small molecules. We are presenting the first incorporation of the hydroxytrifluoroethyl group into alkenes from the photochemical reduction of *N*-trifluoroethoxyphthalimide. This difunctionalization process exploits the in situ generation of the key carbon-centered α-hydroxy-α-trifluoroethyl radical facilitated by DMSO. Access to 2-hydroxytrifluoroethylacetophenones is expedited using the organic photoredox 4DPAIPN species and mild oxidation conditions. The synthesis of difluoromethylene analogues is also feasible under the reported conditions. This synthetic method is found to be suitable in the difunctionalization of simple and more complex styrenes and related heteroaromatics. Lastly, mechanistic experiments support the operation via net-neutral radical/polar crossover photoredox cycle.

## Experimental Section

### General Information

All chemical transformations requiring inert atmosphere were done using Schlenk line techniques. For violet light irradiation, a Kessil PR160-violet LED lamp (30 W High Luminous DEX 2100 LED, λ_max_ = 427 nm) was placed 4 cm away from the reaction vials. Photoinduced reactions were performed using 4 or 8 mL Chemglass vials (15–425 Green Open Top Cap, TFE Septa). Reactions were monitored by TLC or NMR. TLC analysis was performed using hexanes/EtOAc mixtures as the eluent unless specified and visualized using ultraviolet (UV) light and/or Vanillin solution. The cyclic voltammetry (CV) experiments were performed with a BioLogic SP-50 Single Channel Potentiostat in a one-compartment three-electrode setup using a glassy carbon disk as the working electrode (ø = 3 mm), platinum wire as the auxiliary electrode, and SCE or AgNO_3_/Ag (0.01 M AgNO_3_, 0.1 M [NBu_4_N]PF_6_ (TBAPF_6_), MeCN) as reference electrodes. CV were performed at room temperature using the appropriate solvent, degassing with argon for 60 s and using TBAPF_6_ as supporting electrolyte (0.1 M). All the experiments were referred to ferrocene as an internal standard. Polishing of the working electrode has been done using an alumina polishing pad with a solution of 0.05 μm alumina in water (purchased from BAS INC.). NMR experiments (^1^H, ^13^C, ^19^F) were performed in the *Servei de Ressonància Magnètica Nuclear*, UAB, using NEO 300, 400, 500, or 600 spectrometers. Chemical shifts are referenced to residual, nondeuterated CHCl_3_ (δ 7.26 in ^1^H NMR and 77.16 in ^13^C NMR). The HRMS (ESI+) and elemental analyses were done by the *Servei d’Anàlisi Química* of UAB and *Parque Científico Tecnológico* of UBU. HRMS determined by a Bruker microTOF-QII mass spectrometer (fly time analyzer) through positive electrospray ionization. IR spectra were recorded on an FT-IR PerkinElmer using either neat oil or solid products. Fluorescence measurements were obtained using septa-capped UV-Quartz cuvettes (10 mm path length) from Hellma Analytics and were recorded in a PerkinElmer LS 55 Fluorescence Spectrometer attached to a PTP 1 Peltier Temperature Programmer maintaining the temperature at 25 °C. Melting points (°C) are uncorrected. Deuterated NMR solvents were purchased from Eurisotop. Dry solvents were obtained from Aldrich or Fisher and used as received. Bulk DCM, EtOAc and hexane were purchased from VWR. Chemicals were purchased from Fluorochem and Merck and used as received unless specified.

#### General Procedure for the Photoinduced Synthesis of 2-Hydroxytrifluoroethylacetophenones (**3**–**22**)

To a flame-dried 4 mL vial equipped with a magnetic stir bar, redox active ether **1a** (245.0 mg, 1.0 mmol, 2.0 equiv), the corresponding styrene (0.5 mmol, 1.0 equiv) and 4DPAIPN (7.9 mg, 0.01 mmol, 0.02 equiv) were dissolved in 2.5 mL of dry DMSO. Afterward, the solution was degassed with Argon for 20 s. The reaction mixture was irradiated for 90 min with a 427 nm Kessil PR160-violet LED as described in the “Workflow” section described in the SI. The temperature of the reaction was maintained at approximately 25 °C via a fan. After the reaction time, the mixture was diluted with AcOEt (10 mL) and washed with brine (3 × 10 mL). The organic layer was dried over Na_2_SO_4_, filtered and concentrated under reduced pressure. The crude mixture was purified by flash column chromatography.

#### General Procedure for the Photoinduced Synthesis of 4,4-difluorophenylbutanediols (**23**–**25**)

*Step 1*: To a flame-dried 4 mL vial equipped with a magnetic stir bar, redox active ether **1b** (227.0 mg, 1.0 mmol, 2.0 equiv), the corresponding styrene (0.5 mmol, 1.0 equiv) and 4DPAIPN (7.9 mg, 0.01 mmol, 0.02 equiv) were added, and the vial was subjected to 3 cycles of vacuum/argon degassing. Subsequently, 2.5 mL of dry DMSO was added under inert atmosphere and the solution was degassed with Argon for 30 s. The reaction mixture was irradiated for 16 h with a 427 nm Kessil PR160-violet LED as described in the “Workflow” section. The temperature of the reaction was maintained at approximately 25 °C via a fan. After the reaction time, the mixture was diluted with AcOEt (10 mL) and washed with brine (3 × 10 mL). The organic layer was dried over Na_2_SO_4_, filtered and concentrated under reduced pressure. The crude mixture was used in the second step without further purification.

*Step 2*: Into a 5 mL round-bottom flask the crude mixture form *Step 1* was dissolved in 0.6 mL of EtOH. Simultaneously, NaBH_4_ (60.0 mg, 1.6 mmol, 12.8 equiv) was suspended in drops of H_2_O in an Erlenmeyer. Then, the suspension was slowly added to the initial mixture. The reaction is monitored by thin layer chromatography. Upon completion of the reaction, 5 mL of aqueous solution of NaOH 1 M was added to the mixture and then diluted with 5 mL of Et_2_O. The organic layer was separated and the aqueous layer was further extracted with Et_2_O (3 × 5 mL). The combined organic layer was dried over Na_2_SO_4_ and evaporated under reduced pressure. The crude was purified by flash column chromatography through silica gel.

#### 4-(4,4,4-Trifluoro-3-hydroxybutanoyl)phenyl Acetate (**3**)

Compound **3** was prepared according to the general procedure from styrene **2a** (81.1 mg, 0.5 mmol). After purification by column chromatography through silica gel (hexane/EtOAc, 7.5:2.5, *R*_f_ = 0.48) a slightly orange solid was obtained. Compound **3** coelutes with phthalimide. Subsequently, the product was dissolved in toluene, filtrated, and evaporated. The title compound **3** was obtained as a white solid (85.6 mg, 0.31 mmol, 62% yield) with 4% impurity, **mp**: 74–76 °C. ^**1**^**H NMR** (600 MHz, CDCl_3_), δ (ppm): 8.00 (d, *J* = 8.7 Hz, 2H), 7.23 (d, *J* = 8.7 Hz, 2H), 4.70–4.67 (m, 1H), 3.61 (bs, 1H), 3.37 (dd, *J* = 17.7, 9.5 Hz, 1H), 3.27 (dd, *J* = 17.7, 2.4 Hz, 1H), 2.33 (s, 3H); ^**13**^**C{**^**1**^**H} NMR** (126 MHz, CDCl_3_), δ (ppm): 196.3, 168.8, 155.3, 133.7, 130.1 (2C), 124.9 (q, *J* = 279.5 Hz), 122.3 (2C), 67.2 (q, *J* = 30.0 Hz), 38.3, 21.2; ^**19**^**F{**^**1**^**H} NMR** (377 MHz, CDCl_3_), δ (ppm): – 79.3; **IR** (ATR) ν (cm^–1^): 3400, 2921, 2851, 1764, 1679, 1601, 1582, 1505, 1370, 1343, 1273; **HRMS** (ESI+) *m*/*z*: [M + Na]^+^ Calcd for C_12_H_11_F_3_O_4_Na 299.0501; found 299.0496.

#### 1-(4-(*Tert*-butyl)phenyl)-4,4,4-trifluoro-3-hydroxybutan-1-one (**4**)

Compound **4** was prepared according to the general procedure from styrene **2b** (80.1 mg, 0.5 mmol). After purification by column chromatography through silica gel (hexane/EtOAc, 9:1, *R*_f_ = 0.35), the title compound **4** was obtained as a white solid (78.2 mg, 0.29 mmol, 57% yield), **mp**: 73–75 °C. ^**1**^**H NMR** (400 MHz, CDCl_3_), δ (ppm): 7.91 (d, *J* = 8.5 Hz, 2H), 7.51 (d, *J* = 8.5 Hz, 2H), 4.71–4.65 (m, 1H), 3.66 (d, *J* = 4.0 Hz, 1H), 3.37 (dd, *J* = 17.7, 9.1 Hz, 1H) 3.29 (dd, *J* = 17.7, 2.7 Hz, 1H), 1.35 (s, 9H); ^**13**^**C{**^**1**^**H} NMR** (101 MHz, CDCl_3_), δ (ppm): 197.3, 158.2, 133.5, 128.2 (2C), 125.9 (2C), 124.8 (q, *J* = 281.8 Hz), 67.2 (q, *J* = 32.3 Hz), 38.0 (q, *J* = 1.9 Hz), 35.3, 31.0 (3C); ^**19**^**F{**^**1**^**H} NMR** (377 MHz, CDCl_3_), δ (ppm): – 79.2; **IR** (ATR) ν (cm^–1^): 3498, 2965, 2932, 2873, 1681, 1606, 1301, 1270; **HRMS** (ESI+) *m*/*z*: [M + Na]^+^ Calcd for C_14_H_17_F_3_O_2_Na 297.1073; found 297.1075.

#### 4,4,4-Trifluoro-3-hydroxy-1-(*p*-tolyl)butan-1-one (**5**)

Compound **5** was prepared according to the general procedure from styrene **2c** (49.0 mg, 0.4 mmol). After purification by column chromatography through silica gel (hexane/EtOAc, 9:1, *R*_f_ = 0.43), the title compound **5** was obtained as a white solid (76.6 mg, 0.33 mmol, 66% yield), **mp**: 93–95 °C. ^**1**^**H NMR** (500 MHz, CDCl_3_), δ (ppm): 7.87 (d, *J* = 7.9 Hz, 2H), 7.30 (d, *J* = 7.9 Hz, 2H), 4.70–4.66 (m, 1H), 3.59 (d, *J* = 4.5 Hz, 1H), 3.38–3.28 (m, 2H), 2.44 (s, 3H); ^**13**^**C{**^**1**^**H} NMR** (126 MHz, CDCl_3_), δ (ppm): 197.3, 145.4, 133.7, 129.7 (2C), 128.5 (2C), 124.9 (q, *J* = 278.8 Hz), 67.3 (q, *J* = 31.3 Hz), 38.1, 21.9; ^**19**^**F{**^**1**^**H} NMR** (282 MHz, CDCl_3_), δ (ppm): – 79.2; **IR** (ATR) ν (cm^–1^): 3370, 3035, 2957, 1681, 1608, 1573, 1403, 1341, 1314, 1276, 1153; **HRMS** (ESI+) *m*/*z*: [M + H]^+^ Calcd for C_11_H_12_F_3_O_2_ 233.0784; found 233.0785.

#### 1-([1,1′-Biphenyl]-4-yl)-4,4,4-trifluoro-3-hydroxybutan-1-one (**6**)

Compound **6** was prepared according to the general procedure from styrene **2d** (90.1 mg, 0.5 mmol). After purification by column chromatography through silica gel (hexane/EtOAc, 9:1, *R*_f_ = 0.3), the title compound **6** was obtained as a white solid (85.3 mg, 0.29 mmol, 58% yield), **mp**: 129–131 °C. ^**1**^**H NMR** (300 MHz, CDCl_3_), δ (ppm): 8.05 (d, *J* = 8.4 Hz, 2H), 7.73 (d, *J* = 8.4 Hz, 2H), 7.65–762 (m, 2H), 7.51–7.42 (m, 3H), 4.76–4.69 (m, 1H), 3.61 (d, *J* = 4.6 Hz, 1H), 3.43 (dd, *J* = 17.7, 8.8 Hz, 1H), 3.34 (dd, *J* = 17.7, 3.1 Hz, 1H). ^**13**^**C{**^**1**^**H} NMR** (126 MHz, CDCl_3_), δ (ppm): 197.3, 147.0, 139.7, 134.8, 129.2 (2C), 129.0 (2C), 128.7, 127.6 (2C), 127.5 (2C), 124.9 (q, *J* = 278.8 Hz), 67.3 (q, *J* = 32.5 Hz), 38.4; ^**19**^**F{**^**1**^**H} NMR** (282 MHz, CDCl_3_), δ (ppm): −79.2; **IR** (ATR) ν (cm^–1^): 3417, 3037, 2959, 1681, 1602, 1560, 1404, 1320, 1267; **HRMS** (ESI+) *m*/*z*: [M + Na]^+^ Calcd for C_16_H_14_F_3_O_2_Na 317.0760; found 317.0763.

#### 4,4,4-Trifluoro-3-hydroxy-1-(4-methoxyphenyl) butan-1-one (**7**)

Compound **7** was prepared according to the general procedure from styrene **2e** (67.1 mg, 0.5 mmol). After purification by column chromatography through silica gel (hexane/EtOAc, 8:2, *R*_f_ = 0.68), the title compound **7** was obtained as a yellow solid (67.0 mg, 0.27 mmol, 54% yield), **mp**: 98–101 °C. ^**1**^**H NMR** (400 MHz, CDCl_3_), δ (ppm): 7.95 (d, *J* = 8.0 Hz, 2H), 6.90 (d, *J* = 8.0 Hz, 2H), 4.69–4.64 (m, 1H), 3.89 (s, 1H), 3.74 (d, *J* = 4.6 Hz, 1H), 3.63–3.24 (m, 2H); ^**13**^**C{**^**1**^**H} NMR** (101 MHz, CDCl_3_), δ (ppm): 196.2, 164.4, 130.6 (2C), 124.8 (q, *J* = 280.8 Hz), 129.0, 114.1 (2C), 67.2 (q, *J* = 32.3 Hz), 55.6, 37.7 (q, *J* = 2.0 Hz); ^**19**^**F{**^**1**^**H} NMR** (377 MHz, CDCl_3_), δ (ppm): – 79.2; **IR** (ATR) ν (cm^–1^): 3372, 2962, 2921, 2846, 1673, 1549, 1348, 1282, 1265, 1234; **HRMS** (ESI+) *m*/*z*: [M + Na]^+^ Calcd for C_11_H_11_F_3_O_3_Na 271.0552; found 271.0559.

#### 4,4,4-Trifluoro-3-hydroxy-1-phenylbutan-1-one (**8**)

Compound **8** was prepared according to the general procedure from styrene **2f** (52 mg, 0.5 mmol). After purification by column chromatography through silica gel (hexane/EtOAc, 8:2, *R*_f_ = 0.58), the title compound **8** was obtained as a white solid (68.7 mg, 0.32 mmol, 63% yield), **mp**: 73–75 °C. ^**1**^**H NMR** (400 MHz, CDCl_3_), δ (ppm): 7.97 (d, *J* = 7.0 Hz, 2H), 7.63 (t, *J* = 7.4 Hz, 1H), 7.51 (dd, *J* = 7.4 Hz, 7.0 Hz, 2H), 4.73–4.67 (m, 1H), 3.54–3.60 (m, 1H), 3.40 (dd, *J* = 17.8, 9.1 Hz, 1H), 3.31 (dd, *J* = 17.8, 2.8 Hz, 1H); ^**13**^**C{**^**1**^**H} NMR** (101 MHz, CDCl_3_), δ (ppm): 197.6, 136.0, 134.2, 128.9 (2C), 128.2 (2C), 124.8 (*J* = 281.8 Hz), 67.1 (*J* = 31.3 Hz), 38.2; ^**19**^**F{**^**1**^**H} NMR** (377 MHz, CDCl_3_), δ (ppm): – 79.3; **IR** (ATR) ν (cm^–1^): 3381, 3063, 3032, 2922, 2853, 1683, 1275, 1225; **HRMS** (ESI+) *m*/*z*: [M + Na]^+^ Calcd for C_10_H_9_F_3_O_2_Na 241.0447; found 241.0444.

#### 4,4,4-Trifluoro-3-hydroxy-1-(naphthalen-2-yl)butan-1-one (**9**)

Compound **9** was prepared according to the general procedure from styrene **2g** (77.0 mg, 0.5 mmol). After purification by column chromatography through silica gel (hexane to hexane/EtOAc 8:2; *R*_f_ for hexane/EtOAc, 9.5:0.5 = 0.28), the title compound **9** was obtained as a white solid (89.8 mg, 0.34 mmol, 67% yield), **mp**: 75–77 °C. ^**1**^**H NMR** (600 MHz, CDCl_3_), δ (ppm): 8.46 (d, *J* = 1.8 H, 1H), 8.00 (dd, *J* = 8.6 Hz, 1.8 Hz, 1H), 7.97 (d, *J* = 8.2 Hz, 1H), 7.91–7.88 (m, 2H), 7.64 (t, *J* = 7.5 Hz, 1H), 7.58 (t, *J* = 7.5 Hz, 2H), 4.79–4.76 (m, 1H), 3.79 (d, *J* = 4.3 Hz, 1H), 3.54 (dd, *J* = 17.6, 9.6 Hz, 1H), 3.43 (dd, *J* = 17.6, 4.3 Hz, 1H); ^**13**^**C{**^**1**^**H} NMR** (151 MHz, CDCl_3_), δ (ppm): 197.6, 136.1, 133.5, 132.5, 130.5, 129.9, 129.2, 128.9, 128.0, 127.3, 125.0 (q, *J* = 279.0 Hz), 123.5, 67.2 (q, *J* = 31.5 Hz), 38.4; ^**19**^**F{**^**1**^**H} NMR** (282 MHz, CDCl_3_), δ (ppm): – 79.1; **IR** (ATR) ν (cm^–1^): 3374, 3055, 2915, 1682, 1628, 1598, 1470, 1390, 1336, 1274; **HRMS** (ESI+) *m*/*z*: [M + H]^+^ Calcd for C_14_H_12_F_3_O_2_ 269.0784; found 269.0788.

#### 4,4,4-Trifluoro-1-(2-fluoro-4-methylphenyl)-3-hydroxybutan-1-one (**10**)

Compound **10** was prepared according to the general procedure from styrene **2h** (68.0 mg, 0.5 mmol). After purification by column chromatography through silica gel (hexane/EtOAc, 9:1, *R*_f_ = 0.38), the title compound **10** was obtained as a white solid (56.3 mg, 0.23 mmol, 45% yield), **mp**: 59–61 °C. ^**1**^**H NMR** (500 MHz, CDCl_3_), δ (ppm): 7.32 (tq, *J* = 8.0 Hz, 5.9 Hz, 1H), 7.05 (d, *J* = 7.6 Hz, 1H), 6.97 (t, *J* = 9.3 Hz, 1H), 4.68–4.65 (m, 1H), 3.24–3.21 (m, 3H), 2.35 (s, 3H); ^**13**^**C{**^**1**^**H} NMR** (126 MHz, CDCl_3_), δ (ppm): 200.7, 160.6 (d, *J* = 247.5 Hz), 139.1 (d, *J* = 3.8 Hz), 132.2 (d, *J* = 10.0 Hz), 127.2 (d, *J* = 2.5 Hz), 127.1, 124.8 (q, *J* = 279.9 Hz), 113.6 (d, *J* = 22.5 Hz), 67.2 (q, *J* = 32.5 Hz), 44.5 (d, *J* = 5.0 Hz), 19.8 (d, *J* = 2.5 Hz); ^**19**^**F{**^**1**^**H} NMR** (282 MHz, CDCl_3_), δ (ppm): – 79.5 (s, 3F), – 114.2 (s, 1F); **IR** (ATR) ν (cm^–1^): 3433, 2981, 2937, 1694, 1614, 1572, 1441, 1394, 1301, 1271, 1248; **HRMS** (ESI+) *m*/*z*: [M + H]^+^ Calcd for C_11_H_11_F_4_O_2_ 251.0690; found 251.0691.

#### 4,4,4-Trifluoro-1-(3-fluorophenyl)-3-hydroxybutan-1-one (**11**)

Compound **11** was prepared according to the general procedure from styrene **2i** (61.1 mg, 0.5 mmol). After purification by column chromatography through silica gel (hexane/EtOAc, 8:2, *R*_f_ = 0.48), the title compound **11** was obtained as a white solid (41.3 mg, 0.17 mmol, 35% yield), **mp**: 76–78 °C. ^**1**^**H NMR** (500 MHz, CDCl_3_), δ (ppm): 7.78 (dt, *J* = 7.8, 1.3 Hz, 1H), 7.68 (ddd, *J* = 9.2, 2.6, 1.6 Hz, 1H), 7.52 (td, *J* = 7.8, 5.5 Hz, 1H), 7.38–7.35 (m, 1H), 4.77–4.70 (m, 1H), 3.43–3.38 (m, 2H), 3.30 (dd, *J* = 17.8, 2.5 Hz, 1H); ^**13**^**C{**^**1**^**H} NMR** (126 MHz, CDCl_3_), δ (ppm): 196.3 (d, *J* = 2.5 Hz), 163.1 (d, *J* = 247.5 Hz), 138.2 (d, *J* = 6.3 Hz), 130.8 (d, *J* = 7.5 Hz), 124.8 (q, *J* = 278.8 Hz), 124.2 (d, *J* = 3.8 Hz), 121.4 (d, *J* = 22.5 Hz), 115.1 (d, *J* = 22.5 Hz), 67.1 (q, *J* = 31.3 Hz), 38.7; ^**19**^**F{**^**1**^**H} NMR** (377 MHz, CDCl_3_), δ (ppm): – 79.3 (s, 3F), – 111.0 (s, 1F); **IR** (ATR) ν (cm^–1^): 3389, 3081, 2918, 1683, 1587, 1487, 1445, 1336, 1313, 1274, 1249; **HRMS** (ESI+) *m*/*z*: [M + H]^+^ Calcd for C_10_H_9_F_4_O_2_ 237.0533; found 237.0541.

#### 4,4,4-Trifluoro-1-(4-fluorophenyl)-3-hydroxybutan-1-one (**12**)

Compound **12** was prepared according to the general procedure from styrene **2j** (61.1 mg, 0.5 mmol). After purification by column chromatography through silica gel (hexane/EtOAc, 9:1, *R*_f_ = 0.28), the title compound **12** was obtained as a white solid (67.3 mg, 0.29 mmol, 57% yield), **mp**: 77–80 °C. ^**1**^**H NMR** (400 MHz, CDCl_3_), δ (ppm): 8.00 (dd, *J* = 8.9, 5.3 Hz, 2H), 7.17 (t, *J* = 8.9 Hz, 2H), 4.74–4.65 (m, 1H), 3.54 (d, *J* = 8.0 Hz, 1H), 3.37 (dd, *J* = 17.7, 9.6 Hz, 1H), 3.26 (dd, *J* = 17.7, 2.8 Hz, 1H); ^**13**^**C{**^**1**^**H} NMR** (101 MHz, CDCl_3_), δ (ppm): 195.8, 166.4 (d, *J* = 257.6 Hz), 132.5, 131.8 (d, *J* = 9.1 Hz, 2C), 124.7 (q, *J* = 281.8 Hz), 116.1 (d, *J* = 22.2 Hz, 2C), 67.0 (q, *J* = 32.3 Hz), 38.2; ^**19**^**F{**^**1**^**H} NMR** (377 MHz, CDCl_3_), δ (ppm): – 79.3 (3F), – 100.1 (1F); **IR** (ATR) ν (cm^–1^): 3391, 2921, 2853, 1687, 1594, 1342, 1275, 1224; **HRMS** (ESI+) *m*/*z*: [M + Na]^+^ Calcd for C_10_H_8_F_4_O_2_Na 259.0352; found 259.0358.

#### 1-(4-Chlorophenyl)–4,4,4-trifluoro-3-hydroxybutan-1-one (**13**)

Compound **13** was prepared according to the general procedure from the corresponding styrene **2k** (69.3 mg, 0.5 mmol). After purification by column chromatography through silica gel (hexane/EtOAc, 9:1, *R*_f_ = 0.25), the title compound **13** was obtained as a white solid (51.8 mg, 0.21 mmol, 41% yield), **mp**: 91–93 °C. ^**1**^**H NMR** (500 MHz, CDCl_3_), δ (ppm): 7.91 (d, *J* = 8.6 Hz, 2H), 7.48 (d, *J* = 8.6 Hz, 2H), 4.72–4.67 (m, 1H), 3.48 (d, *J* = 4.7 Hz, 1H), 3.37 (dd, *J* = 17.7, 9.5 Hz, 1H), 3.26 (dd, *J* = 17.7, 2.4 Hz, 1H); ^**13**^**C{**^**1**^**H} NMR** (126 MHz, CDCl_3_), δ (ppm): 196.3, 140.9, 134.5, 129.7 (2C), 129.4 (2C), 124.9 (q, *J* = 278.8 Hz), 67.1 (q, *J* = 31.3 Hz), 38.4; ^**19**^**F{**^**1**^**H} NMR** (282 MHz, CDCl_3_), δ (ppm): – 79.3; **IR** (ATR) ν (cm^–1^): 3374, 2926, 2856, 1685, 1591, 1571, 1490, 1399, 1256, 1158; **HRMS** (ESI+) *m*/*z*: [M + H]^+^ Calcd for C_10_H_9_ClF_3_O_2_ 253.0238; found 253.0246.

#### 1-(4-Bromophenyl)-4,4,4-trifluoro-3-hydroxybutan-1-one (**14**)

Compound **14** was prepared according to the general procedure from styrene **2l** (91.5 mg, 0.5 mmol). After purification by column chromatography through silica gel (hexane/EtOAc, 9:1, *R*_f_ = 0.25), the title compound **14** was obtained as a white solid (89.1 mg, 0.30 mmol, 60% yield), **mp**: 111–114 °C. ^**1**^**H NMR** (400 MHz, CDCl_3_), δ (ppm): 7.83 (d, *J* = 8.7 Hz, 2H), 7.63 (d, *J* = 8.70 Hz, 2H), 4.66–4.72 (m, 1H), 3.40 (d, *J* = 4.7 Hz, 1H), 3.36 (dd, *J* = 17.8, 9.3 Hz, 1H), 3.26 (dd, *J* = 17.8, 2.6 Hz, 1H); ^**13**^**C{**^**1**^**H} NMR** (101 MHz, CDCl_3_), δ (ppm): 196.5, 134.9, 132.4 (2C), 129.8 (2C), 129.0, 124.8 (q, *J* = 280.8 Hz), 67.1 (q, *J* = 32.3 Hz), 38.4 (*J* = 1.0 Hz); ^**19**^**F{**^**1**^**H} NMR** (377 MHz, CDCl_3_), δ (ppm): −79.3; **IR** (ATR) ν (cm^–1^): 3389, 2948, 2924, 2853, 1684, 1587, 1343, 1312, 1277, 1222; **HRMS** (ESI+) *m*/*z*: [M + Na]^+^ Calcd for C_10_H_8_BrF_3_O_2_Na 318.9552; found 318.9547.

#### 2-(2,2,2-Trifluoro-1-hydroxyethyl)-2,3-dihydro-1*H*-inden-1-one (**15**)

Compound **15** was prepared according to the general procedure from styrene **2m** (58.0 mg, 0.5 mmol). After purification by column chromatography through silica gel (hexane/EtOAc, 9:1, *R*_f_ = 0.25), the title compound **15** was obtained as a white solid (48.3 mg, 0.21 mmol, 42% yield), **mp**: 63–65 °C. The compound **15** was formed by a mixture of partially separable diastereomers in a 6:1 ratio as determined by ^1^H NMR. ^**1**^**H NMR** (400 MHz, CDCl_3_), δ (ppm): 7.80 (d, 0.15 × 1, *J* = 7.7 Hz, 1H), 7.75 (d, 0.85 × 1, *J* = 7.7 Hz, 1H), 7.62 (t, *J* = 8.0 Hz, 1H), 7.58 (d, 0.15 × 1, *J* = 8.0 Hz, 1H), 7.52 (d, 0.85 × 1, *J* = 8.0 Hz, 1H), 7.37 (t, *J* = 8.0 Hz, 1H), 4.86–4.82 (m, 1H), 3.50 (dd, 0.85 × 1, *J* = 17.7, 5.4 Hz, 1H), 3.23 (dd, *J* = 17.7, 8.3 Hz, 1H), 3.06 (d, *J* = 5.7 Hz, 1H), 2.97–3.01 (m, 1H), 2.49 (dd, 0.15 × 1, *J* = 17.3, 3.9 Hz, 1H); ^**13**^**C{**^**1**^**H} NMR** (126 MHz, CDCl_3_), δ (ppm): 207.6, 205.3, 154.9, 153.9, 137.0, 136.0, 135.7, 135.3, 127.8, 127.7, 126.8, 125.3 (q, *J* = 281.3 Hz), 124.3, 124.2, 68.5 (q, *J* = 32.5 Hz), 47.7, 47.2, 28.4, 26.7; ^**19**^**F{**^**1**^**H} NMR** (377 MHz, CDCl_3_), δ (ppm): −77.4; **IR** (ATR) ν (cm^–1^): 3476, 2955, 2922, 1702, 1606, 1586, 1467, 1435, 1389, 1332, 1275, 1252; **HRMS** (ESI+) *m*/*z*: [M + Na]^+^ Calcd for C_11_H_9_F_3_O_2_Na 253.0447; found 253.0443.

#### 2-(2,2,2-Trifluoro-1-hydroxyethyl)-3,4-dihydronaphthalen-1(2*H*)-one (**16**)

Compound **16** was prepared according to the general procedure from styrene **2n** (65.0 mg, 0.5 mmol). After purification by column chromatography through silica gel (hexane/EtOAc, 9.5:0.5, *R*_f_ = 0.23), the title compound **16** was obtained as a white solid (59.8 mg, 0.25 mmol, 49% yield), **mp**: 80–82 °C. The compound **16** was formed by a mixture of partially separable diastereomers in a 2:1 ratio as determined by ^1^H and ^19^F NMR. ^**1**^**H NMR** (500 MHz, CDCl_3_), δ (ppm): 8.04 (d, 1 × 0.63, *J* = 7.5 Hz, 1H), 7.52 (t, 1 × 0.65, *J* = 7.5 Hz, 1H), 7.33 (t, 1 × 0.65, *J* = 7.5 Hz, 1H), 7.27–7.08 (m, 2H), 6.65 (s, 0.36 × 1, 1H), 5.06–5.02 (m, 0.65 × 1, 1H), 4.62–4.59 (m, 0.37 × 1, 1H), 3.10–3.01 (m, 1H), 2.92–2.83 (m, 2H), 2.49–2.27 (m, 2H); ^**13**^**C{**^**1**^**H} NMR** (126 MHz, CDCl_3_), δ (ppm): 197.2, 144.3, 135.6, 134.3, 133.1, 133.0, 132.1, 129.0, 128.1, 127.8, 127.6, 127.0, 126.9, 126.8, 125.6 (q, *J* = 280.0 Hz, major), 124.5 (q, *J* = 281.3 Hz, minor), 73.9 (q, *J* = 31.3 Hz, minor), 68.4 (q, *J* = 31.3 Hz, major), 48.4, 28.8, 27.9, 23.1, 22.7; ^**19**^**F{**^**1**^**H} NMR** (282 MHz, CDCl_3_), δ (ppm): – 75.4 (s, 0.64 × 1, 3F), – 77.0 (s, 0.36 × 1, 3F); **IR** (ATR) ν (cm^–1^): 3363, 3072, 2969, 2924, 1680, 1599, 1486, 1454, 1430, 1357, 1327, 1306, 1273, 1231; **HRMS** (ESI+) *m*/*z*: [M + H]^+^ Calcd for C_12_H_12_F_3_O_2_ 245.0784; found 245.0788.

#### 1-(Benzo[*b*]thiophen-5-yl)-4,4,4-trifluoro-3-hydroxybutan-1-one (**17**)

Compound **17** was prepared according to the general procedure from styrene **2o** (80.0 mg, 0.5 mmol). After purification by column chromatography through silica gel (hexane/EtOAc, 9:1, *R*_f_ = 0.25), the title compound **17** was obtained as a white solid (80.9 mg, 0.30 mmol, 59% yield), **mp**: 73–75 °C. ^**1**^**H NMR** (500 MHz, CDCl_3_), δ (ppm): 8.43 (d, *J* = 1.7 Hz, 1H), 7.97–7.92 (m, 2H), 7.57 (d, *J* = 5.5 Hz, 1H), 7.46 (d, *J* = 5.5 Hz, 1H), 4.77–4.73 (m, 1H), 3.73 (d, *J* = 4.4 Hz, 1H), 3.48 (dd, *J* = 17.6, 9.4 Hz, 1H), 3.40 (dd, *J* = 17.6, 2.5 Hz, 1H); ^**13**^**C{**^**1**^**H} NMR** (126 MHz, CDCl_3_), δ (ppm): 197.5, 145.4, 139.6, 132.7, 128.5, 125.0 (q, *J* = 278.8 Hz), 124.8, 124.5, 123.2, 123.1, 67.2 (q, *J* = 31.3 Hz), 38.4; ^**19**^**F{**^**1**^**H} NMR** (282 MHz, CDCl_3_), δ (ppm): −79.2; **IR** (ATR) ν (cm^–1^): 3416, 3109, 3083, 2916, 1682, 1588, 1421, 1328, 1307, 1264, 1216; **HRMS** (ESI+) *m*/*z*: [M + H]^+^ Calcd for C_12_H_10_F_3_O_2_S 275.0348; found 275.0350.

#### 1-(Benzo[*c*]thiophen-1-yl)-4,4,4-trifluoro-3-hydroxybutan-1-one (**18**)

Compound **18** was prepared according to the general procedure from styrene **2p** (80.1 mg, 0.5 mmol). After purification by column chromatography through silica gel (hexane/EtOAc, 9:1, *R*_f_ = 0.28), the title compound **18** was obtained as a yellow solid (65.8 mg, 0.24 mmol, 48% yield), **mp**: 116–118 °C. ^**1**^**H NMR** (500 MHz, CDCl_3_), δ (ppm): 8.04 (s, 1H), 7.92 (d, *J* = 8.1 Hz, 1H), 7.89 (d, *J* = 8.1 Hz, 1H), 7.50 (t, *J* = 7.7 Hz, 1H), 7.44 (t, *J* = 7.7 Hz, 1H), 4.71–4.74 (m, 1H), 3.52 (d, *J* = 4.9 Hz, 1H), 3.46 (dd, *J* = 17.2, 9.6 Hz, 1H), 3.34 (dd, *J* = 17.2, 2.5 Hz, 1H); ^**13**^**C{**^**1**^**H} NMR** (126 MHz, CDCl_3_), δ (ppm): 191.7, 143.0, 142.5, 139.0, 130.7, 128.3, 126.4, 125.5, 124.8 (q, *J* = 280.0 Hz), 123.2, 67.2 (q, *J* = 31.3 Hz), 38.9; ^**19**^**F{**^**1**^**H} NMR** (282 MHz, CDCl_3_), δ (ppm): – 79.3; **IR** (ATR) ν (cm^–1^): 3350, 2955, 2924, 1654, 1593, 1509, 1427, 1408, 1334, 1297, 1274; **HRMS** (ESI+) *m*/*z*: [M + H]^+^ Calcd for C_12_H_10_F_3_O_2_S 275.0348; found 275.0351.

#### 4,4,4-Trifluoro-3-hydroxy-1-(isobenzofuran-1-yl)butan-1-one (**19**)

Compound **19** was prepared according to the general procedure from styrene **2q** (73.0 mg, 0.5 mmol). After purification by column chromatography through silica gel (hexane/EtOAc, 9:1, *R*_f_ = 0.23), the title compound **19** was obtained as a white solid (45.2 mg, 0.18 mmol, 35% yield), **mp**: 81–83 °C. ^**1**^**H NMR** (600 MHz, CDCl_3_), δ (ppm): 7.74 (d, *J* = 7.9 Hz, 1H), 7.62–7.59 (m, 2H), 7.53 (ddd, *J* = 8.4, 7.1, 1.3 Hz, 1H), 7.35 (t, *J* = 7.1 Hz, 1H), 4.75–4.71 (m, 1H), 3.45–3.39 (m, 2H), 3.31 (dd, *J* = 17.5 Hz, 2.5 Hz, 1H); ^**13**^**C{**^**1**^**H} NMR** (126 MHz, CDCl_3_), δ (ppm): 188.0, 156.1, 151.9, 129.2, 127.0, 124.8 (q, *J* = 278.8 Hz), 124.4, 123.7, 114.4, 112.7, 67.0 (q, *J* = 32.5 Hz), 38.7; ^**19**^**F{**^**1**^**H} NMR** (282 MHz, CDCl_3_), δ (ppm): – 79.4; **IR** (ATR) ν (cm^–1^): 3441, 3335, 3113, 3074, 2922, 2853, 1650, 1637, 1610, 1550, 1478, 1450, 1373, 1330, 1305, 1275, 1215; **HRMS** (ESI+) *m*/*z*: [M + Na]^+^ Calcd for C_12_H_9_F_3_O_3_Na 281.0396; found 281.0398.

#### 4-(4,4,4-Trifluoro-3-hydroxybutanoyl)benzyl 2-(4-isobutylphenyl)propanoate (**20**)

Compound **20** was prepared according to the general procedure from styrene **2r** (161.0 mg, 0.5 mmol). After purification by column chromatography through silica gel (hexane/EtOAc, 7.5:2.5, *R*_f_ = 0.68), the title compound **20** was obtained as a yellow solid (111.3 mg, 0.25 mmol, 51% yield), **mp**: 72–74 °C. Compound **20** was formed as mixture of partially separable diastereomers in a 10:1 ratio as determined in the crude mixture by ^19^F NMR. During isolation, the minor diastereomer was eluted with the impurities, and the yield of the product was determined considering the mass of the major diastereomer only. ^**1**^**H NMR** (400 MHz, CDCl_3_), δ (ppm): 7.87 (d, *J* = 8.4 Hz, 2H), 7.28 (d, *J* = 8.4 Hz, 2H), 7.21–7.19 (m, 2H), 7.10 (d, *J* = 8.1 Hz, 2H), 5.17 (q, *J* = 13.5 Hz, 2H), 4.69–4.67 (m, 1H), 3.79 (q, *J* = 7.1 Hz, 1H), 3.50 (d, *J* = 4.6 Hz, 1H), 3.35 (dd, *J* = 17.8, 9.1 Hz, 1H), 3.27 (dd, *J* = 17.8, 2.8 Hz, 1H), 2.47 (d, *J* = 7.2 Hz, 2H), 1.84 (sept, *J* = 7.1 Hz, 1H), 1.53 (d, *J* = 7.1 Hz, 3H), 0.91 (d, *J* = 7.1 Hz, 6H); ^**13**^**C{**^**1**^**H} NMR** (126 MHz, CDCl_3_), δ (ppm): 197.1, 174.5, 142.8, 141.0, 137.5, 135.6, 129.6 (2C), 128.5 (2C), 127.7 (2C), 127.4 (2C), 124.9 (q, *J* = 278.8 Hz), 67.2 (q, *J* = 31.3 Hz), 65.3, 45.3, 45.2, 38.4, 30.4, 22.5 (2C), 18.4; ^**19**^**F{**^**1**^**H} NMR** (377 MHz, CDCl_3_), δ (ppm): −79.1 (0.05 × 1, 3F), −79.3 (1 × 0.95, 3F); **IR** (ATR) ν (cm^–1^): 3537, 2955, 2869, 1720, 1681, 1609, 1512, 1460, 1419, 1379, 1305, 1275; **HRMS** (ESI+) *m*/*z*: [M + Na]^+^ Calcd for C_24_H_27_F_3_O_4_Na 459.1754; found 459.1738.

#### 4-(4,4,4-Trifluoro-3-hydroxybutanoyl) phenyl 4-([1,1′-biphenyl]-4-yl)-4-oxobutanoate (**21**)

Compound **21** was prepared according to the general procedure from styrene **2s** (185.2 mg, 0.5 mmol). After purification by column chromatography through silica gel (hexane/EtOAc, 7.5:2.5, *R*_f_ = 0.39). The title compound **21** was obtained as a yellow solid (126.0 mg, 0.26 mmol, 52% yield), **mp**: 130–133 °C.^**1**^**H NMR** (400 MHz, CDCl_3_), δ (ppm): 8.06 (d, *J* = 8.4 Hz, 2H), 7.96 (d, *J* = 8.4 Hz, 2H), 7.70 (d, *J* = 8.4 Hz, 2H), 7.63 (d, *J* = 7.0 Hz, 2H), 7.49 (m, 4H), 7.41 (t, *J* = 7.3 Hz, 1H), 5.23 (s, 2H), 4.71–4.67 (m, 1H), 3.48 (s, 1H), 3.42–3.26 (m, 4H), 2.88 (t, *J* = 6.5 Hz, 2H); ^**13**^**C{**^**1**^**H} NMR** (126 MHz, CDCl_3_), δ (ppm) 197.5, 197.0, 172.6, 146.1, 142.5, 139.8, 135.6, 135.2, 129.0 (2C), 128.7 (2C), 128.5 (2C), 128.3 (2C), 128.0, 127.3 (4C), 124.7 (q, *J* = 225.2 Hz), 66.9 (q, *J* = 25.3 Hz), 65.5, 38.3, 31.3, 28.3; ^**19**^**F{**^**1**^**H} NMR** (377 MHz, CDCl_3_), δ (ppm): −79.2; **IR** (ATR) ν (cm^–1^): 3367, 2920, 2850, 1740, 1684, 1323, 1262, 1224, 1206; **HRMS** (ESI+) *m*/*z*: [M + Na]^+^ Calcd for C_27_H_23_F_3_O_5_Na 507.1390; found 507.1390.

#### 4-(4,4,4-Trifluoro-3-hydroxybutanoyl)benzyl 2-(3-fluoro-[1,1′-biphenyl]-4-yl)propanoate (**22**)

Compound **22** was prepared according to the general procedure from styrene **2t** (180.2 mg, 0.5 mmol). After purification by column chromatography through silica gel (hexane/EtOAc 9:1–7:3; *R*_f_ for hexane/EtOAc, 7:3 = 0.75) the title compound **22** was obtained as a colorless oil (116.2 mg, 0.24 mmol, 49% yield). The compound **22** was formed by a mixture of partially separable diastereomers in a 3.5:1 ratio as determined by ^1^H and ^19^F NMR (isolated in a 6:1 ratio). ^**1**^**H NMR** (600 MHz, CDCl_3_), δ (ppm): 7.93 (d, 0.87 × 1, *J* = 8.3 Hz, 2H), 7.55 (d, *J* = 8.3 Hz, 2H), 7.46 (t, *J* = 8.3 Hz, 2H), 7.43–7.37 (m, 4H), 7.15 (d, *J* = 8.5 Hz, 1H), 7.12 (d, *J* = 8.5 Hz, 1H), 5.22 (d, 0.87 × 1, *J* = 6.0 Hz, 2H), 5.15 (d, 0.13 × 1, *J* = 6.0 Hz, 2H), 4.71–4.68 (m, 1H), 3.85 (q, *J* = 6.0 Hz, 1H), 3.47 (bs, 0.87 × 1, 1H), 3.39–3.27 (m, 2H), 1.59–1.56 (d, *J* = 12.0 Hz, 4H); ^**13**^**C{**^**1**^**H} NMR** (151 MHz, CDCl_3_), δ (ppm): 197.1, 173.7, 159.8 (d, *J* = 247.5 Hz), 142.4, 141.5 (d, *J* = 7.5 Hz), 135.8, 135.5, 131.0 (d, *J* = 3.2 Hz), 129.1 (d, *J* = 3.0 Hz), 128.7 (2C), 128.6 (2C), 128.0 (2C), 127.9 (2C), 124.9 (q, *J* = 279.0 Hz), 123.7 (d, *J* = 3.0 Hz), 115.4 (d, *J* = 23.8 Hz), 67.2 (q, *J* = 33.0 Hz), 66.4, 65.8, 45.1, 38.4, 18.4; ^**19**^**F{**^**1**^**H} NMR** (282 MHz, CDCl_3_), δ (ppm): – 79.1 (0.13 × 3, 3F), – 79.2 (0.87 × 3, 3F), – 117.4 (0.87 × 1, 1F), – 117.6 (0.13 × 1, 1F); **IR** (ATR) ν (cm^–1^): 3465, 3034, 2981, 2933, 1734, 1685, 1611, 1581, 1515, 1484, 1417, 1379, 1326, 1270, 1222; **HRMS** (ESI+) *m*/*z*: [M + Na]^+^ Calcd for C_26_H_22_F_4_O_4_Na 497.1346; found 497.1356.

#### 4,4-Difluoro-1-(4-fluorophenyl)butane-1,3-diol (**23**)

Compound **23** was prepared according to the general procedure from styrene **2j** (61.1 mg, 0.5 mmol). After purification by column chromatography through silica gel (hexane/EtOAc, 3:1; *R*_f_ for hexane/EtOAc 4:1 = 0.1), the title compound **23** was obtained as a colorless oil (33.4 mg, 0.15 mmol, 30% yield). Compound **23** was formed as mixture of partially separable diastereomers in a 4.5:1 ratio as determined in the crude mixture by ^1^H NMR. ^**1**^**H NMR** (400 MHz, CDCl_3_), δ (ppm): 7.34 (dd, *J* = 8.5, 5.5 Hz, 2H), 7.05 (t, *J* = 8.7 Hz, 2H), 5.70 (td, 0.80 × 1, *J* = 56.0, 4.0 Hz, 1H), 5.67 (td, 0.20 × 1, *J* = 56.0, 4.0 Hz, 1H), 5.09 (dd, 0.80 × 1, *J* = 8.7, 3.4 Hz, 1H), 4.99 (dd, 0.20 × 1, *J* = 8.7, 3.4 Hz, 1H), 4.17–3.90 (m, 1H), 2.91 (br s, 1H), 2.50 (br s, 1H), 2.06–1.86 (m, 2H); ^**13**^**C{**^**1**^**H} NMR** (101 MHz, CDCl_3_), δ (ppm): 162.5 (d, *J* = 246.1 Hz), 139.6, 127.3 (d, *J* = 8.1 Hz, 2C), 116.2 (t, *J* = 244.0 Hz), 115.7 (d, *J* = 21.4 Hz, 2C), 70.4, 68.7 (t, *J* = 23.9 Hz), 38.1 (t, *J* = 2.5 Hz); ^**19**^**F{**^**1**^**H} NMR** (377 MHz, CDCl_3_), δ (ppm): −114.5, −129.7, −129.8; **IR** (ATR) ν (cm^–1^): 3365, 2959, 2925, 2856, 1604, 1509, 1493, 1222, 1156; **HRMS** (ESI+) *m*/*z*: [M + Na]^+^ Calcd for C_10_H_11_F_3_O_2_Na 243.0603; found 243.0606.

#### 4,4-Difluoro-1-(4-methoxyphenyl)butane-1,3-diol (**24**)

Compound **24** was prepared according to the general procedure from styrene **2e** (77.1 mg, 0.5 mmol). After purification by column chromatography through silica gel (hexane/EtOAc, 3:1–2:1; *R*_f_ for hexane/EtOAc 4:1 = 0.1), the title compound **24** was obtained as a colorless oil (57.4 mg, 0.25 mmol, 49% yield). Compound **24** was formed as mixture of partially separable diastereomers in a 3:1 ratio as determined in the crude mixture by ^1^H NMR. ^**1**^**H NMR** (500 MHz, CDCl_3_), δ (ppm): 7.29 (d, *J* = 8.7 Hz, 2H), 6.90 (d, *J* = 8.7 Hz, 2H), 5.70 (td, *J* = 56.0, 4.1 Hz, 1H), 5.06 (dd, 0.89 × 1, *J* = 8.9, 3.2 Hz, 1H), 4.98–4.80 (m, 0.10 × 1, 1H), 4.13–4.01 (m, 1H), 3.81 (s, 3H), 3.61 (s, 0.09 × 1, 1H), 2.98 (d, *J* = 5.1 Hz, 0.85 × 1, 1H), 2.61 (s, 0.09 × 1, 1H), 2.35 (s, 0.80 × 1, 1H), 2.02 (ddd, *J* = 14.6, 8.9, 3.0 Hz, 1H), 1.93 (ddd, *J* = 14.6, 9.1, 3.3 Hz, 1H); ^**13**^**C{**^**1**^**H} NMR** (126 MHz, CDCl_3_), δ (ppm): 159.4, 135.8, 126.9 (2C), 116.3 (t, *J* = 243.8 Hz), 114.2 (2C), 70.8, 68.8 (t, *J* = 23.8 Hz), 55.5, 38.0 (t, *J* = 3.1 Hz); ^**19**^**F{**^**1**^**H} NMR** (377 MHz, CDCl_3_), δ (ppm): −129.70; **IR** (ATR) ν (cm^–1^): 3386, 2959, 2933, 2839, 1611, 1511, 1302, 1245, 1175, 1142; **HRMS** (ESI+) *m*/*z*: [M + Na]^+^ Calcd for C_11_H_14_F_2_O_3_Na 255.0803; found 255.0803.

#### 4,4-Difluoro-1-(naphthalen-2-yl)butane-1,3-diol (**25**)

Compound **25** was prepared according to the general procedure from styrene **2g** (77.1 mg, 0.5 mmol). After purification by column chromatography through silica gel (hexane/EtOAc, 3:1–2:1; *R*_f_ for hexane/EtOAc 4:1 = 0.1), the title compound **25** was obtained as a colorless oil (27.7 mg, 0.11 mmol, 22% yield), **mp**: 43–45 °C. Compound **25** was formed as mixture of partially separable diastereomers in a 4.5:1 ratio as determined in the crude mixture by ^1^H NMR. ^**1**^**H NMR** (500 MHz, CDCl_3_), δ (ppm): 7.89–7.80 (m, 4H), 7.55–7.42 (m, 3H), 5.71 (td, 0.80 × 1, *J* = 56.0, 4.2 Hz, 1H), 5.69 (td, 0.20 × 1, *J* = 56.0, 4.2 Hz, 1H), 5.27 (dd, 0.80 × 1, *J* = 10.0, 5.0 Hz, 1H), 5.16 (dd, 0.20 × 1, *J* = 10.0, 5.0 Hz, 1H), 4.13–4.06 (m, 1H), 3.03 (br s, 0.80 × 1, 1H), 2.91 (s, 0.20 × 1, 1H), 2.85 (s, 0.20 × 1, 1H), 2.66 (s, 0.80 × 1, 1H), 2.08 (qdd, *J* = 15.0, 10.0, 5.0 Hz, 2H); ^**13**^**C{**^**1**^**H} NMR** (126 MHz, CDCl_3_), δ (ppm): 141.1, 133.4, 133.2, 128.7, 128.1, 127.9, 126.5, 126.2, 124.4, 123.7, 116.3 (t, *J* = 244.0 Hz), 71.3, 68.7 (t, *J* = 23.8 Hz), 37.9; ^**19**^**F{**^**1**^**H} NMR** (377 MHz, CDCl_3_), δ (ppm): −129.68; **IR** (ATR) ν (cm^–1^): 3303, 3056, 2923, 1386, 1195, 1140, 1104; **HRMS** (ESI+) *m*/*z*: [M + Na]^+^ Calcd for C_14_H_14_F_2_O_2_Na 275.0854; found 275.0863.

## Data Availability

The data underlying this study are available in the published article and its Supporting Information.

## Supporting Information Available

Preparation of starting materials, reaction workflow, isolation of compound **26**, large-scale synthesis, mechanistic studies, NMR spectra and monocrystal X-ray diffraction (PDF)

## References

[ref1] aDhungana R. K.; KC S.; Basnet P.; Giri R. Transition Metal-Catalyzed Dicarbofunctionalization of Unactivated Olefins. Chem. Rec. 2018, 18, 1314–1340. 10.1002/tcr.201700098. 29517841

[ref2] aPitzer L.; Schwarz J. L.; Glorius F. Reductive radical-polar crossover: traditional electrophiles in modern radical reactions. Chem. Sci. 2019, 10, 8285–8291. 10.1039/C9SC03359A. 32055300 PMC7003961

[ref3] aFuruya T.; Kamlet A.; Ritter T. Catalysis for fluorination and trifluoromethylation. Nature 2011, 473, 470–477. 10.1038/nature10108. 21614074 PMC3119199

[ref4] aPurser S.; Moore P. R.; Swallow S.; Gouverneur V. Fluorine in Medicinal Chemistry. Chem. Soc. Rev. 2008, 37, 320–330. 10.1039/B610213C. 18197348

[ref5] aKoike T.; Akita M. New Horizons of Photocatalytic Fluoromethylative Difunctionalization of Alkenes. Chem 2018, 4, 409–437. 10.1016/j.chempr.2017.11.004.

[ref6] aCarboni A.; Dagousset G.; Magnier E.; Masson G. Photoredox-Induced Three-Component Oxy-, Amino-, and Carbotrifluoromethylation of Enecarbamates. Org. Lett. 2014, 16, 1240–1243. 10.1021/ol500374e. 24520865

[ref7] aLiu X.; Xu C.; Wang M.; Liu Q. Trifluoromethyltrimethylsilane: Nucleophilic Trifluoromethylation and Beyond. Chem. Rev. 2015, 115, 683–730. 10.1021/cr400473a. 24754488

[ref8] aCharpentier J.; Früh N.; Togni A. Electrophilic Trifluoromethylation by Use of Hypervalent Iodine Reagents. Chem. Rev. 2015, 115, 650–682. 10.1021/cr500223h. 25152082

[ref9] aShao Y.-M.; Yang W.-B.; Kuo T.-H.; Tsai K.-C.; Lin C.-H.; Yang A.-S.; Liang P.-H.; Wong C.-H. Design, synthesis, and evaluation of trifluoromethyl ketones as inhibitors of SARS-CoV 3CL protease. Bioorg. Med. Chem. 2008, 16, 4652–4660. 10.1016/j.bmc.2008.02.040. 18329272 PMC7127754

[ref10] aWouters J.; Moureau F.; Evrard G.; Koenig J.-J.; Jegham S.; George P.; Durant F. A reversible monoamine oxidase a inhibitor, befloxatone: structural approach of its mechanism of action. Bioorg. Med. Chem. 1999, 7, 1683–1693. 10.1016/S0968-0896(99)00102-9. 10482460

[ref11] aPrakash G. K. S.; Krishnamurti R.; Olah G. A. Synthetic methods and reactions. 141. Fluoride-induced trifluoromethylation ofcarbonyl compounds with trifluoromethyltrimethylsilane (TMS-CF_3_). A trifluoromethide equivalent. J. Am. Chem. Soc. 1989, 111, 393–395. 10.1021/ja00183a073.

[ref12] Kelly C. B.; Mercadante M. A.; Leadbeater N. E. Trifluoromethyl ketones: properties, preparation, and application. Chem. Commun. 2013, 49, 11133–11148. 10.1039/c3cc46266h. 24162741

[ref13] aZhang J.; Liu D.; Liu S.; Ge Y.; Lan Y.; Chen Y. Visible-Light-Induced Alkoxyl Radicals Enable α-C(sp^3^)-H Bond Allylation. iScience 2020, 23, 100755 10.1016/j.isci.2019.100755. 31884167 PMC6941871

[ref14] Shu C.; Noble A.; Aggarwal V. K. Metal-free photoinduced C(sp^3^)–H borylation of alkanes. Nature 2020, 586, 714–720. 10.1038/s41586-020-2831-6. 33116286

[ref15] Lombardi L.; Cerveri A.; Giovanelli R.; Reis M. C.; López C. S.; Bertuzzi G.; Bandini M. Direct Synthesis of α-Aryl-α-Trifluoromethyl Alcohols via Nickel Catalyzed Cross-Electrophile Coupling. Angew. Chem., Int. Ed. 2022, 61, e202211732 10.1002/anie.202211732. PMC9828748 36161744

[ref16] aChen F.; Xu X.-H.; Chu L.; Qing F.-L. Visible-Light-Induced Nickel-Catalyzed Radical Cross-Couplings to Access α-Aryl-α-trifluoromethyl Alcohols. Org. Lett. 2022, 24, 9332–9336. 10.1021/acs.orglett.2c03943. 36484514

[ref17] aHe B.-Q.; Yu X.-Y.; Wang P.-Z.; Chen J.-R.; Xiao W.-J. A photoredox catalyzed iminyl radical-triggered C–C bond cleavage/addition/Kornblum oxidation cascade of oxime esters and styrenes: synthesis of ketonitriles. Chem. Commun. 2018, 54, 12262–12265. 10.1039/C8CC07072E. 30318535

[ref18] aJiang Q.; Guo T.; Wu K.; Yu Z. Rhodium(III)-catalyzed sp^2^ C–H bond addition to CF_3_-substituted unsaturated ketones. Chem. Commun. 2016, 52, 2913–2915. 10.1039/C5CC10361D. 26781559

[ref19] See Supporting Information.

[ref20] aErickson J. A.; McLoughlin J. I. Hydrogen Bond Donor Properties of the Difluoromethyl Group. J. Org. Chem. 1995, 60, 1626–1631. 10.1021/jo00111a021.

[ref21] aYerien D. E.; Barata-Vallejo S.; Postigo A. Difluoromethylation Reactions of Organic Compounds. Chem. - Eur. J. 2017, 23, 14676–14701. 10.1002/chem.201702311. 28632338

[ref22] Li Y.; Guo S.; Li Q.; Zheng K. Metal-free photoinduced C(sp^3^)-H/C(sp^3^)-H cross-coupling to access α-tertiary amino acid derivatives. Nat. Commun. 2023, 14, 6225 10.1038/s41467-023-41956-6. 37802984 PMC10558569

[ref23] Wayner D. D. M.; McPhee D. J.; Griller D. Oxidation and reduction potentials of transient free radicals. J. Am. Chem. Soc. 1988, 110, 132–137. 10.1021/ja00209a021.

