# Supplementary material for: Direct Synthesis of 2-Hydroxytrifluoroethylacetophenones via Organophotoredox-Mediated Net-Neutral Radical/Polar Crossover
Source: J Org Chem. 2024 Aug 1;89(16):11682–92. doi: 10.1021/acs.joc.4c01419 (PMC11334190; doi:10.1021/acs.joc.4c01419)
Supplement: Supplementary file 1 — jo4c01419_si_001.pdf [file jo4c01419_si_001.pdf]

Supporting Information

Direct Synthesis of 2-Hydroxytrifluoroethylacetophenones via Organophotoredox-Mediated Net-Neutral Radical/Polar Crossover

Albert Gallego-Gamo,[a] Pau Sarró,[a] Yingmin Ji,[a] Roser Pleixats,[a] Elies Molins,[b] Carolina Gimbert-Suriñach,[a,]* Adelina Vallribera[a,]* and Albert Granados[a,]*

[a] *Departament de Química and Centro de Innovación en Química Avanzada (ORFEO-CINQA), Universitat Autònoma de Barcelona, Cerdanyola del Vallès, 08193 Barcelona, Spain*

[b] *Institut de Ciència de Materials de Barcelona (ICMAB-CSIC), Campus UAB, 08193, Bellaterra, Spain*

*To whom correspondence should be addressed.

carolina.gimbert@uab.es adelina.vallribera@uab.es albert.granados@uab.es

TABLE OF CONTENT

1. List of Used Styrenes and Redox Active Ethers S3

2. Synthesis of Starting Materials S4

3. Reaction Workflow and Isolation of Compound **26** S8

4. Large-scale Synthesis of compound **4**.. S10

5. Mechanistic Investigation S11

6. Data of X-Ray Structure of Compound **11**... S17

7. NMR Spectra... S22

1. List of Used Styrenes and Redox Active Ethers

Styrenes **2a-n** are commercially available. Styrenes **2o-q** and **2r-t** were prepared following modified reported procedures.[1]

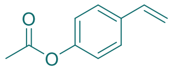
2a

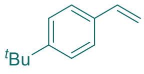
2b

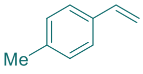
2c

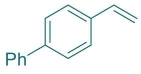
2d

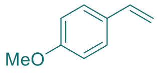
2e

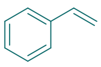
2f

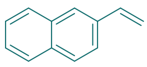
2g

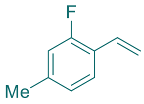
2h

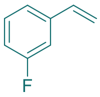
2i

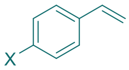
2j (X = F)
2k (X = Cl)
2l (X = Br)

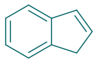
2m

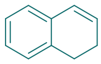
2n

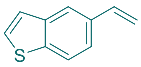
2o

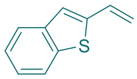
2p

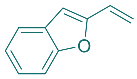
2q

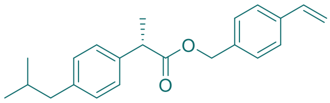
2r
from Ibuprofen

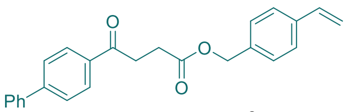
2s
from Fenbufen

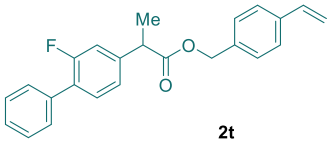
2t
from Flurbiprofen

Redox active ethers (**1a-1b**) were prepared following modified reported procedures.[2]

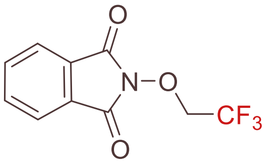
1a

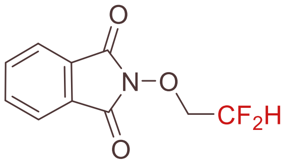
1b

---

[1] a) Molander, G. A.; Brown, A. R. Suzuki-Miyaura Cross-Coupling Reactions of Potassium Vinyltrifluoroborate with Aryl and Heteroaryl Electrophiles. *J. Org. Chem.* **2006**, *71*, 9681–9686. b) Marcum, J. S.; Cervarich, T. N.; Manan, R. S.; Roberts, C. C.; Meek, S. J. (CDC)-Rhodium-Catalyzed Hydroallylation of Vinylarenes and 1,3-Dienes with AllylTrifluoroborates. *ACS Catal.* **2019**, *9*, 5881–5889. c) Granados, A.; Dhungana, R. K.; Sharique, M.; Molander, G. From Styrenes to Fluorinated Benzyl Bromides: A Photoinduced Difunctionalization via Atom Transfer Radical Addition. *Org. Lett.* **2022**, *24*, 4750–4755.

[2] a) Shu, C.; Noble, A.; Aggarwal, V. K. Metal-free photoinduced C(sp^3)-H borylation of alkanes. *Nature* **2020**, *586*, 714–719. b) Li, Y.; Guo, S.; Li, Q.; Zheng, K. Metal-free photoinduced C(sp^3)-H/C(sp^3)-H cross-coupling to access α-tertiary amino acid derivatives *Nat. Commun.* **2023**, *14*, 6225.

2. Synthesis of Starting Materials

2.1. Synthesis of Styrenes

Synthesis of Styrene 2o[1a]

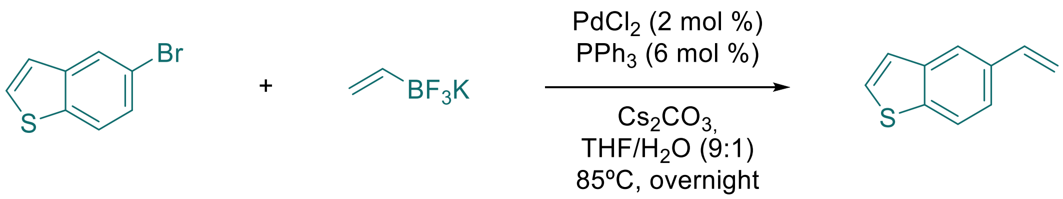

In a Schlenk flask, potassium vinyltrifluoroborate (134 mg, 1.00 mmol, 1 equiv), $PdCl_2$ (3.5 mg, 0.02 mmol, 0.02 equiv), PPh_3 (16 mg, 0.06 mmol, 0.06 equiv), Cs_2CO_3 (978 mg, 3.00 mmol, 3 equiv), and 5-bromobenzo[*b*]thiophene (1.00 mmol, 1 equiv) were dissolved in 2 mL of THF/water (9:1) and heated at 85°C. The reaction mixture was stirred overnight. Upon completion it was cooled down to rt and diluted with water (5 mL), followed by extraction with CH_2Cl_2 (3 x 15 mL). The organic layer was dried over Na_2SO_4, filtered, and concentrated under reduced pressure. The crude mixture was purified by flash column chromatography to yield **2o** in 79% yield (126 mg, 0.79 mmol).

Method B (Styrenes 2p and 2q)[1b]

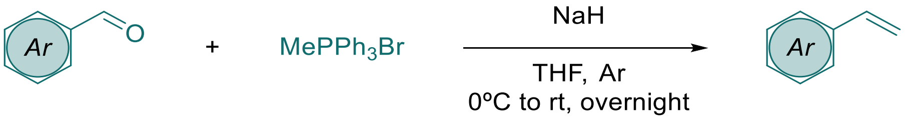

In a Schlenk flask, the corresponding aldehyde (1.00 mmol, 1 equiv) and methyltriphenylphosphonium bromide (428 mg, 1.2 mmol, 1.2 equiv) were dissolved in dry THF (5 mL) at 0ºC. To the stirring suspension, sodium hydride (108 mg, 4.5 mmol, 4.5 equiv) was added and the mixture was allowed to warm up to room temperature and proceed overnight. Upon completion, the reaction mixture was quenched with water (10 mL) and washed with brine (3 x 10 mL). The organic layer was dried over Na_2SO_4, filtered, and concentrated under reduced pressure. The crude mixture was purified by flash column chromatography.

Method C (Styrenes 2r-2t)[1a]

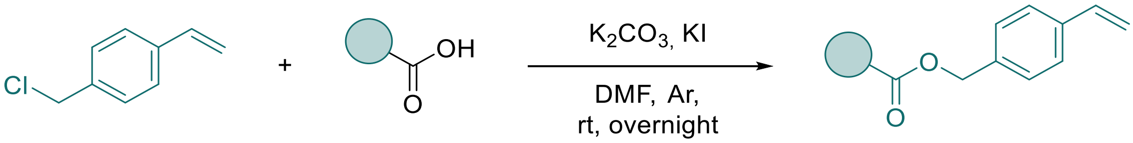

In a Schlenk flask, the corresponding carboxylic acid (1.0 mmol, 1 equiv) was dissolved in dry DMF (5 mL). Then, K_2CO_3 (207.3 mg, 1.5 mmol, 1.5 equiv) and KI (249 mg, 1.5 mmol, 1.5 equiv) was added and stirred. To the stirring suspension, 4-vinylbenzyl chloride (167.8 mg, 1.1 mmol, 1.1 equiv) was added, and the reaction was allowed to proceed overnight at room temperature. After the reaction time, the mixture was diluted with 10 mL of AcOEt and 10 mL of water. The reaction mixture was then extracted and washed with brine (3 x 10 mL), dried over Na_2SO_4, filtered and concentrated under reduced pressure. The crude mixture was purified by flash column chromatography.

2.2. *Synthesis of Redox Active Ethers*

Synthesis of 1a[2a]

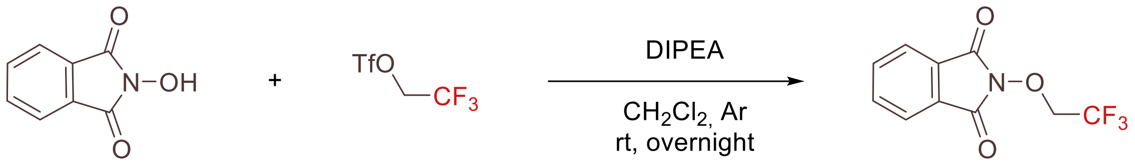

1a

Prepared following a modified procedure from Aggarwal.[2a] In a 100 mL Schlenk flask containing a solution of *N*-hydroxyphthalimide (1.63 g, 10.0 mmol, 1 equiv) in CH_2Cl_2 (30 mL), DIPEA (2.58 g, 20.0 mmol, 2 equiv) and 2,2,2-trifluoroethyl trifluoromethanesulphonate (2.55 g, 11.0 mmol, 1.1 equiv) were added and kept stirring at room temperature overnight. Subsequently, the reaction was quenched with water (30 mL) and extracted with CH_2Cl_2 (3 x 40 mL). The organic layers were combined and washed with brine (30 mL), dried over Na_2SO_4, filtered, and concentrated under reduced pressure. The residue was purified by column chromatography through silica gel (hexane: ethyl acetate, 6:4, R_f = 0.75) to yield **1a** as a white solid (2.07 g, 8.44 mmol, 85% yield). **^{1}H NMR** (500 MHz, $CDCl_3$), δ (ppm): 7.92 – 7.83 (m, 2H), 7.82 – 7.75 (m, 2H), 4.55 (q, *J* = 8.0 Hz, 2H). **^{13}C NMR** (101 MHz, $CDCl_3$), δ (ppm): 162.6 (2C), 135.0 (2C), 128.7 (2C), 124.0 (2C), 122.5 (q, *J* = 279.5 Hz), 73.2 (q, *J* = 35.2 Hz). **^{19}F{^{1}H} NMR** (377 MHz, $CDCl_3$), δ (ppm): -73.4.

Synthesis of 1b[2b]

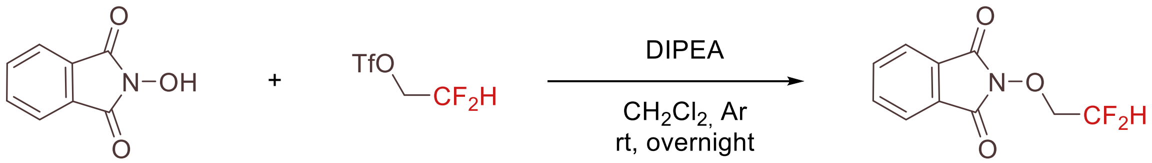

1b

Prepared following a modified procedure from Zheng.[2b] In a 100 mL Schlenk flask containing a solution of *N*-hydroxyphthalimide (0.70 g, 4.3 mmol, 1 equiv) in CH_2Cl_2 (12 mL), DIPEA (1.11 g, 8.6 mmol, 2 equiv) and 2,2-difluoroethyl trifluoromethanesulphonate (0.62 mL, 4.7 mmol, 1.1 equiv) were added and kept stirring at room temperature overnight. Subsequently, the reaction was quenched with water (10 mL) and extracted with CH_2Cl_2 (3 x 15 mL). The organic layers were combined and washed with brine (10 mL), dried over Na_2SO_4, filtered and concentrated under reduced pressure. The residue was purified by column chromatography through silica gel (hexane: ethyl acetate, 6:4, R_f = 0.75) to yield **1b** as a white solid (0.87 g, 4.0 mmol, 90 % yield). **^{1}H NMR** (500 MHz, $CDCl_3$), δ (ppm): 7.93 – 7.82 (m, 2H), 7.82 – 7.71 (m, 2H), 6.22 (tt, *J* = 55.0, 5.0 Hz, 1H), 4.37 (td, *J* = 15.0, 5.0 Hz, 2H). **^{13}C NMR** (126 MHz, $CDCl_3$), δ (ppm): 163.1 (2C), 135.0 (2C), 128.8 (2C), 124.0 (2C), 113.3 (t, *J* = 242.3 Hz), 76.2 (t, *J* = 29.4 Hz). **^{19}F{^{1}H} NMR** (377 MHz, $CDCl_3$), δ (ppm): -124.5.

***2.3.** Synthesis of Organophotocatalysts*

The organophotoctalayst were prepared following modified reported procedures.[3]

Method A (4DPAIPN)

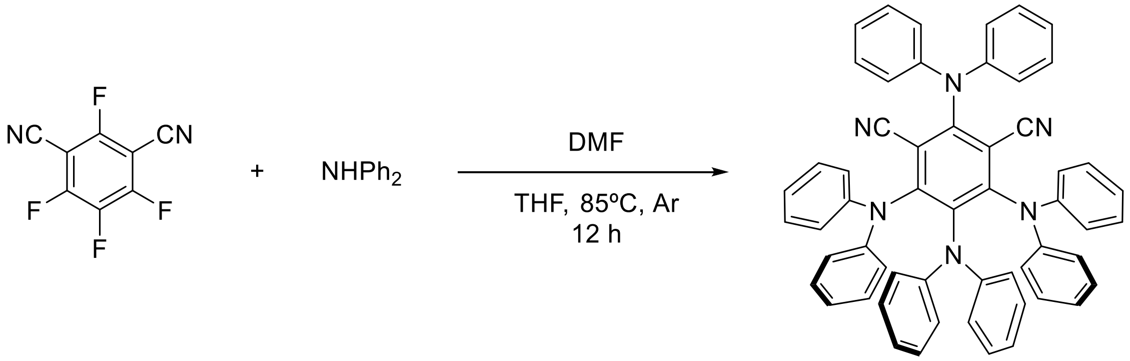

In a 100 mL 3 necked round bottom flask, NaH (60% in oil, 0.60 g, 15.0 mmol, 6.5 equiv) was washed and triturated with pentane under N_2 atmosphere. Meanwhile, in a Schlenk flask, diphenylamine (1.69 g, 10.0 mmol, 5.0 equiv) was dissolved in dry DMF (40 mL). After removing the pentane, the diphenylamine solution was slowly added to the washed NaH and heated at 85ºC for 30 minutes. Then, the mixture was cooled down to rt and tetrafluoroisophthalonitrile (0.40 g,

[3] Kwon, Y.; Lee, J.; Noh, Y.; Kim, D.; Lee, Y.; Yu, C.; Roldao, J. C.; Feng, S.; Gierschner, J.; Wannemacher, R.; Kwon, M. S. Formation and degradation of strongly reducing cyanoarene-based radical anions towards efficient radical anion-mediated photoredox catalysis. *Nat. Commun.* **2023**, *14*, 92.

2.0 mmol, 1 equiv) was added. Then, the reaction was allowed to proceed for 12 h at 85ºC. After completion of the reaction, 20 mL of water was added, and the product was extracted from the mixture with AcOEt. The organic phase was washed with brine (3 x 10 mL), dried over Na_2SO_4 and concentrated under reduced pressure. The crude mixture was purified by flash column chromatography. The spectroscopic data for the isolated compound agreed with the reported procedures.

Method B (4CzIPN)

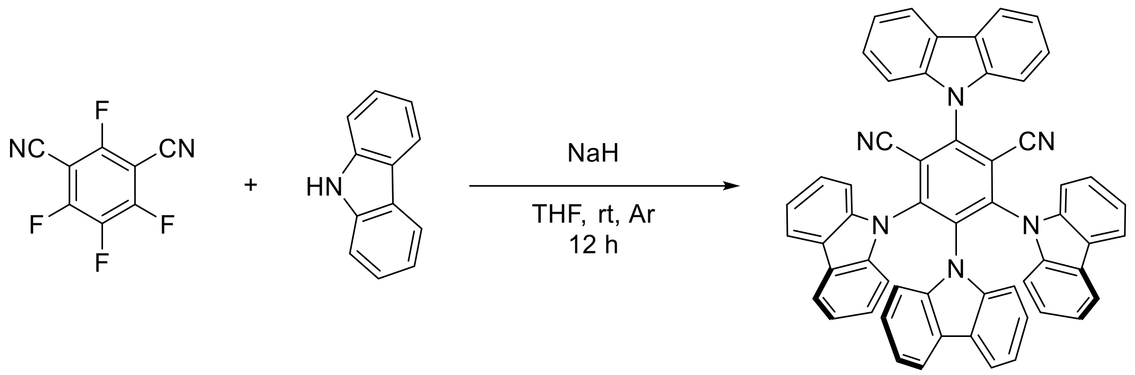

In a 100 mL 3 necked round bottom flask, NaH (60% in oil, 0.60 g, 15.0 mmol, 6.5 equiv) was washed and triturated with pentane under N_2 atmosphere. Meanwhile, in a Schlenk flask, carbazole (1.67 g, 10.0 mmol, 5.0 equiv) was dissolved in dry THF (40 mL). After removing the pentane, the carbazole solution was slowly added to the washed NaH. After 30 minutes, tetrafluoroisophthalonitrile (0.40 g, 2.0 mmol, 1.0 equiv) was added and the reaction was allowed to proceed 12 h at room temperature. After completion of the reaction, 20 mL of water was added, and the product was extracted from the mixture with AcOEt. The organic layer was dried over Na_2SO_4 and concentrated under reduced pressure. The product was purified by recrystallization. The spectroscopic data for the isolated compound was in agreement with the reported procedures.

3. Reaction Workflow and Isolation of Compound 26

3.1. *Reaction Workflow*

All photoinduced reactions were done using a Kessil PR160-violet LED lamp (30 W High Luminous DEX 2100 LED, λ_{max} = 427 nm). The LED was placed 3 cm away from the reaction vial within a ventilated fume hood and using a fan to maintain the temperature approximately at 25ºC.

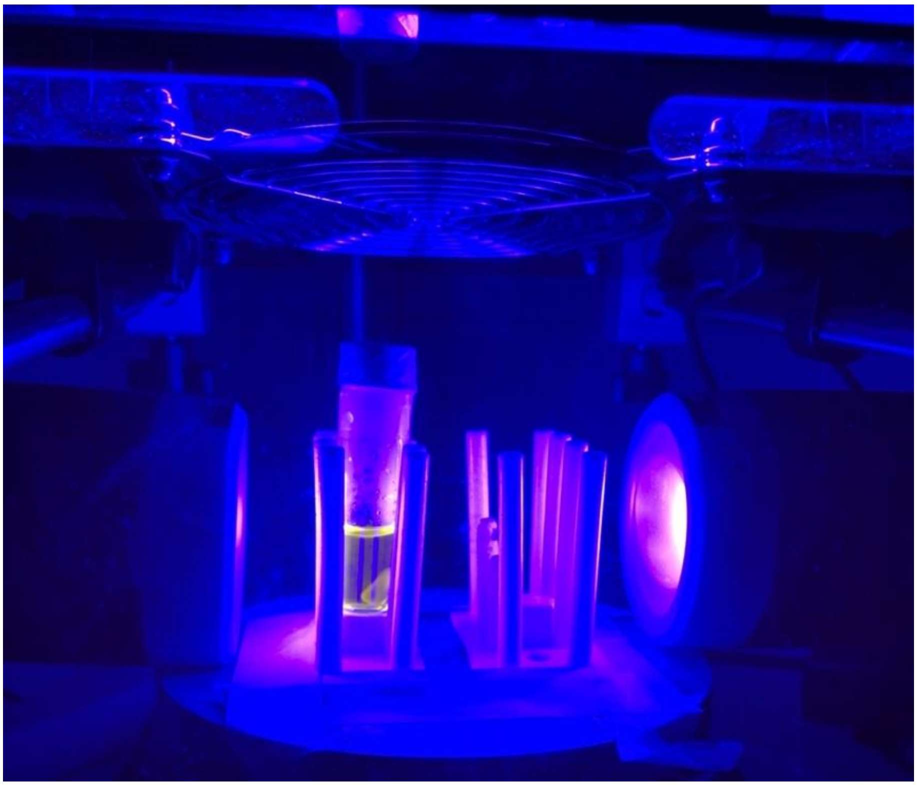

Figure S1. Reaction setup for the photoinduced synthesis of 2-hydroxytrifluoroethylacetophenones

3.2. *Isolation of compound* ***26***

4,4-Difluoro-3-hydroxy-1-(naphthalen-2-yl)butan-1-one (**26**)

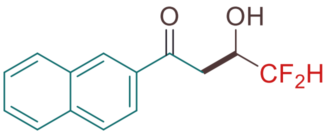

Compound **26** was prepared according to the general procedure (*Step 1*) from styrene **2g** (77.1 mg, 0.5 mmol). After purification by column chromatography through silica gel (hexane:EtOAc,

9:1 – 7:3; R_f for hexane:EtOAc, 9:1 = 0.15), the title compound **26** was degraded along the column and was obtained as a yellow solid (15.0 mg, 0.06 mmol, 12% yield), **mp**: 48 – 50ºC. Purification trials with other methods (*e. g.* Alumina gel) were unsuccessful. **^{1}H NMR** (500 MHz, $CDCl_3$), δ (ppm): 8.49 (d, *J* = 2.5 Hz, 1H), 8.02 (dd, *J* = 8.4, 2.5 Hz, 1H), 7.98 (d, *J* = 8.4 Hz, 1H), 7.90 (dd, *J* = 12.4, 8.4 Hz, 2H), 7.64 (t, *J* = 7.5 Hz, 1H), 7.58 (t, *J* = 7.5 Hz, 1H), 5.95 (td, *J* = 55.9, 3.4 Hz, 1H), 4.52 (ddt, *J* = 14.1, 10.1, 5.1 Hz, 1H), 3.54 – 3.38 (m, 3H); **^{13}C{^{1}H} NMR** (126 MHz, $CDCl_3$), δ (ppm): 198.9, 136.1, 133.7, 132.5, 130.5, 129.8, 129.1, 128.9, 128.0, 127.2, 123.5, 115.7 (dd, *J* = 244.4, 242.7 Hz), 68.1 (dd, *J* = 26.0, 23.8 Hz), 37.9 (t, *J* = 3.0 Hz); **^{19}F{^{1}H} NMR** (377 MHz, $CDCl_3$), δ (ppm): -128.41 (d, *J* = 288.1 Hz, 1F), -131.46 (d, *J* = 287.7 Hz, 1F); **IR** (ATR) ν (cm^{-1}): 3444, 2965, 2871, 1665, 1605, 1409, 1305, 1211, 1182, 1105; **HRMS** (ESI+) m/z: $[M+H]^+$ Calcd. for $C_{14}H_{13}F_2O_2$ 251.0878; found 251.0888.

4. Large-scale Synthesis of compound 4

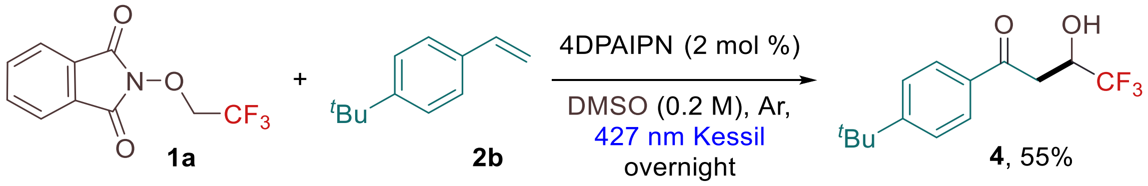

In a 25 mL Schlenk tube with a stirring bar, redox active ether **1a** (1.23 g, 5.0 mmol, 2.0 equiv), 1-(*tert*-butyl)-4-vinylbenzene **2b** (0.40 g, 2.5 mmol, 1.0 equiv), and 4DPAIPN (39.9 mg, 0.05 mmol, 0.02 equiv) were added, and the Schlenk was subjected to 3 cycles of vacuum/argon degassing. Subsequently, 12.5 mL of dry DMSO was added under inert atmosphere and the solution was degassed with Argon bubbling for 60 seconds. The reaction mixture was irradiated overnight with two 427 nm Kessil PR160-violet LEDs shown below. The temperature of the reaction was maintained at approximately 25 °C via a fan. After the reaction time, the mixture was diluted with AcOEt (40 mL) and washed with brine (3 x 30 mL). The organic layer was dried over Na_2SO_4, filtered and concentrated under reduced pressure. The crude mixture was purified by flash column chromatography to yield compound **4** (376 mg, 55%).

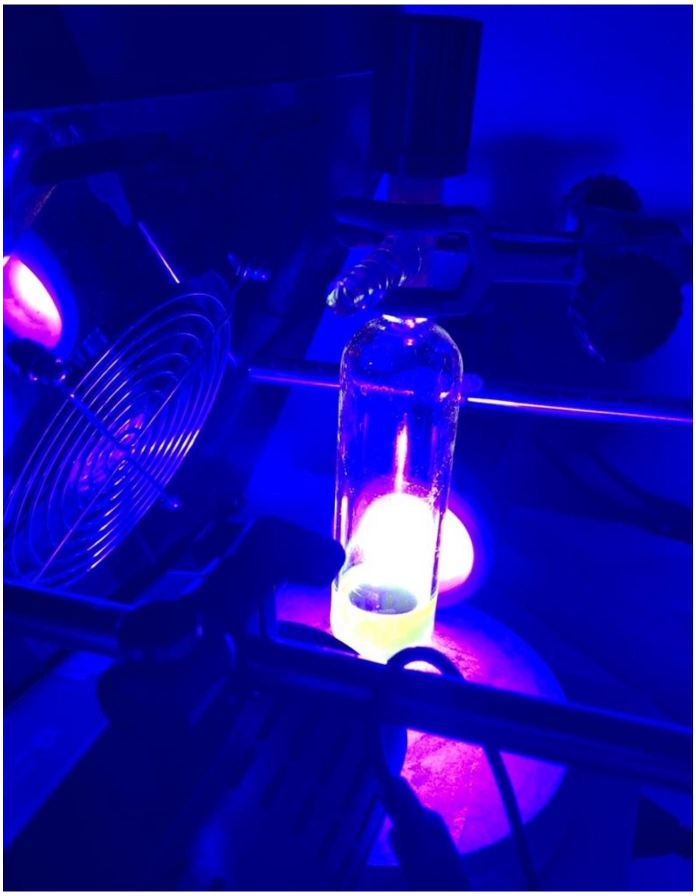

Figure S2. Reaction setup for the photoinduced and large-scale synthesis of **4**

5. Mechanistic Investigation

7.1. Cyclic Voltammetry Experiment and Redox Data

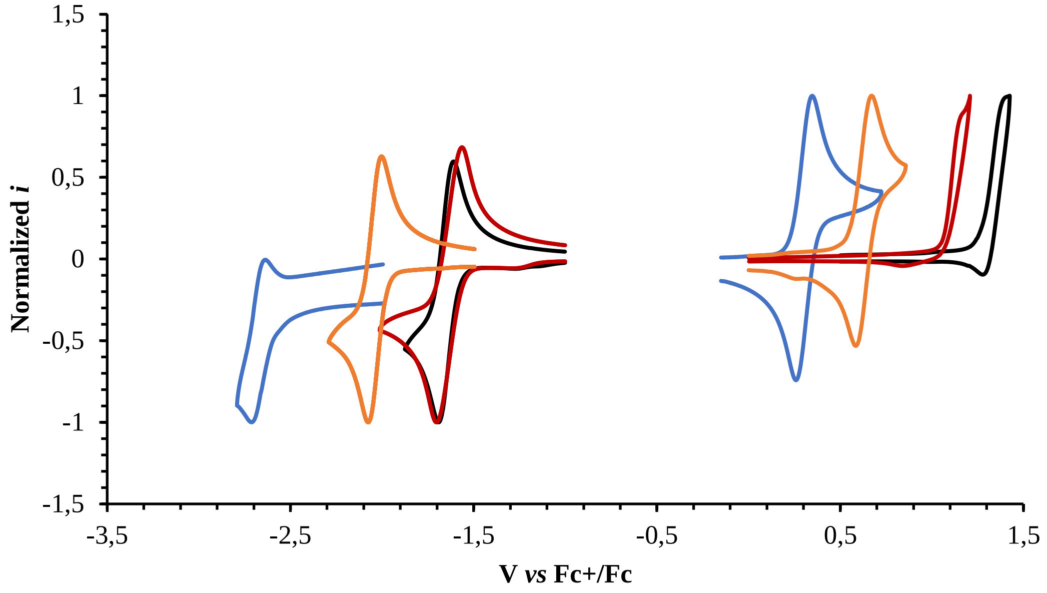

Figure S3: *Cyclic voltammetry of compounds* ***Ir(ppy)$_3$ (blue)****,* ***[Ir((dF)(CF$_3$)ppy)$_2$(dtbpy)]PF$_6$ (black)****,* ***4DPAIPN (orange)****,* ***4CzIPN (red)****. Conditions: Ir(ppy)$_3$ 2.3 mM (100 mV/s), [Ir((dF)(CF$_3$) ppy)$_2$(dtbpy)]PF$_6$ 2.7 mM (100 mV/s), 4DPAIPN 2.6 mM (100 mV/s), 4CzIPN 2.9 mM (100 mV/s) in MeCN, TBAPF$_6$ 0.1 M, rt. All measurements start at 0.0 V vs reference electrode. Scan direction to negative potentials for reductive scans and positive potentials for oxidative scans. Glassy carbon disk as working electrode, platinum wire as auxiliary electrode and SCE or AgNO$_3$/Ag as reference electrode. IUPAC plotting.*

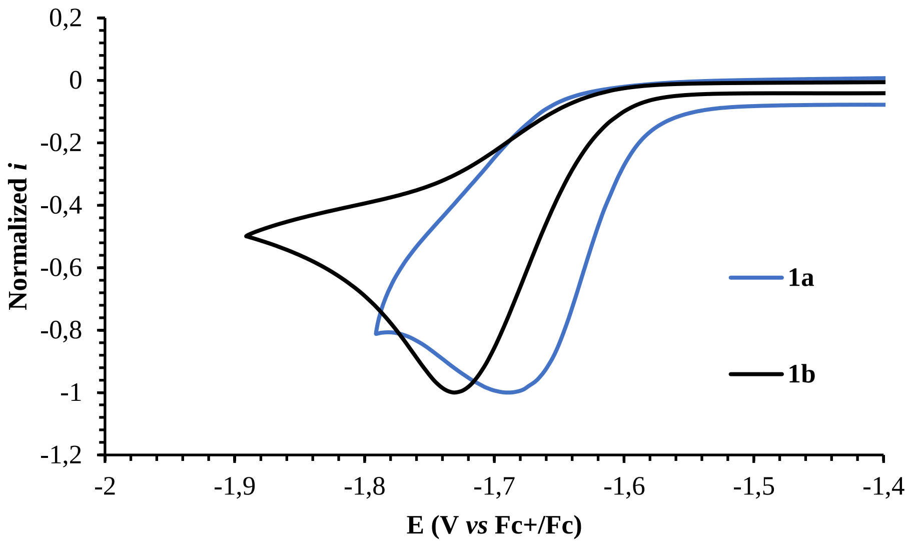

Figure S4: *Cyclic voltammetry of redox active ethers* ***1a (blue)*** *and* ***1b (black)****. Conditions:* ***1a*** *2.5 mM (100 mV/s),* ***1b*** *2.7 mM (100 mV/s), in MeCN, TBAPF$_6$ 0.1 M, rt. All measurements start at 0.0 V vs reference electrode. Scan direction to negative potentials for reductive scans. Glassy carbon disk as working electrode, platinum wire as auxiliary electrode and SCE as reference electrode. IUPAC plotting.*

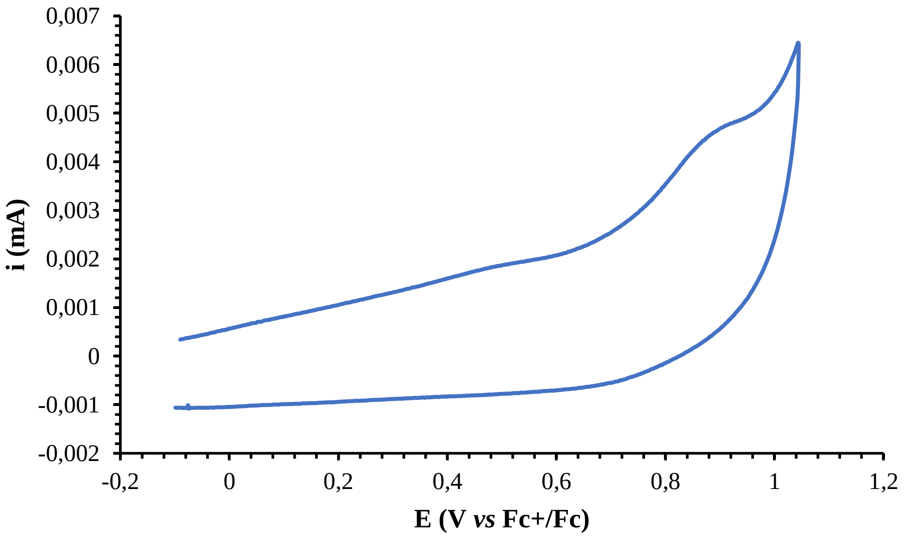

Figure S5: *Cyclic voltammetry of styrene* ***2a****. Conditions:* ***2a*** *2.2 mM (100 mV/s) in MeCN, TBAPF$_6$ 0.1 M, rt. The measurement starts at 0.0 V vs reference electrode. Scan direction to positive potentials for oxidative scans. Glassy carbon disk as working electrode, platinum wire as auxiliary electrode and* $AgNO_3$/Ag *as reference electrode. IUPAC plotting.*

***Table S1:** Collected redox data of the presented work relevant compounds.*

Compound	***$E_{1/2}$ (PC^+/PC)***	***$E_{1/2}$ (PC/PC^-)***	***E_a***	***E_c***	***E ($PC^+/^*PC$)***	***E ($^*PC/PC^-$)***
Ir(ppy)$_3$	0.32	-2.68	-	-	-2.45	0.09
[Ir((dF)(CF$_3$) ppy)$_2$(dtbpy)]PF$_6$	1.37	-1.65		-	-1.21	0.93
4DPAIPN	0.63	-2.04	-	-	-1.99	0.58
4CzIPN	-	-1.63	1.13	-	-1.57	1.07
1a	-	-	-	-1.63	-	-
1b	-	-	-	-1.67	-	-
2a	-	-	0.81	-	-	-

All values are given in V *vs* Fc^+/Fc. For those that presented irreversible waves, E_a (oxidation) or E_c (reduction) are extracted as an approximation of the thermodynamic $E_{1/2}$.

Calculus for the Excited State potentials were performed with the following equation:[4]

1) $E_{1/2}(PC^+/^*PC) = E_{1/2}\ (PC^+/PC) - E_{0\text{-}0}$
2) $E_{1/2}\ (^*PC/PC^-) = E_{1/2}\ (PC/PC^-) + E_{0\text{-}0}$
3) E (eV) = 1.2398/λ (in μm), then $E_{0\text{-}0}$ is when $\lambda = (\lambda_{max,abs} + \lambda_{max,em}) / 2$ (*Rehem-Weller equation)*

7.2. TEMPO Experiment

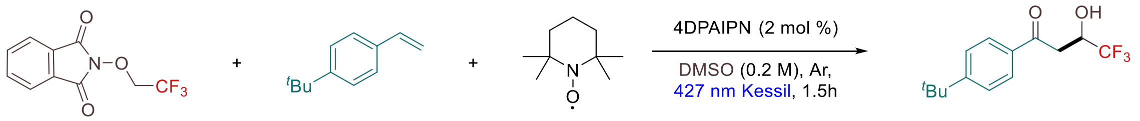

1a **2b** 5 equiv **4**, 0%

To a flame-dried 4 mL vial equipped with a magnetic stir bar, redox active ether **1a** (49.0 mg, 0.2 mmol, 2.0 equiv), 1-(*tert*-butyl)-4-vinylbenzene **2b** (16.0 mg, 0.1 mmol, 1.0 equiv), TEMPO (80 mg, 0.5 mmol, 5.0 equiv), and 4DPAIPN (1.6 mg, 0.002 mmol, 0.02 equiv) were added, and the vial was subjected to 3 cycles of vacuum/argon degassing. Subsequently, 0.5 mL of dry DMSO was added under inert atmosphere and the solution was degassed with Argon for 30 seconds. The reaction mixture was irradiated for 1.5 h with a 427 nm Kessil PR160-violet LED as described in the "*Workflow*" section. The temperature of the reaction was maintained at approximately 25 °C via a fan. After the reaction time, the mixture was diluted with AcOEt (10 mL) and washed with brine (3 x 10 mL). The organic layer was dried over Na_2SO_4, filtered, and concentrated under

[4] Tucker, J. W.; Stephenson. C. R. Shining Light on Photoredox Catalysis: Theory and Synthetic Applications. *J. Org. Chem.* **2012**, *77*, 1617-1622.

reduced pressure. The crude mixture was analyzed by ^{1}H- and ^{19}F- NMR observing null formation of compound **4** and ~65% of unreacted styrene **2b**.

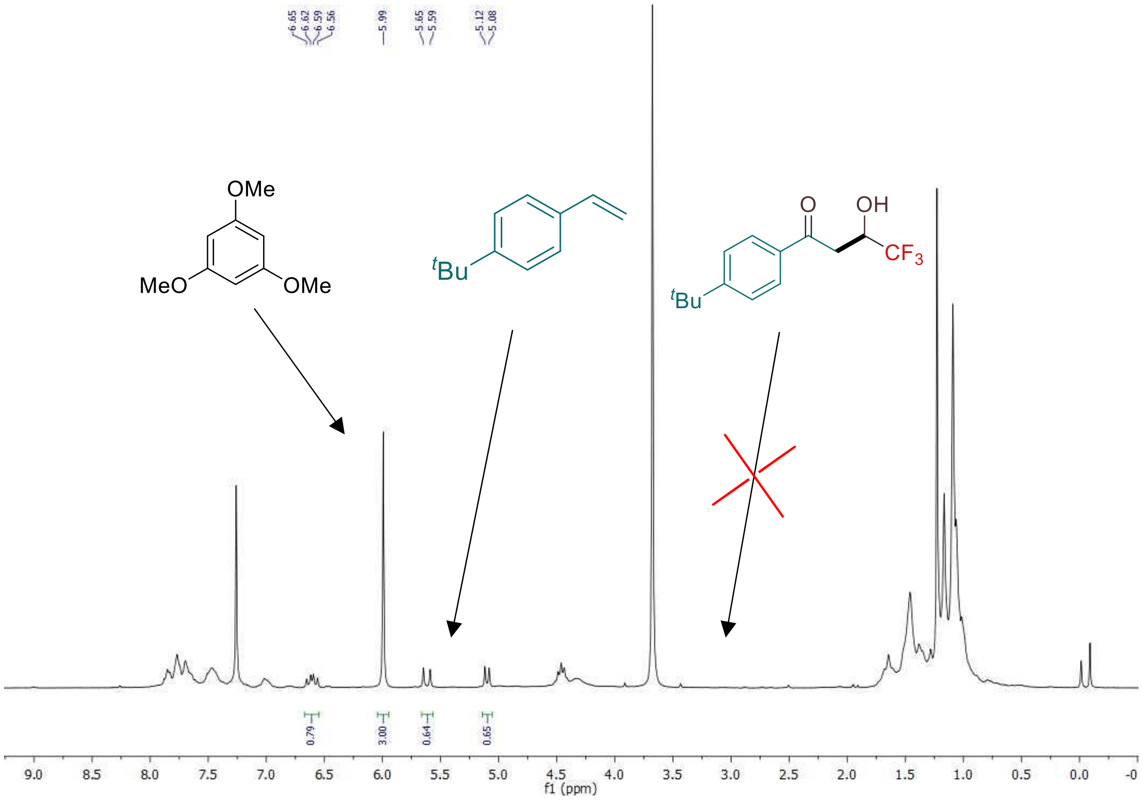

Figure S6: *^{1}H NMR (300 MHz, $CDCl_3$) of the TEMPO experiment reaction using 1,3,5-trimethoxybenzene as internal standard.*

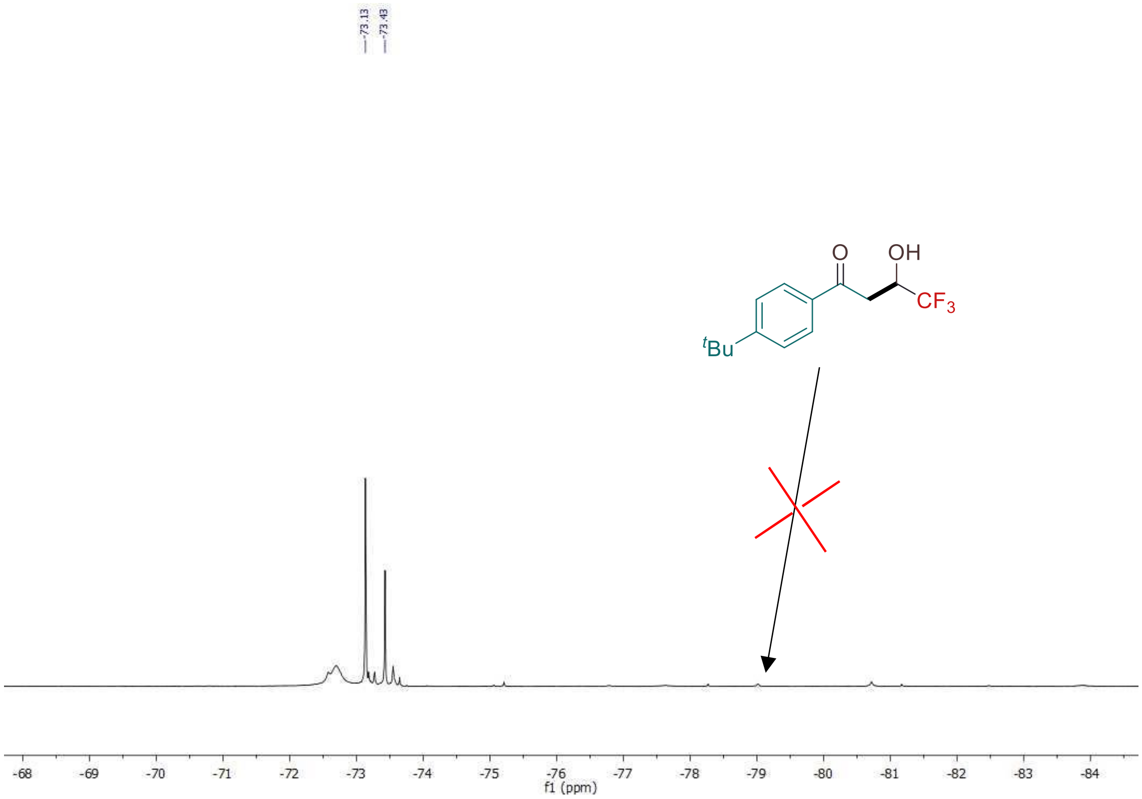

Figure S7: *^{19}F NMR (282 MHz, CDCl$_3$) of the TEMPO experiment reaction.*

__7.3.__ Stern-Volmer Quenching Studies

Fluorescence measurements were obtained using septa-capped UV-Quartz cuvettes (10 mm pathlength) obtained from Hellma Analytics. Excitation was performed at 400 nm; fluorescence spectra were obtained from 300-700 nm. The stock solutions were prepared as follows:

1. Photocatalyst 4DPAIPN solution (0.0000002 M): To a 25 mL volumetric flask was added 3.90 mg of 4DPAIPN and was dissolved in DMSO. Subsequently, 10 µL of the previous solution was added to a 10 mL volumetric flask and was diluted with DMSO obtaining a 1.96x10^{-6} M solution of 4DPAIPN.
2. Redox active ether (RAE) **1a** solution (0.004 M): To a 10 mL volumetric flask was added 10.3 mg of RAE **1a** and was dissolved in DMSO obtaining a 4.20x10^{-3} M solution of RAE derivative **1a**.
3. Styrene solution (0.004 M): To a 10 mL volumetric flask was added 7.6 mg of styrene **2a** and was dissolved in DMSO obtaining a 4.68x10^{-3} M solution of styrene derivative **2a**.

Following preparation, the solutions were allocated to the cuvettes and fluorescence quenching was determined with individual quenchers (phthalimide ether and styrene). 1 mL of the photocatalyst solution was added together with 1 mL of DMSO for the initial measurement. For

the experiments with quencher, 1 mL of photocatalyst solution was added together with increasing amounts of quencher (10, 50, 500 and 1000 μL) and adjusting concentration with volume of DMSO to reach 2 mL. Degassing of each individual solution for 30 seconds was performed prior to recording the data. Linear regression of I_0/I against concentration was carried out to yield the Stern-Volmer quenching rate constant (K_{SV}). The following Stern-Volmer plots for luminescence quenching of 4DPAIPN (1.96×10^{-6} M in degassed DMSO) by quenchers were obtained. The excited catalyst is only quenched by the *redox active ether* **1a** with a Stern-Volmer quenching rate constant of 85.7 M^{-1}.

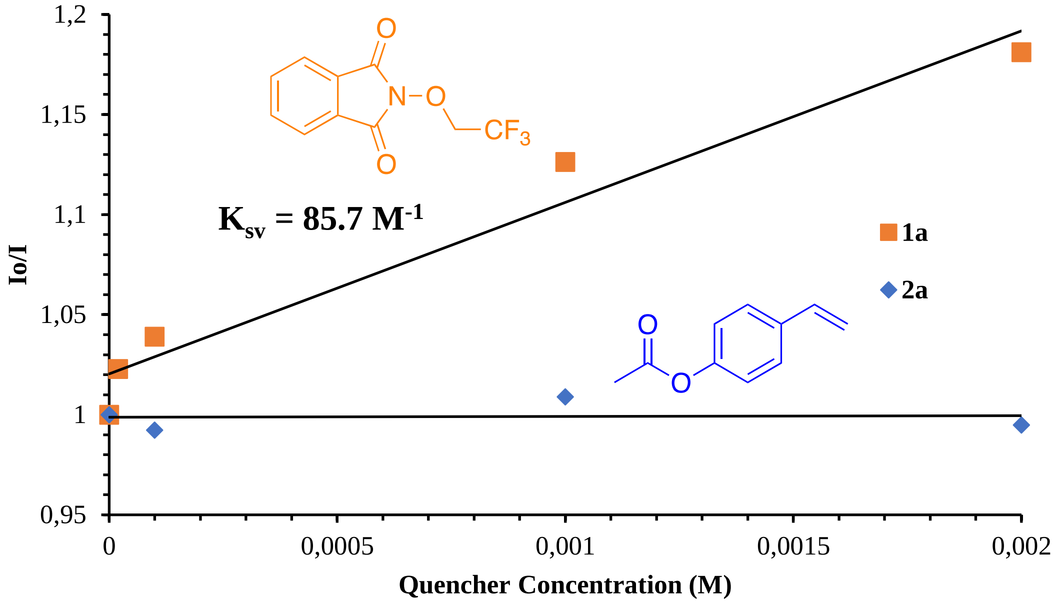

Figure S8. *Stern-Volmer plots for luminescence quenching of 4DPAIPN (1.96 x 10^{-6} M in degassed DMSO) by redox active ether* ***1a*** *(orange) and styrene* ***2a*** *(blue),* $\lambda_{exc.}$ *= 400 nm,* $\lambda_{em.}$ *= 530 nm,* K_{sv} *= Stern-Volmer constant.*

6. Data of X-Ray Structure of Compound 11

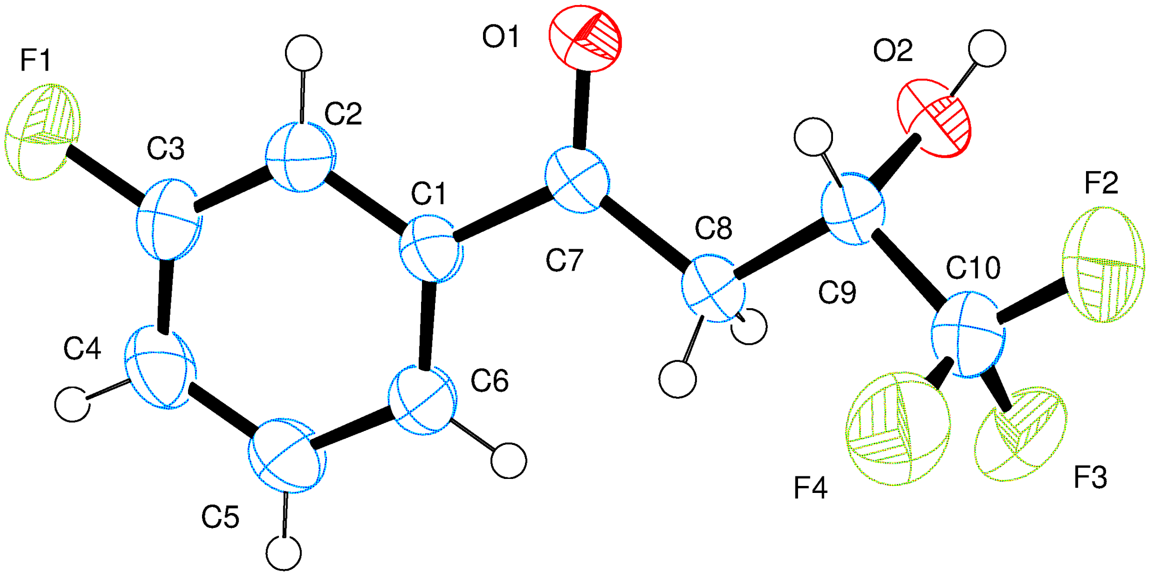

***Figure S9**. Ortep view of **11** showing the atom labelling and the thermal vibration ellipsoids at 50% probability*

Adequate crystals were obtained by crystallization from DCM for compound **11**. A clear colorless, needle-like specimen of $C_{10}H_8F_4O_2$, approximate dimensions 0.110 mm x 0.130 mm x 0.460 mm, was used for the X-ray crystallographic analysis. The X-ray intensity data were measured (λ = 0.71073 Å).

The total exposure time was 14.20 hours. The frames were integrated with the Bruker SAINT software package using a narrow-frame algorithm. The integration of the data using a monoclinic unit cell yielded a total of 29647 reflections to a maximum θ angle of 28.56° (0.74 Å resolution), of which 2573 were independent. The final cell constants of a = 4.8540(11) Å, b = 10.187(2) Å, c = 20.402(5) Å, β = 93.178(5)°, volume = 1007.3(4) Å3, are based upon the refinement of the XYZ-centroids of 6539 reflections above 20 σ(I) with 4.462° < 2θ < 49.23°. Data were corrected for absorption effects using the Multi-Scan method (SADABS). The ratio of minimum to maximum apparent transmission was 0.950. The calculated minimum and maximum transmission coefficients (based on crystal size) are 0.9320 and 0.9830.

The structure was solved and refined using the Bruker SHELXTL Software Package, using the space group P 21/n, with Z = 4 for the formula unit, $C_{10}H_8F_4O_2$. The final anisotropic full-matrix least-squares refinement on F2 with 148 variables converged at R1 = 4.24%, for the observed data and wR2 = 11.69% for all data. The goodness-of-fit was 1.039. The largest peak in the final difference electron density synthesis was 0.232 e-/Å3 and the largest hole was -0.191 e-/Å3 with an RMS deviation of 0.041 e-/Å3. The seven highest residuals are located at the center of C-C bonds. On the basis of the final model, the calculated density was 1.557 g/cm^3.

Table S2. *Crystal data and structure refinement for Compound* ***11****.*

Identification code	**11**	
Empirical formula	$C_{10}H_8F_0O_2$	
Formula weight	236.16	
Temperature	298(2) K	
Wavelength	0.71073 Å	
Crystal system	Monoclinic	
Space group	P 21/n	
Unit cell dimensions	a = 4.8540(11) Å	α= 90°.
	b = 10.187(2) Å	β= 93.178(5)°.
	c = 20.402(5) Å	γ = 90°.
Volume	1007.3(4) $Å^3$	
Z	4	
Density (calculated)	1.557 Mg/m^3	
Absorption coefficient	0.155 mm^{-1}	
F(000)	480	
Crystal size	0.460 x 0.130 x 0.110 mm^3	
Theta range for data collection	1.999 to 28.557°.	
Index ranges	-6<=h<=6, -13<=k<=13, -27<=l<=27	
Reflections collected	29647	
Independent reflections	2573 [R(int) = 0.0341]	
Refinement method	Full-matrix least-squares on F^2	
Data / restraints / parameters	2573 / 0 / 148	
Goodness-of-fit on F^2	1.039	
Final R indices [I>2sigma(I)]	R1 = 0.0424, wR2 = 0.1078	
R indices (all data)	R1 = 0.0534, wR2 = 0.1169	
Extinction coefficient	0.022(3)	
Largest diff. peak and hole	0.232 and -0.191 e.$Å^{-3}$	

Table S3. *Atomic coordinates (x 10^4) and equivalent isotropic displacement parameters (Å^{2}x 10^3) for Compound **11**. U(eq) is defined as one third of the trace of the orthogonalized U^{ij} tensor.*

	x	y	z	U(eq)
F(1)	11444(2)	4137(1)	7524(1)	85(1)
F(2)	-529(3)	2689(1)	3715(1)	86(1)
F(3)	-519(3)	1074(1)	4383(1)	85(1)
F(4)	3255(3)	1701(1)	3984(1)	90(1)
O(1)	3892(2)	4556(1)	5794(1)	47(1)
O(2)	-1134(2)	3551(1)	4978(1)	47(1)
C(1)	6294(3)	2853(1)	6354(1)	36(1)
C(2)	7925(3)	3745(1)	6715(1)	45(1)
C(3)	9824(3)	3271(2)	7179(1)	52(1)
C(4)	10186(3)	1957(2)	7305(1)	55(1)
C(5)	8555(3)	1079(2)	6949(1)	54(1)
C(6)	6610(3)	1519(1)	6474(1)	45(1)
C(7)	4317(2)	3382(1)	5832(1)	36(1)
C(8)	2956(3)	2421(1)	5353(1)	40(1)
C(9)	1410(3)	3086(1)	4777(1)	38(1)
C(10)	903(3)	2130(2)	4216(1)	55(1)

Table S4. *Bond lengths [Å] and angles [°] for Compound **11**.*

F(1)-C(3)	1.3531(17)
F(2)-C(10)	1.3325(18)
F(3)-C(10)	1.333(2)
F(4)-C(10)	1.333(2)
O(1)-C(7)	1.2158(16)
O(2)-C(9)	1.4048(16)
C(1)-C(6)	1.3878(19)
C(1)-C(2)	1.3891(19)
C(1)-C(7)	1.4936(17)
C(2)-C(3)	1.372(2)
C(3)-C(4)	1.372(2)
C(4)-C(5)	1.374(2)
C(5)-C(6)	1.3891(19)

C(7)-C(8)	1.5096(18)
C(8)-C(9)	1.5187(17)
C(9)-C(10)	1.512(2)
C(6)-C(1)-C(2)	119.49(12)
C(6)-C(1)-C(7)	122.63(12)
C(2)-C(1)-C(7)	117.85(12)
C(3)-C(2)-C(1)	118.51(13)
F(1)-C(3)-C(2)	118.56(14)
F(1)-C(3)-C(4)	118.30(13)
C(2)-C(3)-C(4)	123.13(14)
C(3)-C(4)-C(5)	118.13(13)
C(4)-C(5)-C(6)	120.53(14)
C(1)-C(6)-C(5)	120.21(14)
O(1)-C(7)-C(1)	120.02(12)
O(1)-C(7)-C(8)	122.03(11)
C(1)-C(7)-C(8)	117.93(11)
C(7)-C(8)-C(9)	113.06(11)
O(2)-C(9)-C(10)	109.20(11)
O(2)-C(9)-C(8)	109.21(11)
C(10)-C(9)-C(8)	110.55(12)
F(2)-C(10)-F(3)	106.59(13)
F(2)-C(10)-F(4)	106.73(14)
F(3)-C(10)-F(4)	107.01(14)
F(2)-C(10)-C(9)	111.18(13)
F(3)-C(10)-C(9)	113.06(14)
F(4)-C(10)-C(9)	111.90(13)

Table S5. *Anisotropic displacement parameters ($Å^2$x 10^3)for Compound **11**. The anisotropic displacement factor exponent takes the form: $-2\pi^2[h^2a^{*2}U^{11} + ... + 2 h k a^* b^* U^{12}]$*

	U^{11}	U^{22}	U^{33}	U^{23}	U^{13}	U^{12}
F(1)	87(1)	72(1)	90(1)	-16(1)	-50(1)	-6(1)
F(2)	105(1)	96(1)	54(1)	-9(1)	-39(1)	12(1)
F(3)	95(1)	55(1)	100(1)	-14(1)	-28(1)	-17(1)
F(4)	79(1)	111(1)	78(1)	-43(1)	-2(1)	21(1)
O(1)	52(1)	35(1)	52(1)	1(1)	-13(1)	7(1)
O(2)	39(1)	49(1)	51(1)	9(1)	-4(1)	10(1)
C(1)	37(1)	38(1)	34(1)	1(1)	-4(1)	3(1)
C(2)	49(1)	39(1)	45(1)	-2(1)	-9(1)	2(1)
C(3)	50(1)	56(1)	48(1)	-8(1)	-16(1)	0(1)
C(4)	56(1)	62(1)	46(1)	3(1)	-18(1)	12(1)
C(5)	64(1)	44(1)	53(1)	9(1)	-15(1)	9(1)
C(6)	50(1)	38(1)	46(1)	3(1)	-12(1)	2(1)
C(7)	35(1)	36(1)	37(1)	2(1)	-3(1)	4(1)
C(8)	39(1)	35(1)	46(1)	1(1)	-10(1)	4(1)
C(9)	36(1)	38(1)	40(1)	2(1)	-7(1)	1(1)
C(10)	56(1)	56(1)	52(1)	-8(1)	-15(1)	6(1)

Table S6. *Hydrogen bonds for Compound **11** [Å and °].*

D-H...A	d(D-H)	d(H...A)	d(D...A)	<(DHA)
O(2)-H(2A)...O(1)#1	0.82	1.98	2.7852(14)	166.7
C(9)-H(9)...O(1)#2	0.98	2.59	3.5528(18)	169.0

Symmetry transformations used to generate equivalent atoms:

#1 -x,-y+1,-z+1 #2 -x+1,-y+1,-z+1

7. NMR Spectra

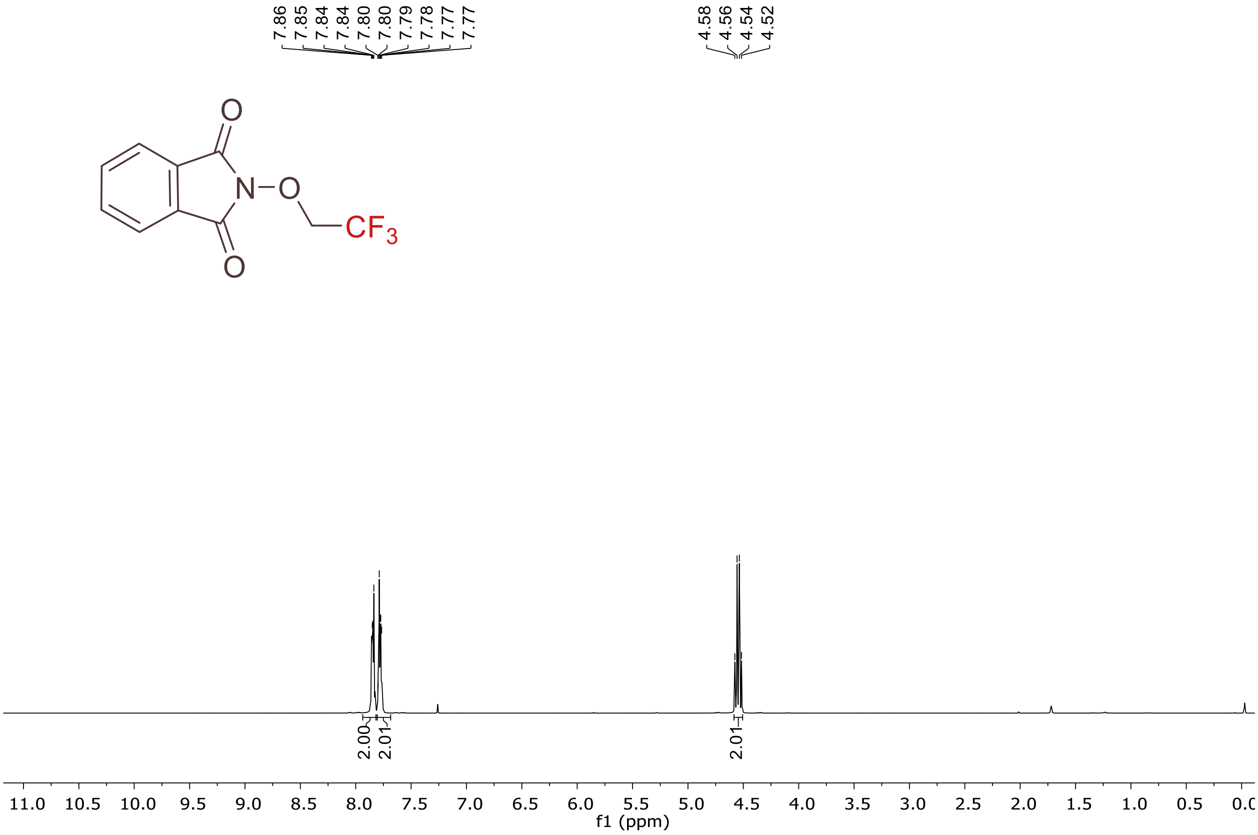

^{1}H NMR (500 MHz, $CDCl_3$) of compound **1a**.

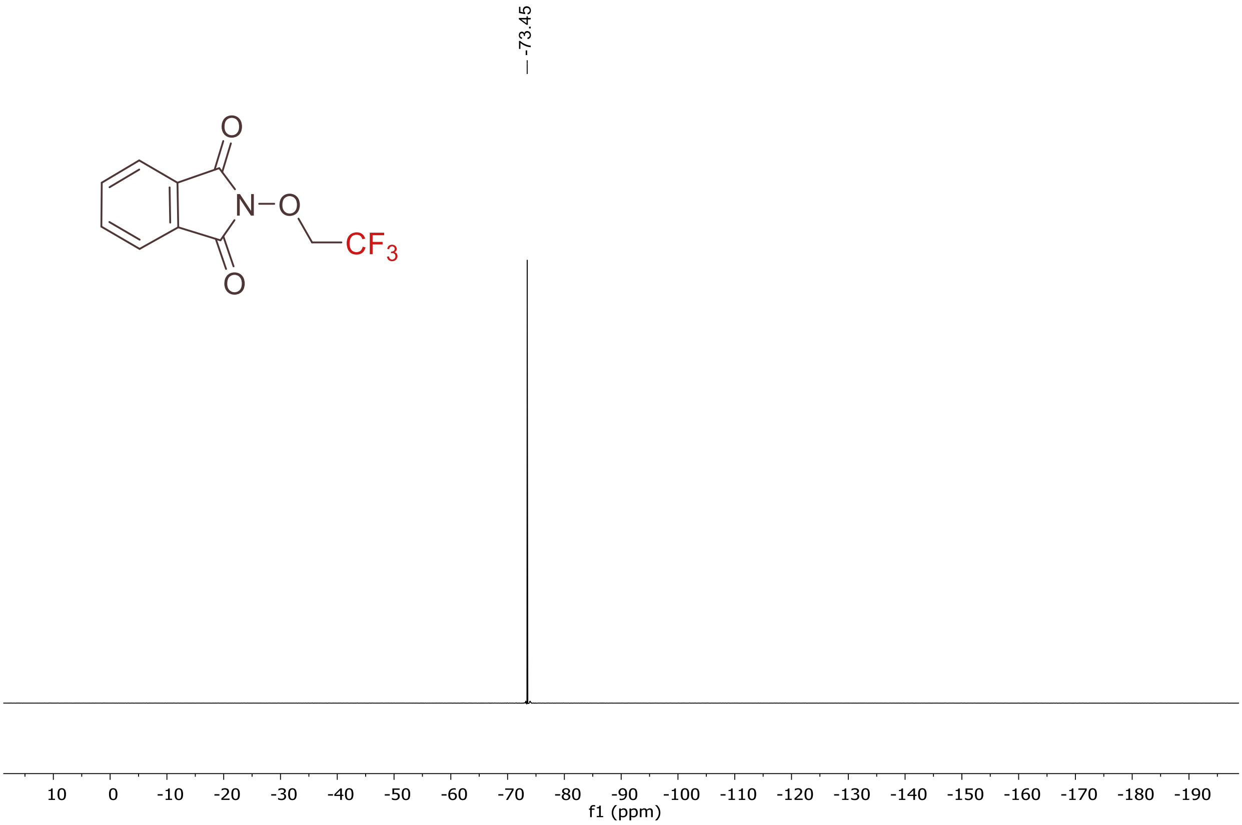

$^{19}F\{^{1}H\}$ NMR (377 MHz, $CDCl_3$) of compound **1a**.

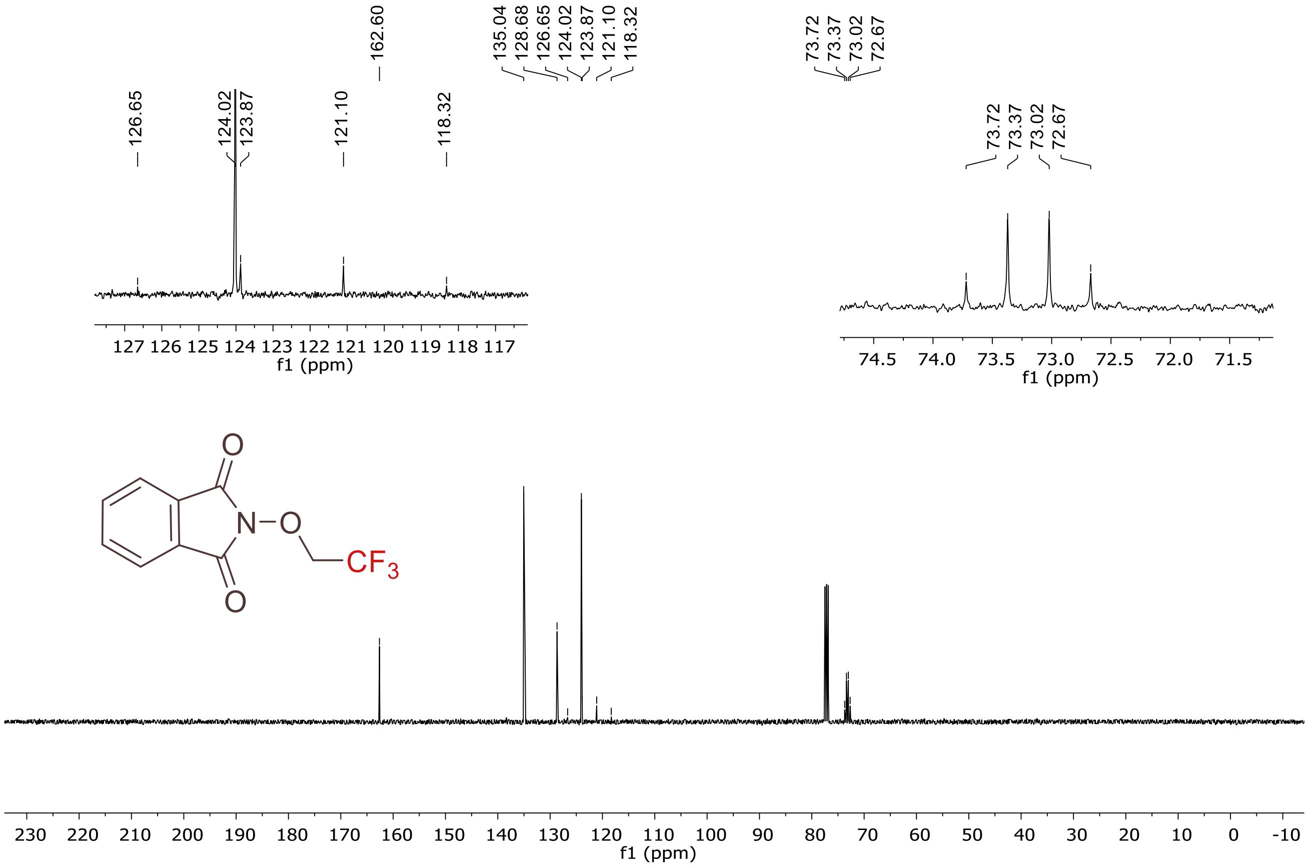

$^{13}C\{^{1}H\}$ NMR (101 MHz, $CDCl_3$) of compound **1a**.

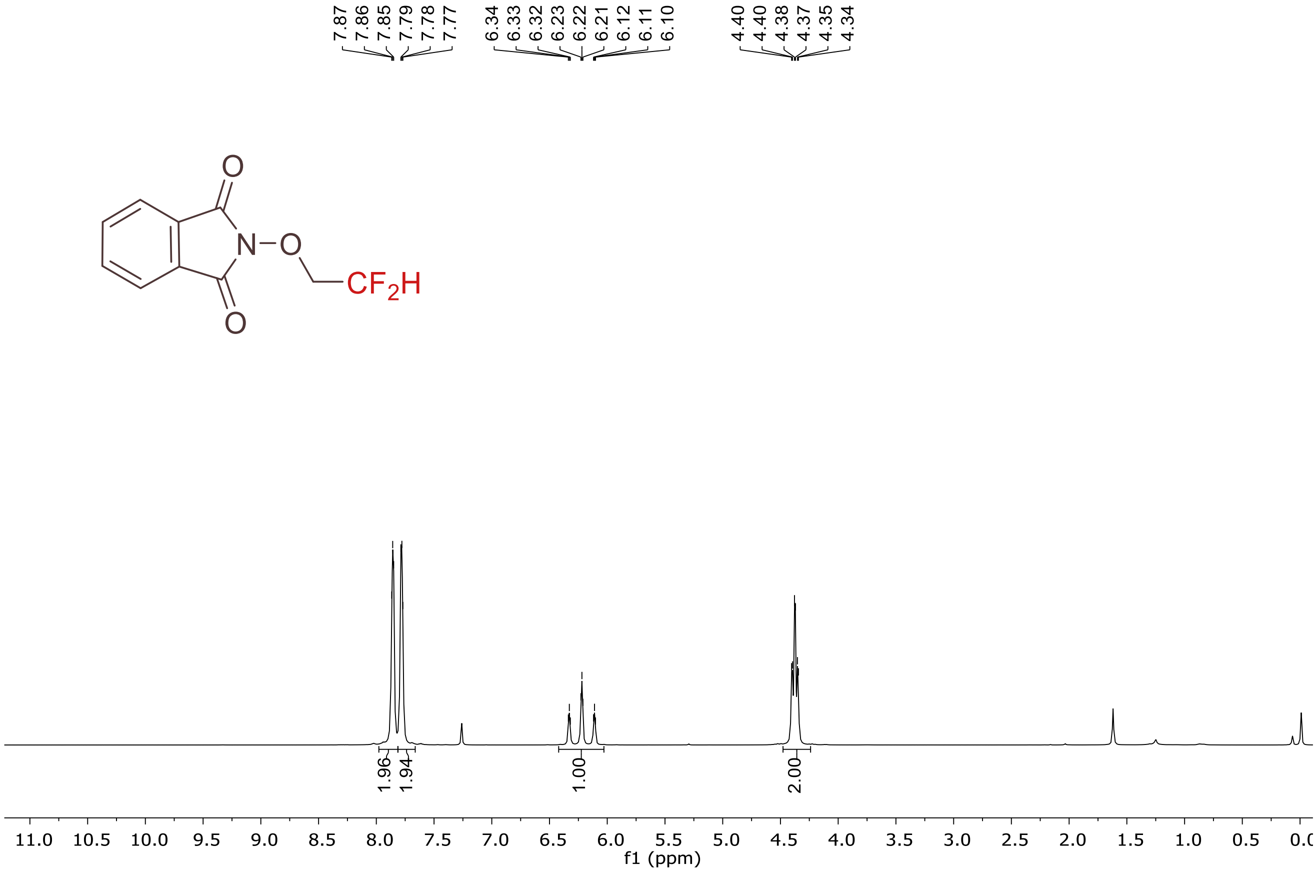

^{1}H NMR (500 MHz, $CDCl_3$) of compound **1b**.

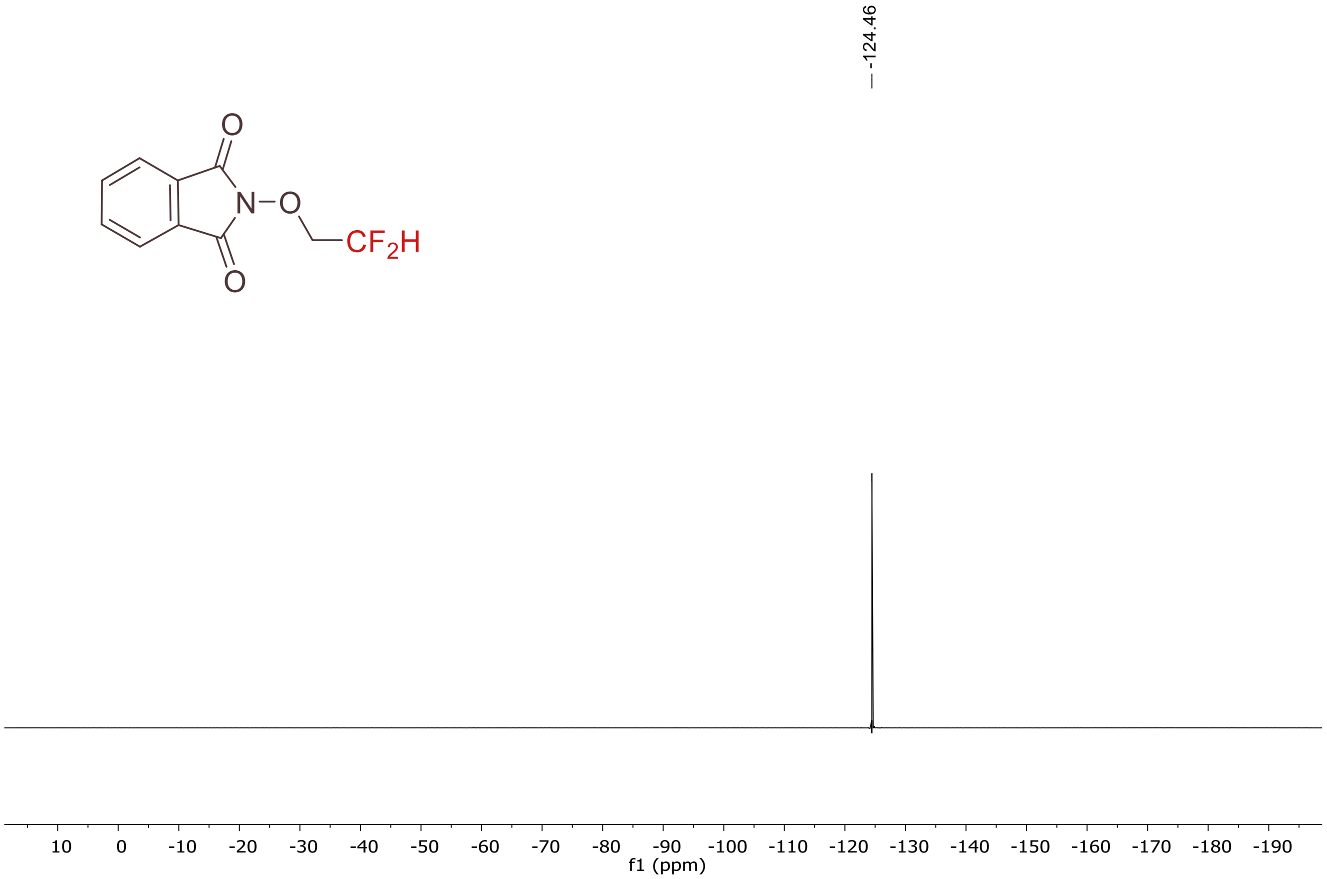

$^{19}F\{^{1}H\}$ NMR (377 MHz, $CDCl_3$) of compound **1b**.

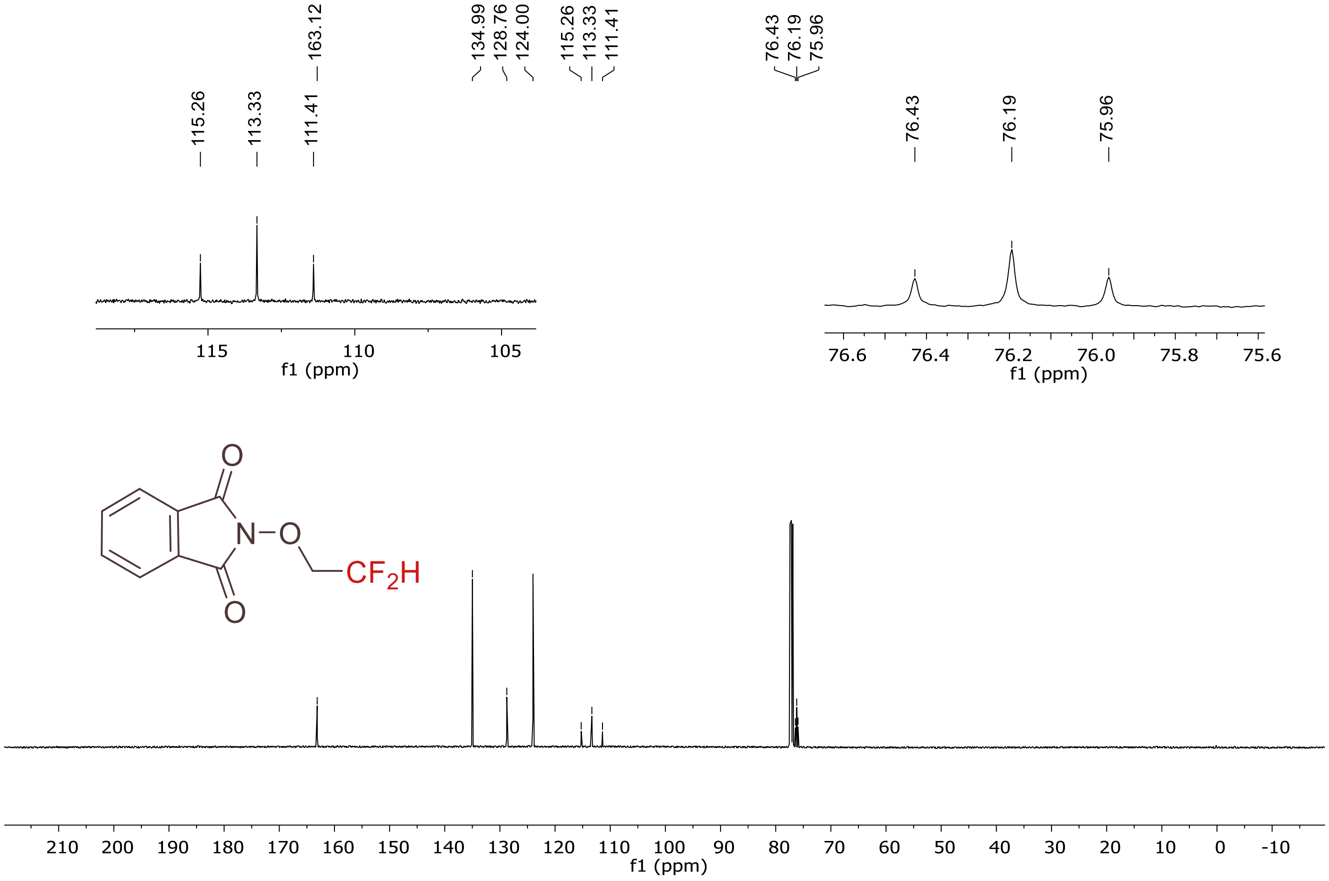

$^{13}C\{^{1}H\}$ NMR (126 MHz, $CDCl_3$) of compound **1b**.

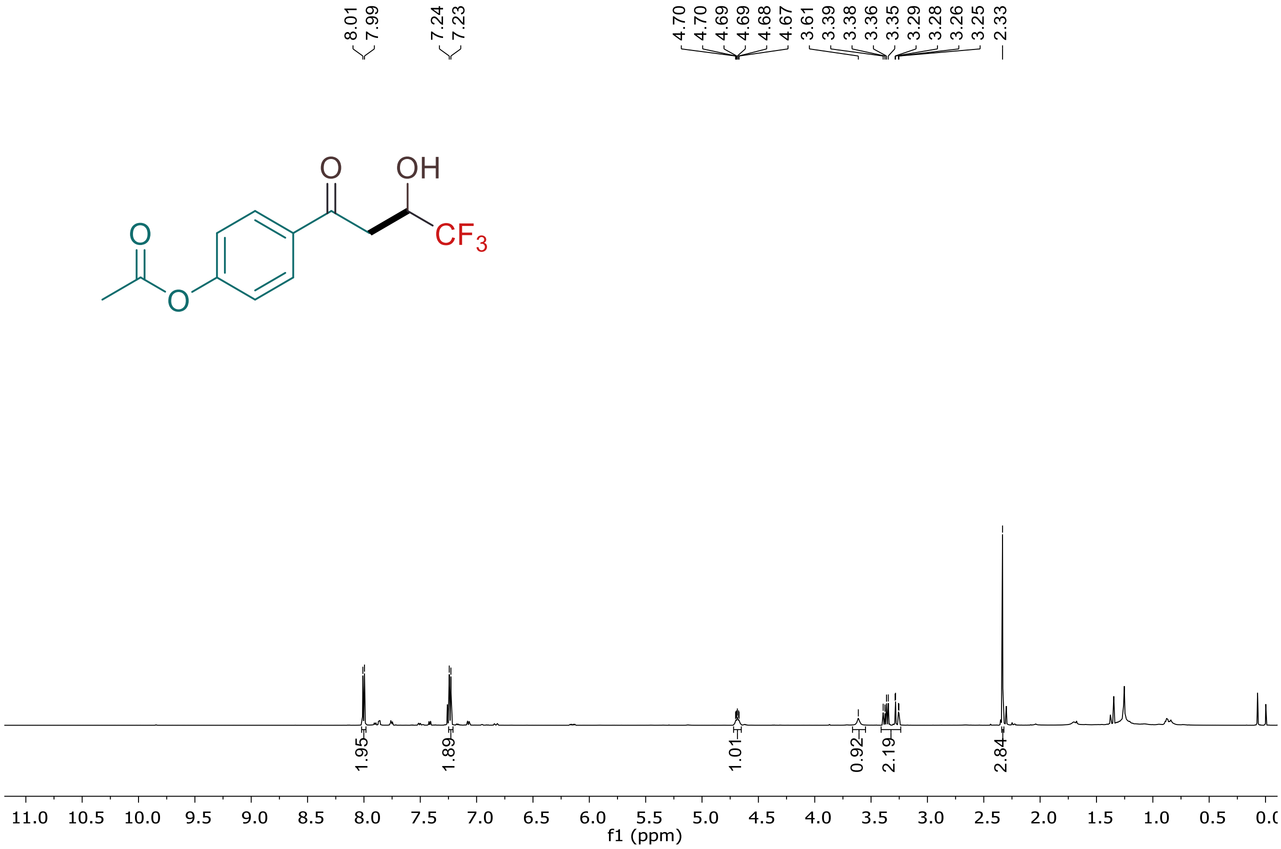

^{1}H NMR (600 MHz, $CDCl_3$) of compound **3**.

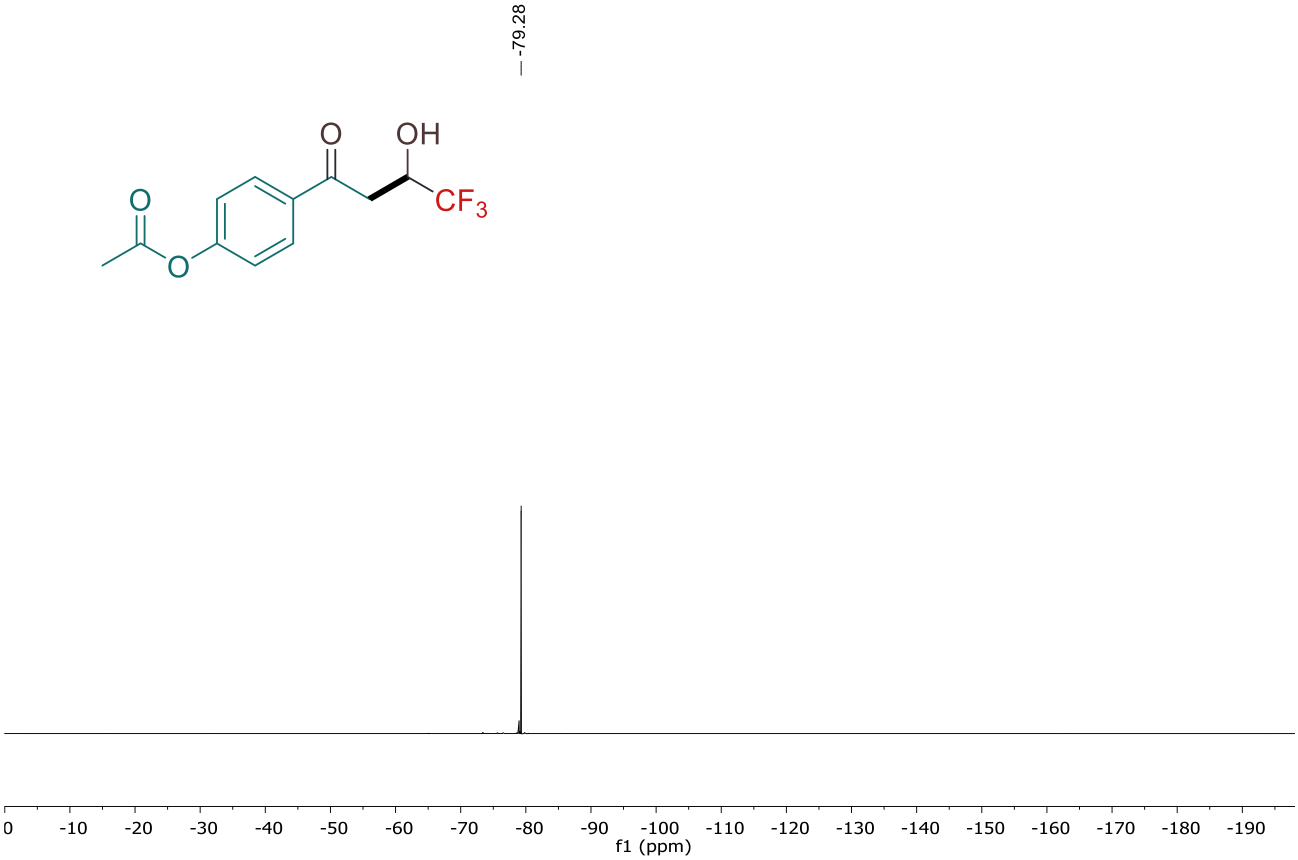

^{19}F{^{1}H} NMR (377 MHz, $CDCl_3$) of compound **3**.

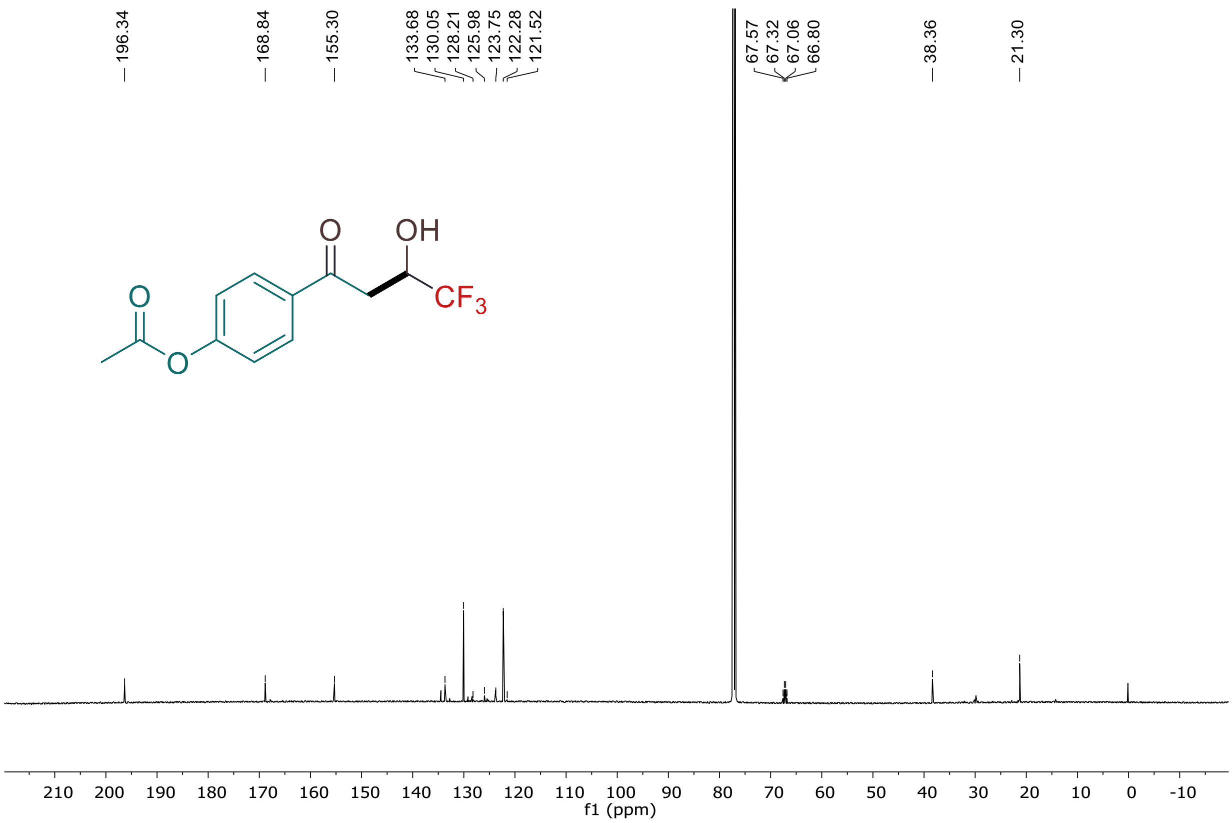

$^{13}C\{^{1}H\}$ NMR (126 MHz, $CDCl_3$) of compound **3**.

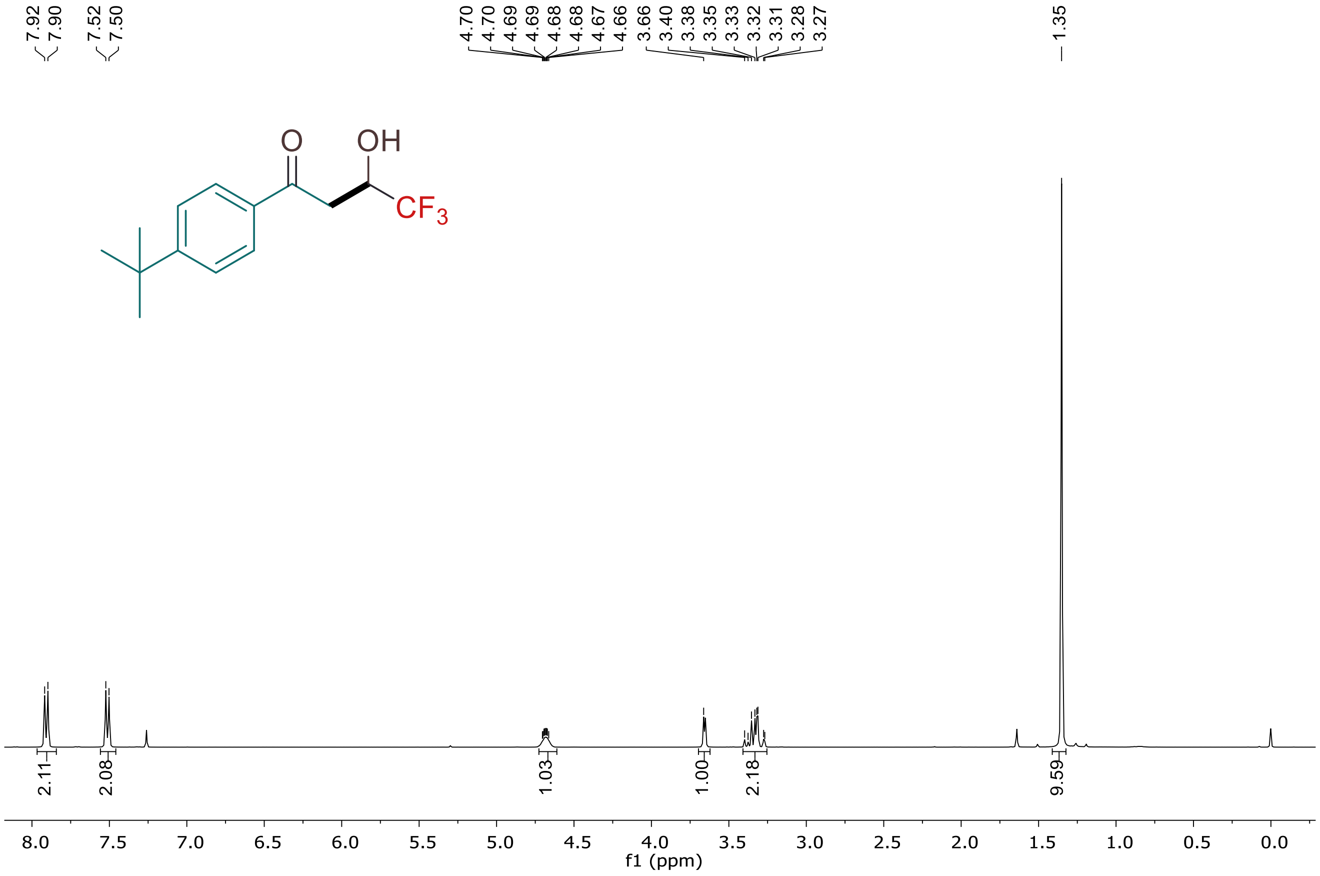

^{1}H NMR (400 MHz, $CDCl_3$) of compound **4**.

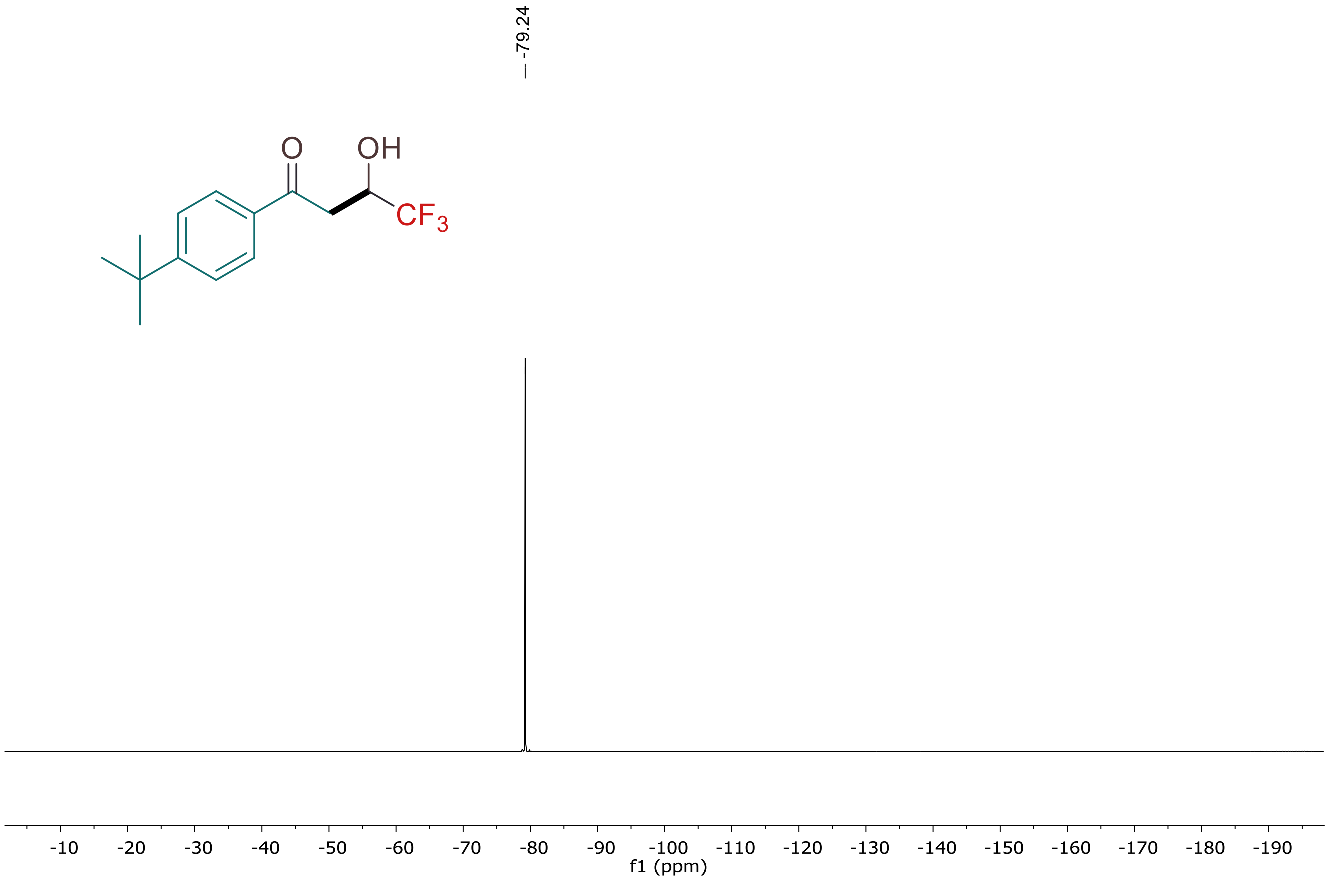

$^{19}F\{^{1}H\}$ NMR (377 MHz, $CDCl_3$) of compound **4**.

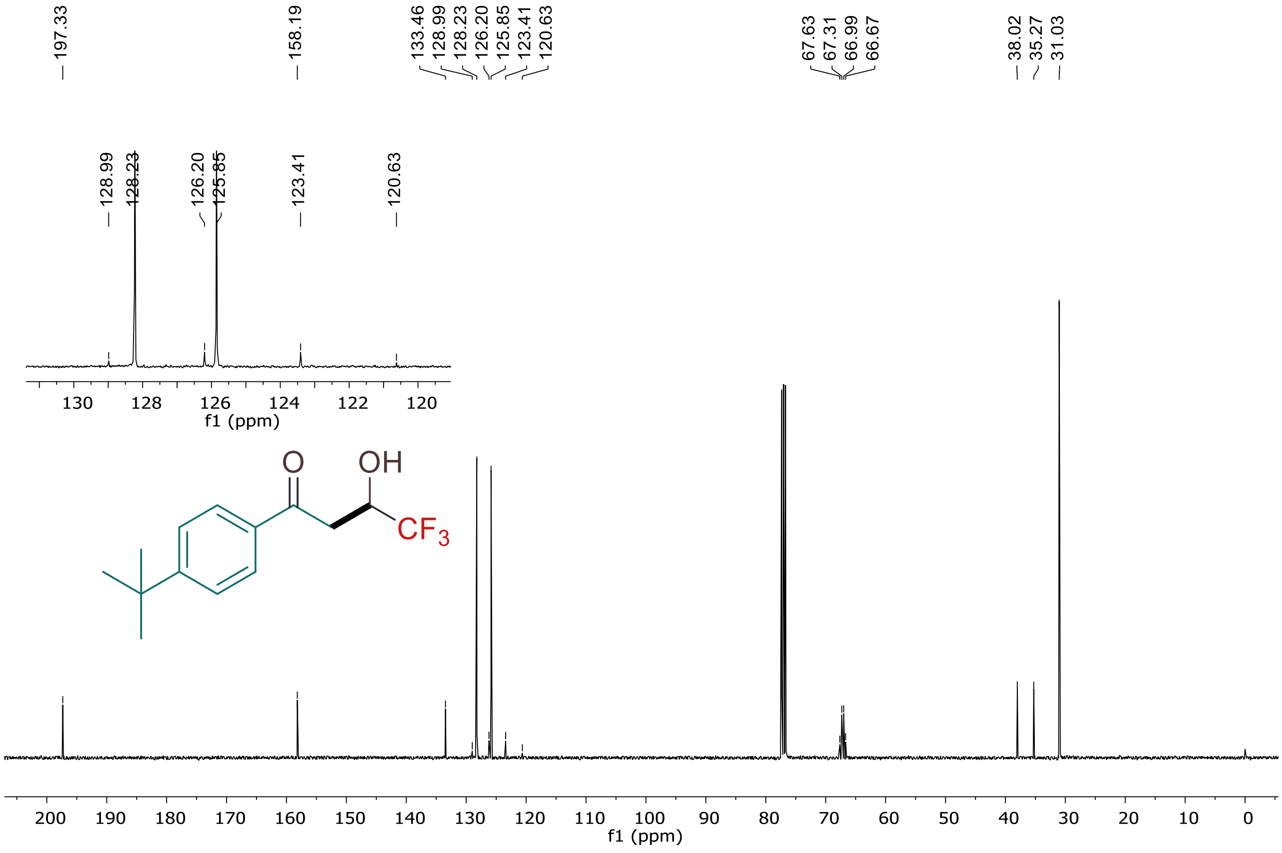

$^{13}C\{^{1}H\}$ NMR (101 MHz, $CDCl_3$) of compound **4**.

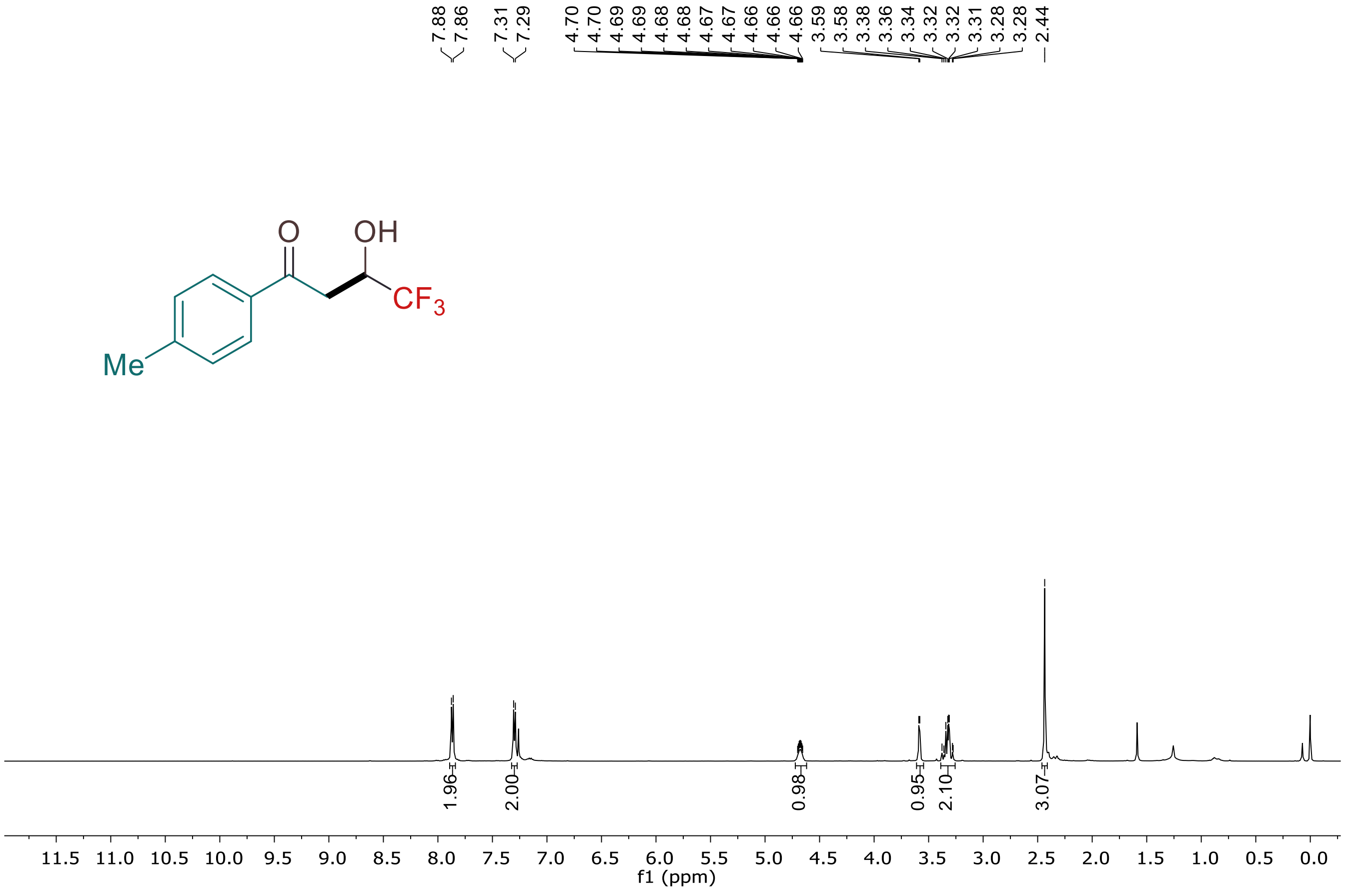

^{1}H NMR (500 MHz, $CDCl_3$) of compound **5**.

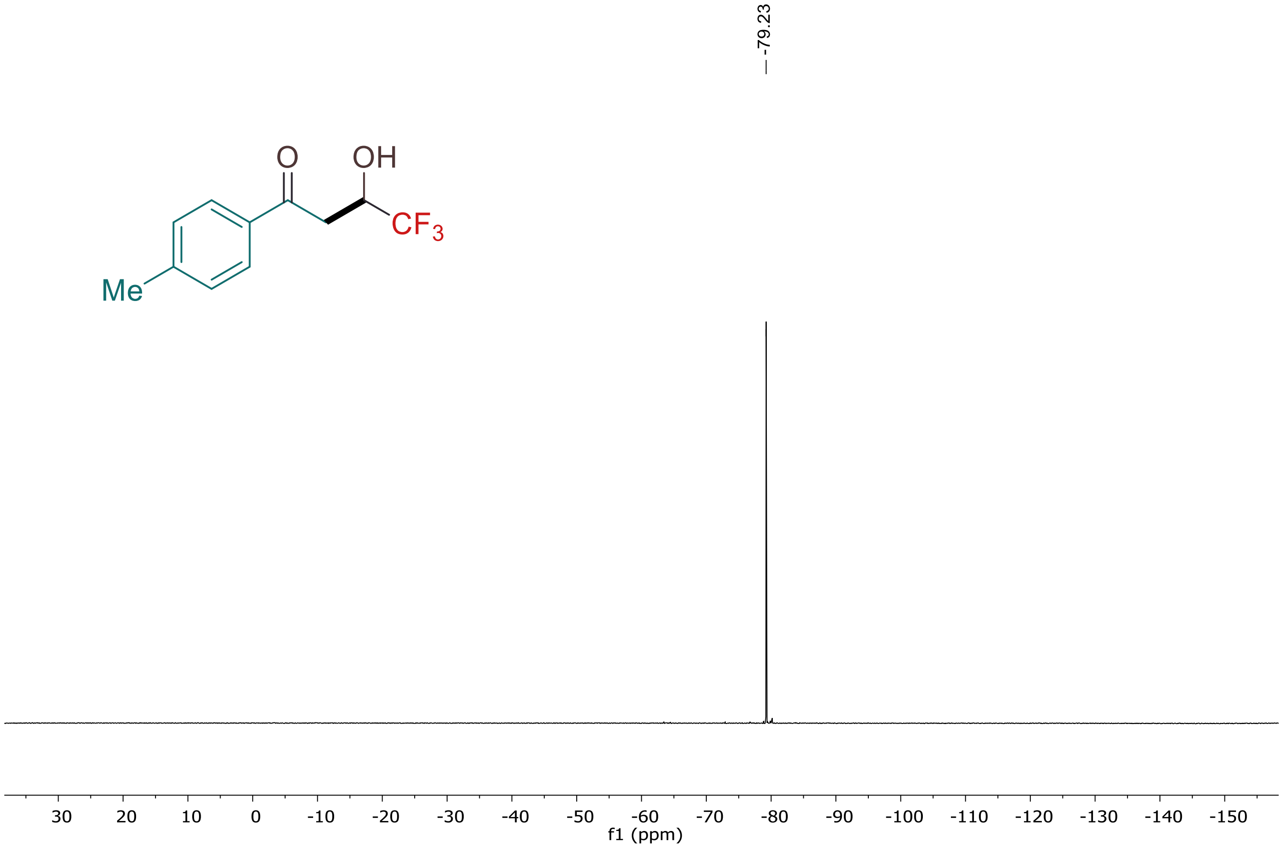

^{19}F{^{1}H} NMR (282 MHz, $CDCl_3$) of compound **5**.

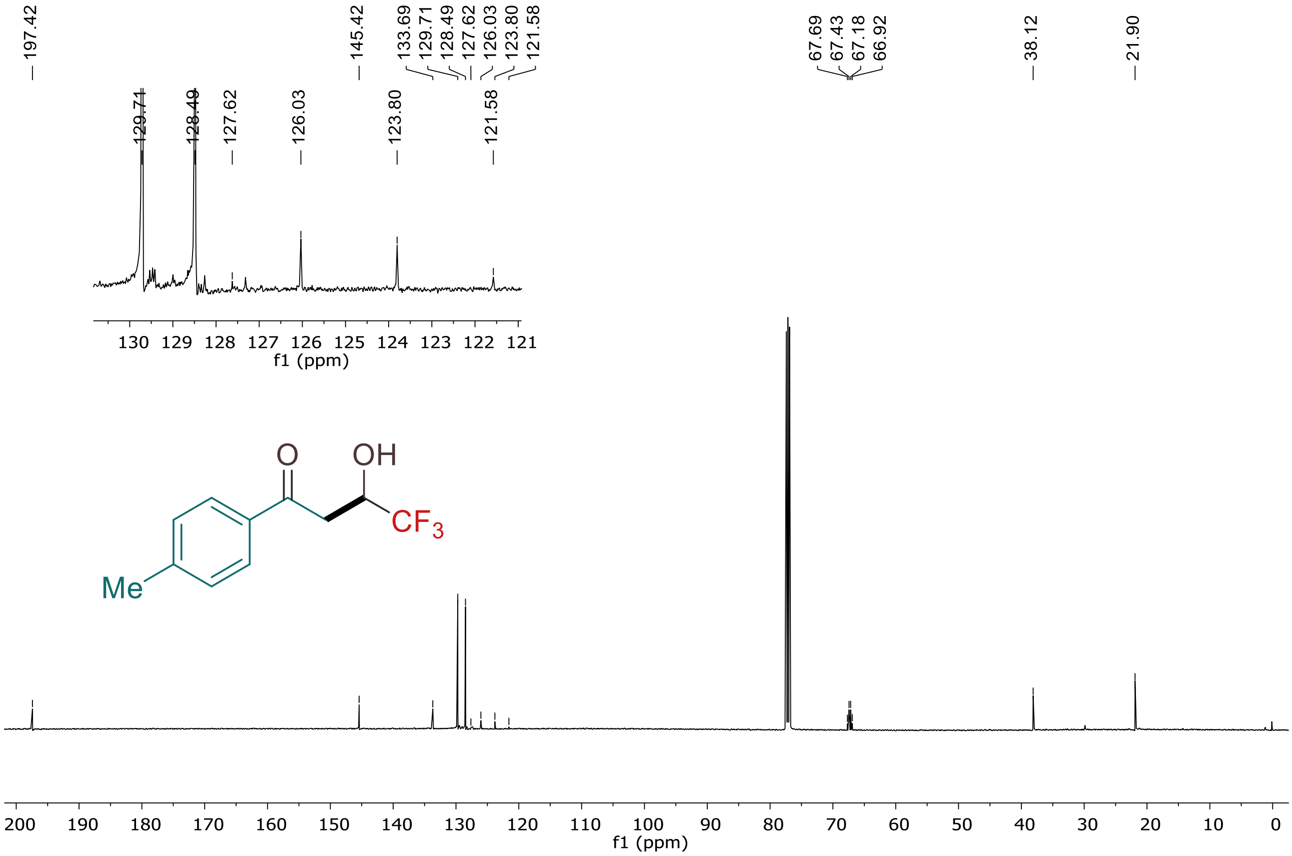

$^{13}C\{^{1}H\}$ NMR (126 MHz, $CDCl_3$) of compound **5**.

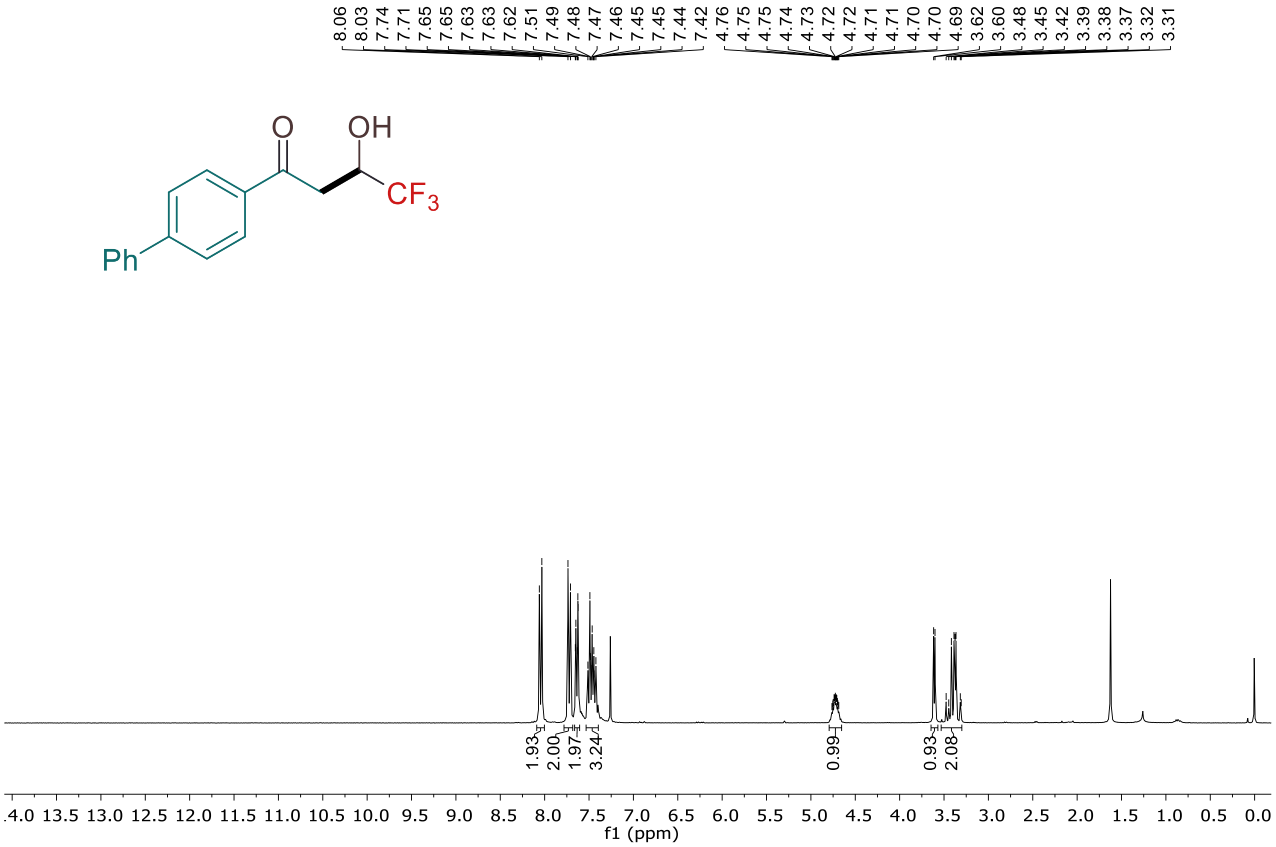

^{1}H NMR (300 MHz, $CDCl_3$) of compound **6**.

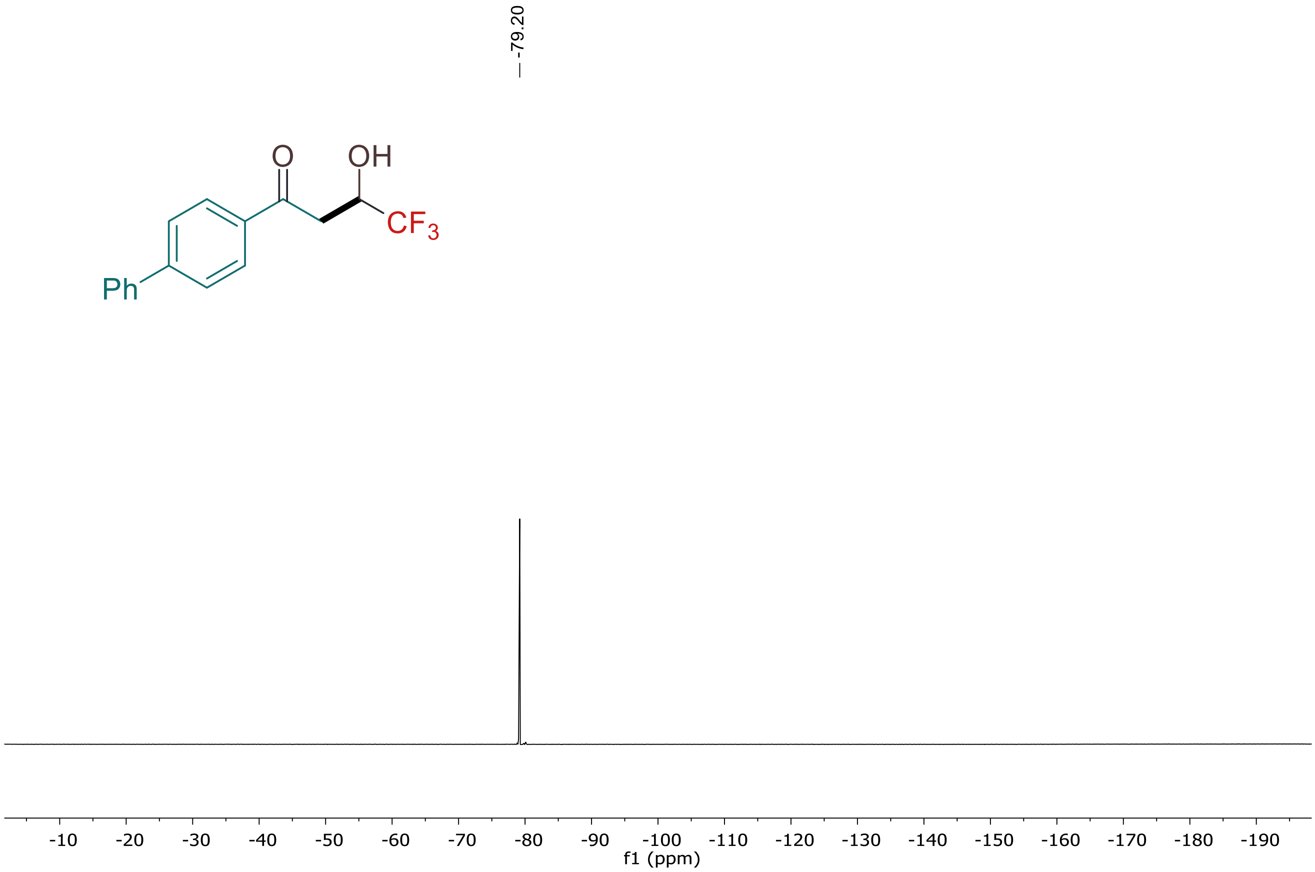

$^{19}F\{^{1}H\}$ NMR (282 MHz, $CDCl_3$) of compound **6**.

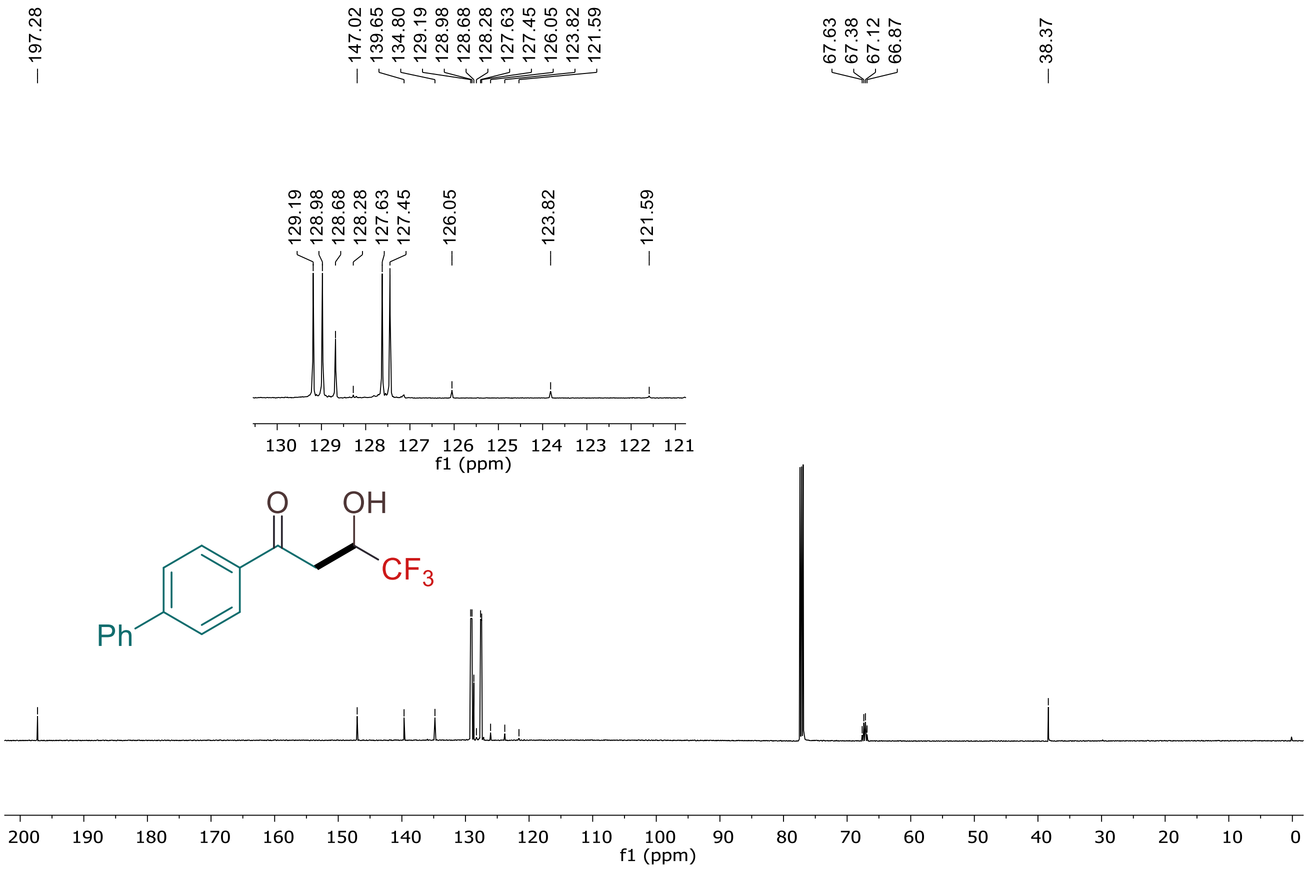

$^{13}C\{^{1}H\}$ NMR (126 MHz, $CDCl_3$) of compound **6**.

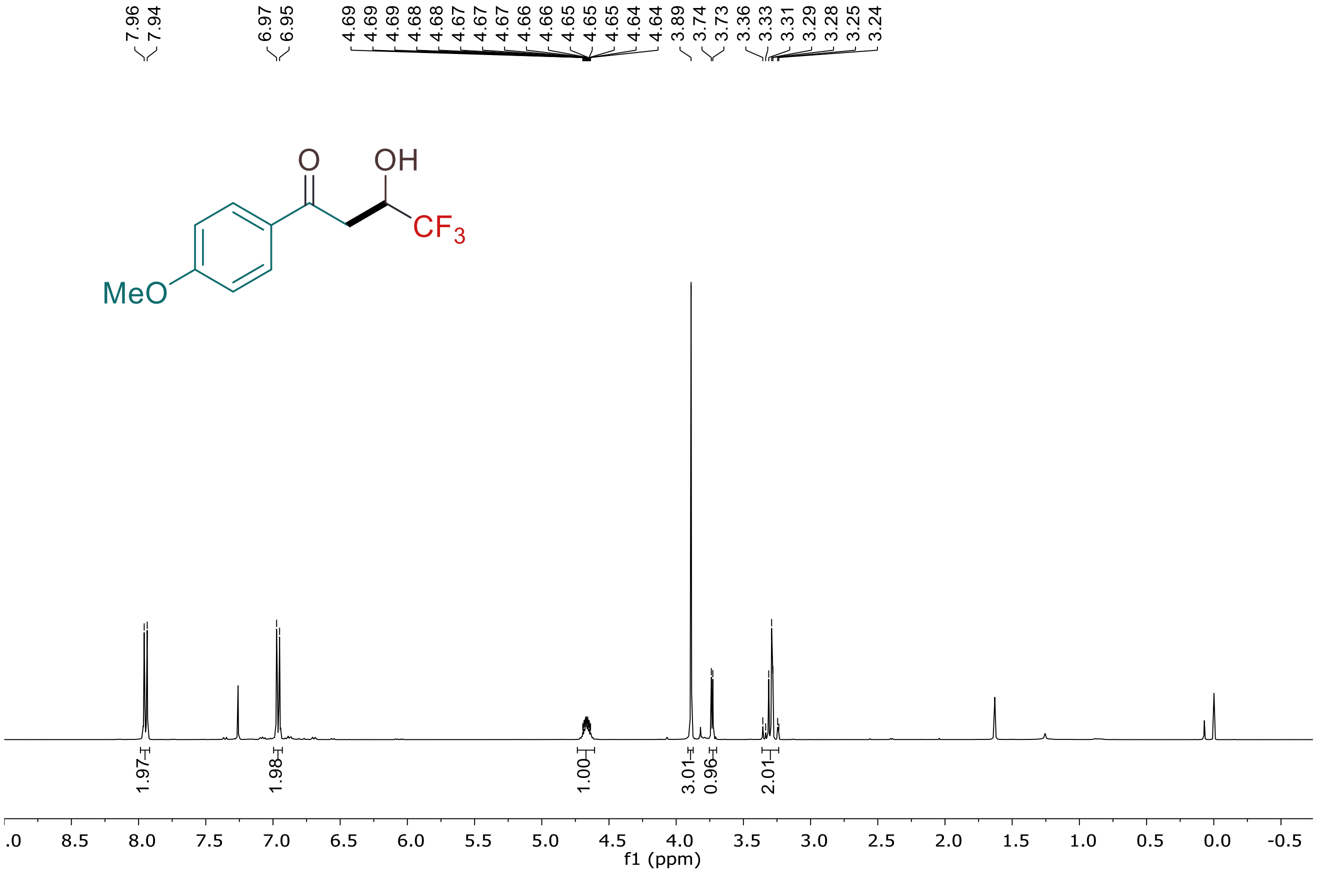

^{1}H NMR (400 MHz, $CDCl_3$) of compound **7**.

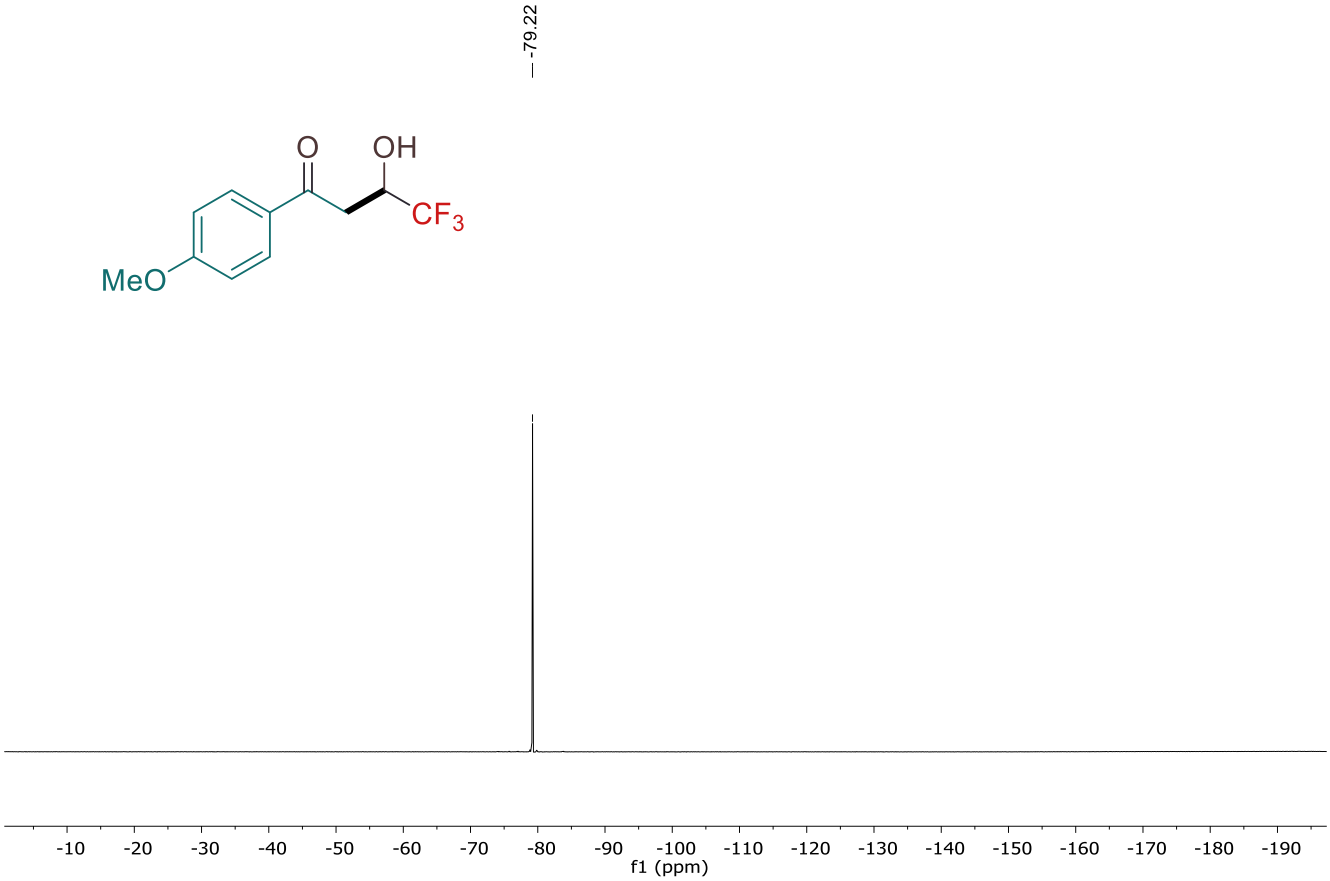

$^{19}F\{^{1}H\}$ NMR (377 MHz, $CDCl_3$) of compound **7**.

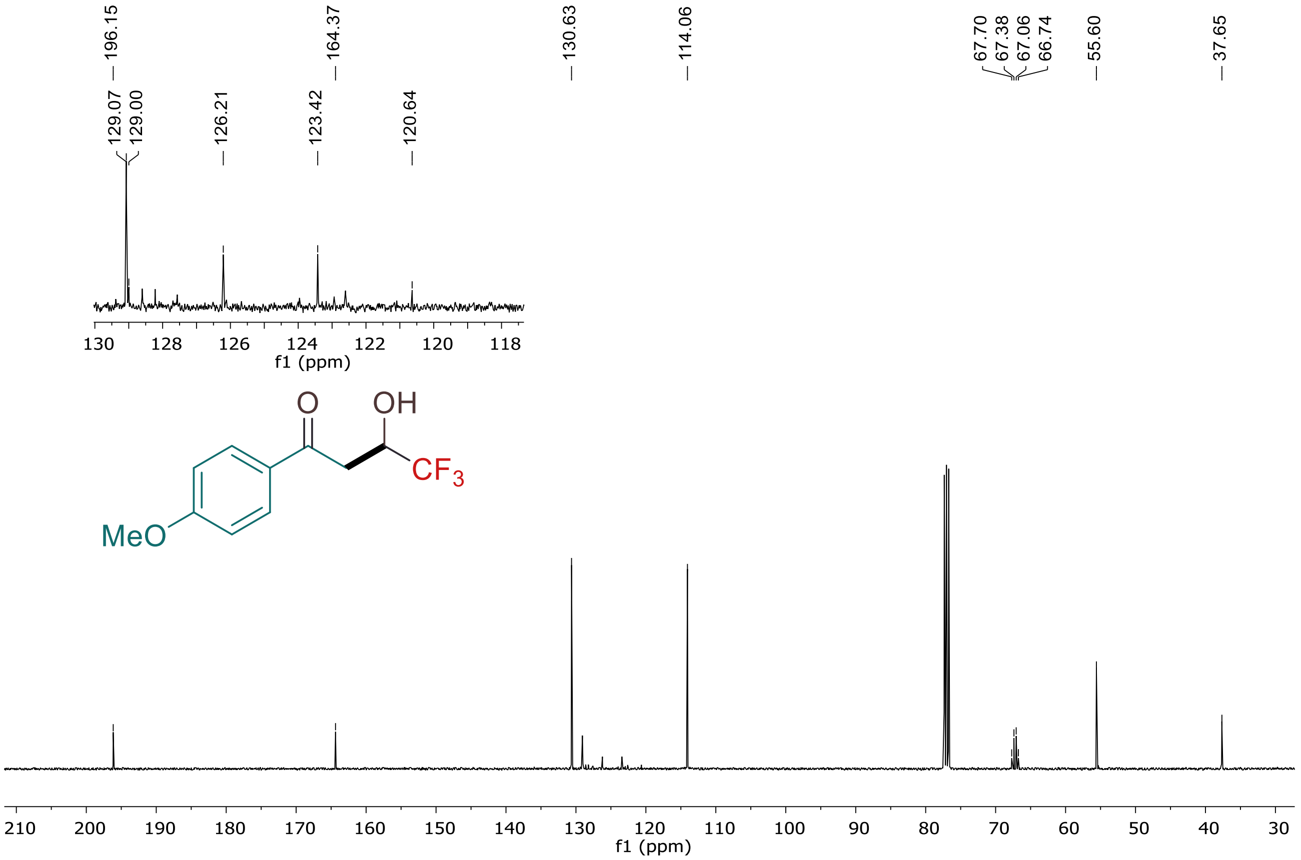

$^{13}C\{^{1}H\}$ NMR (101 MHz, $CDCl_3$) of compound **7**.

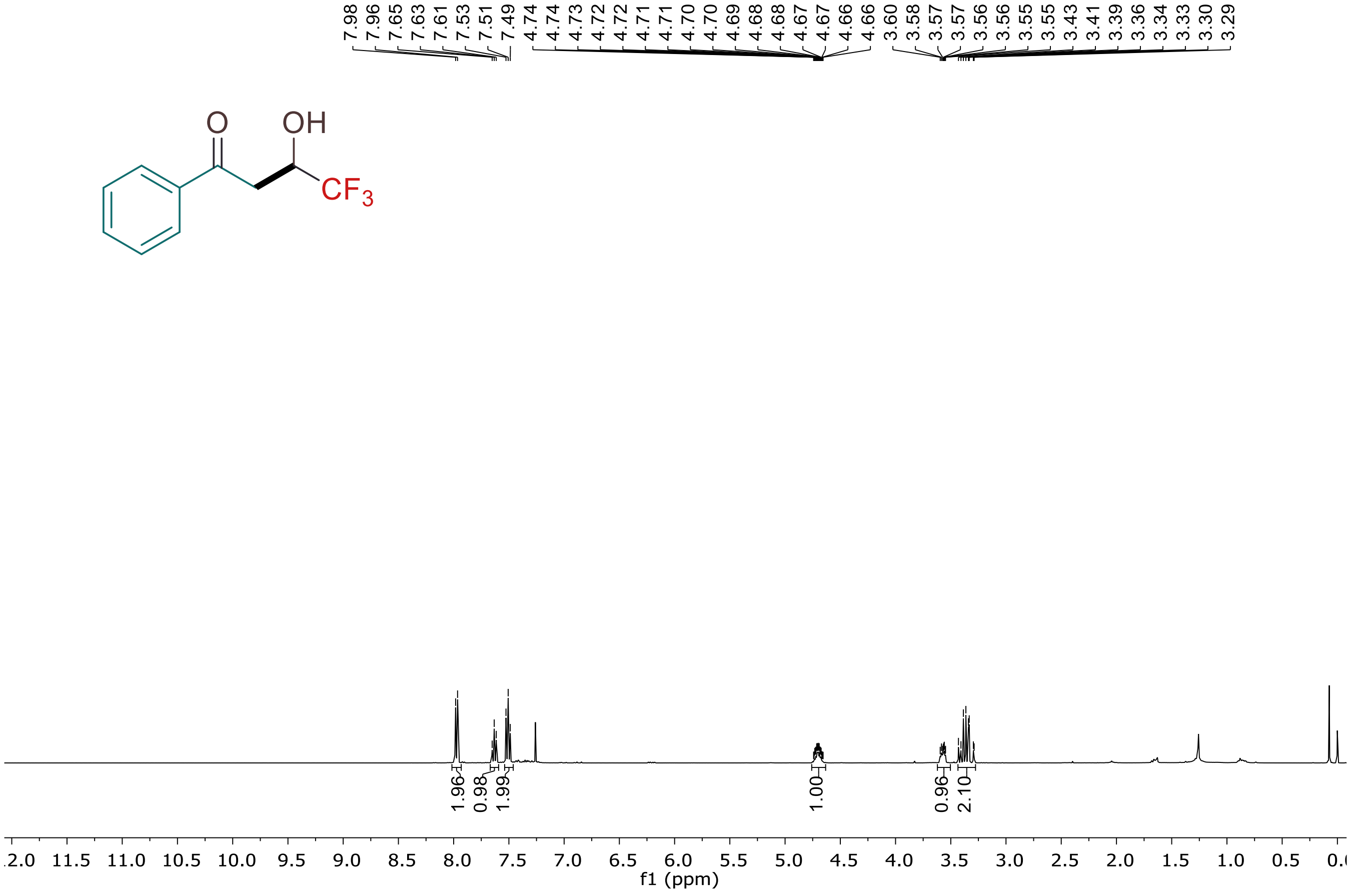

^{1}H NMR (400 MHz, $CDCl_3$) of compound **8**.

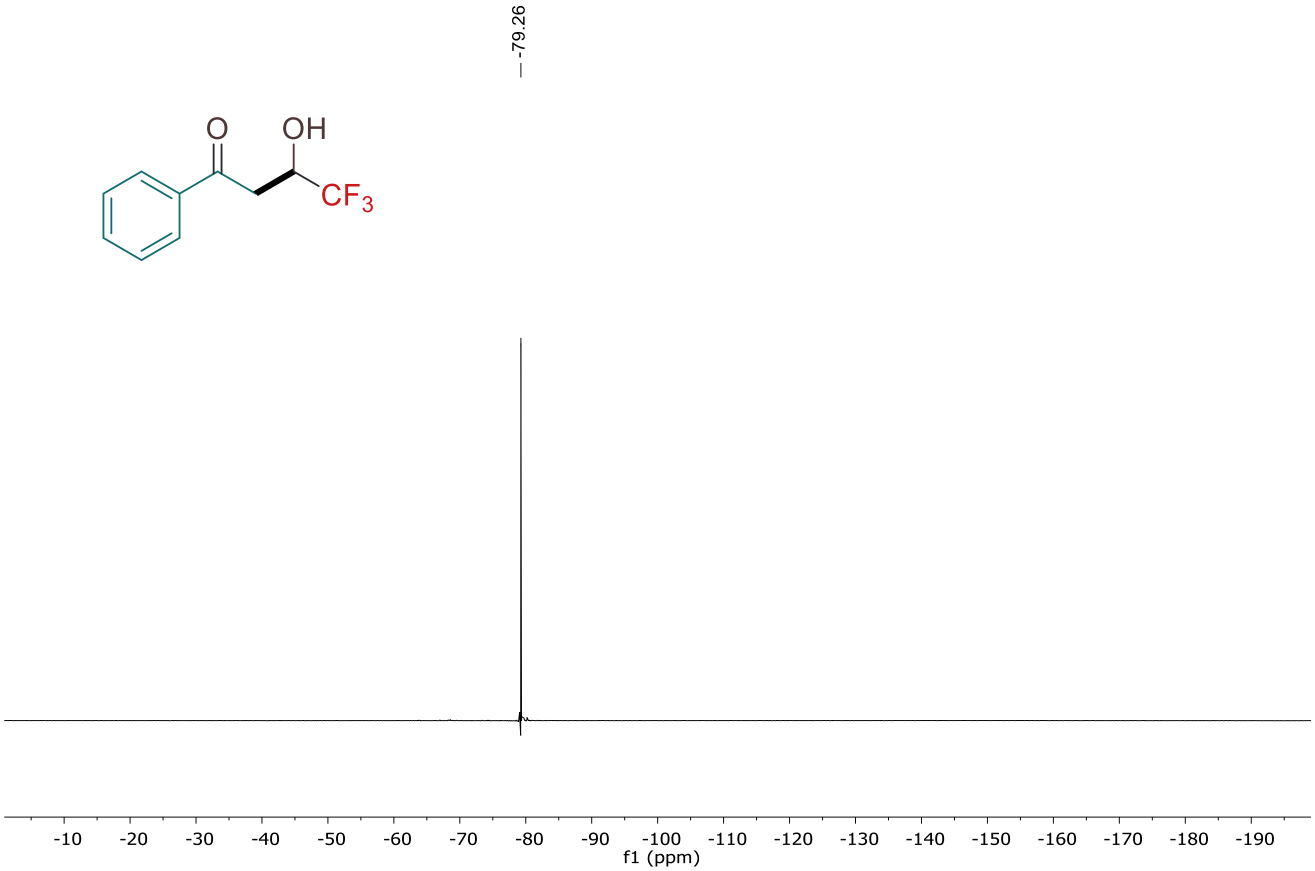

$^{19}F\{^{1}H\}$ NMR (377 MHz, $CDCl_3$) of compound **8**.

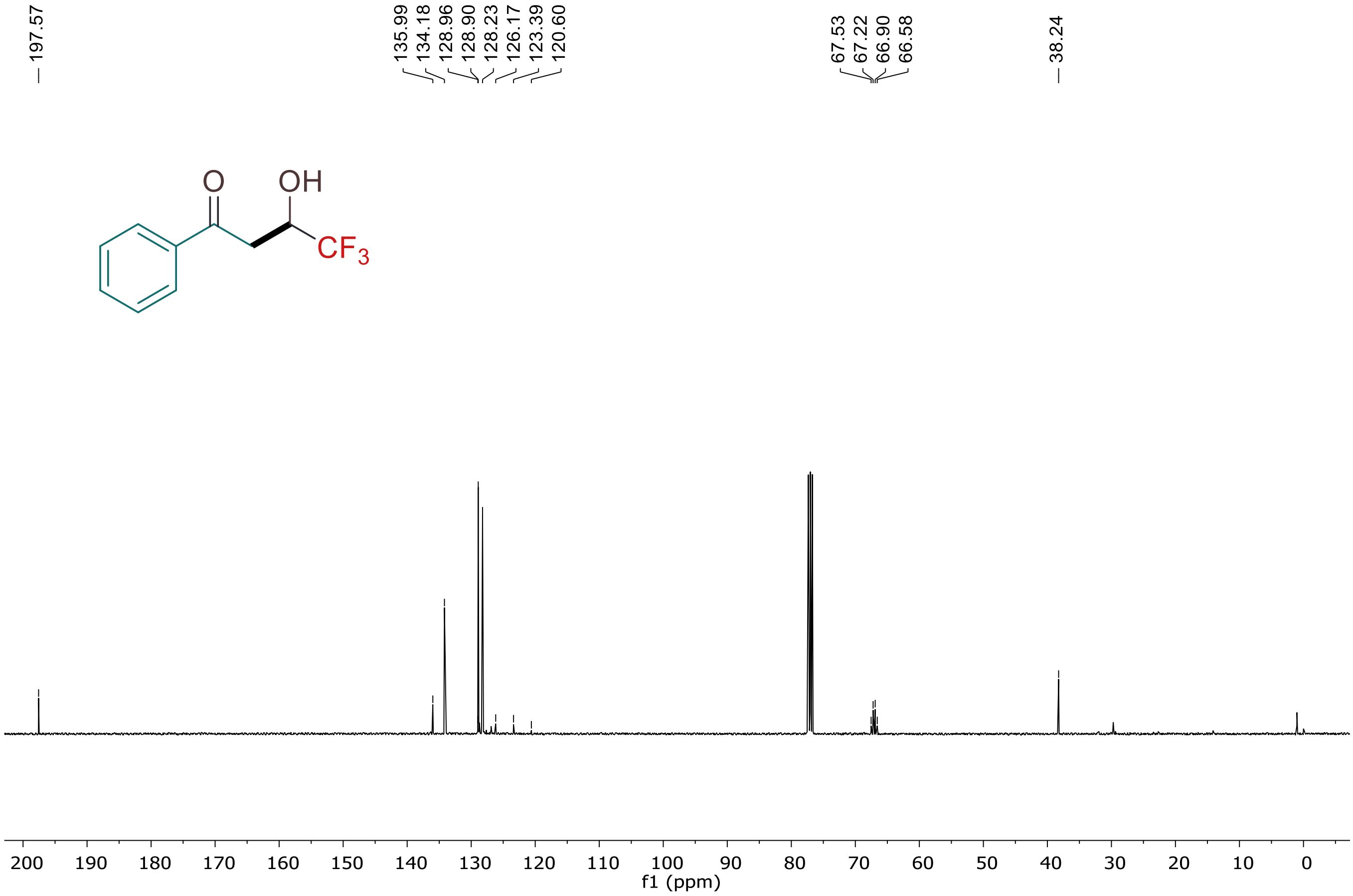

$^{13}C\{^{1}H\}$ NMR (101 MHz, $CDCl_3$) of compound **8**.

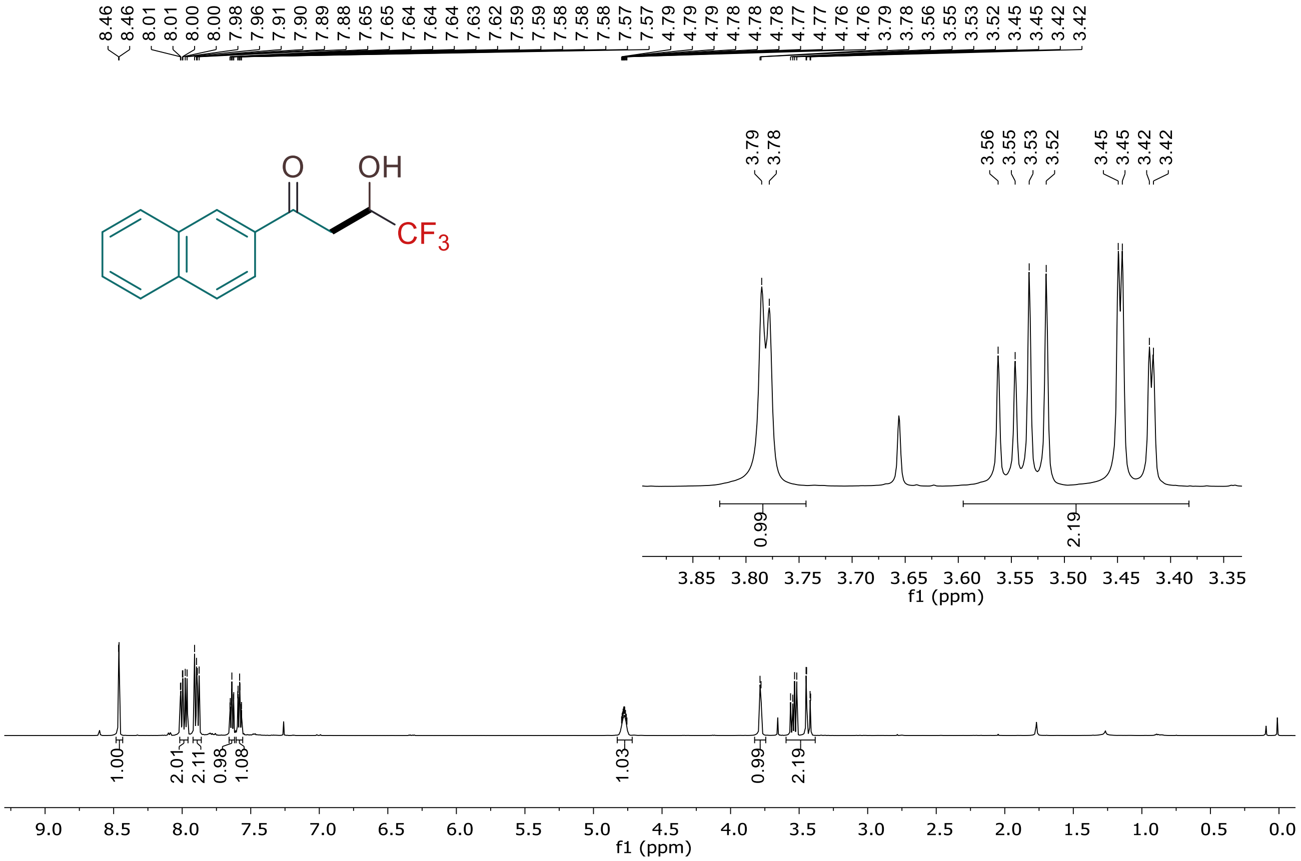

^{1}H NMR (600 MHz, $CDCl_3$) of compound **9**.

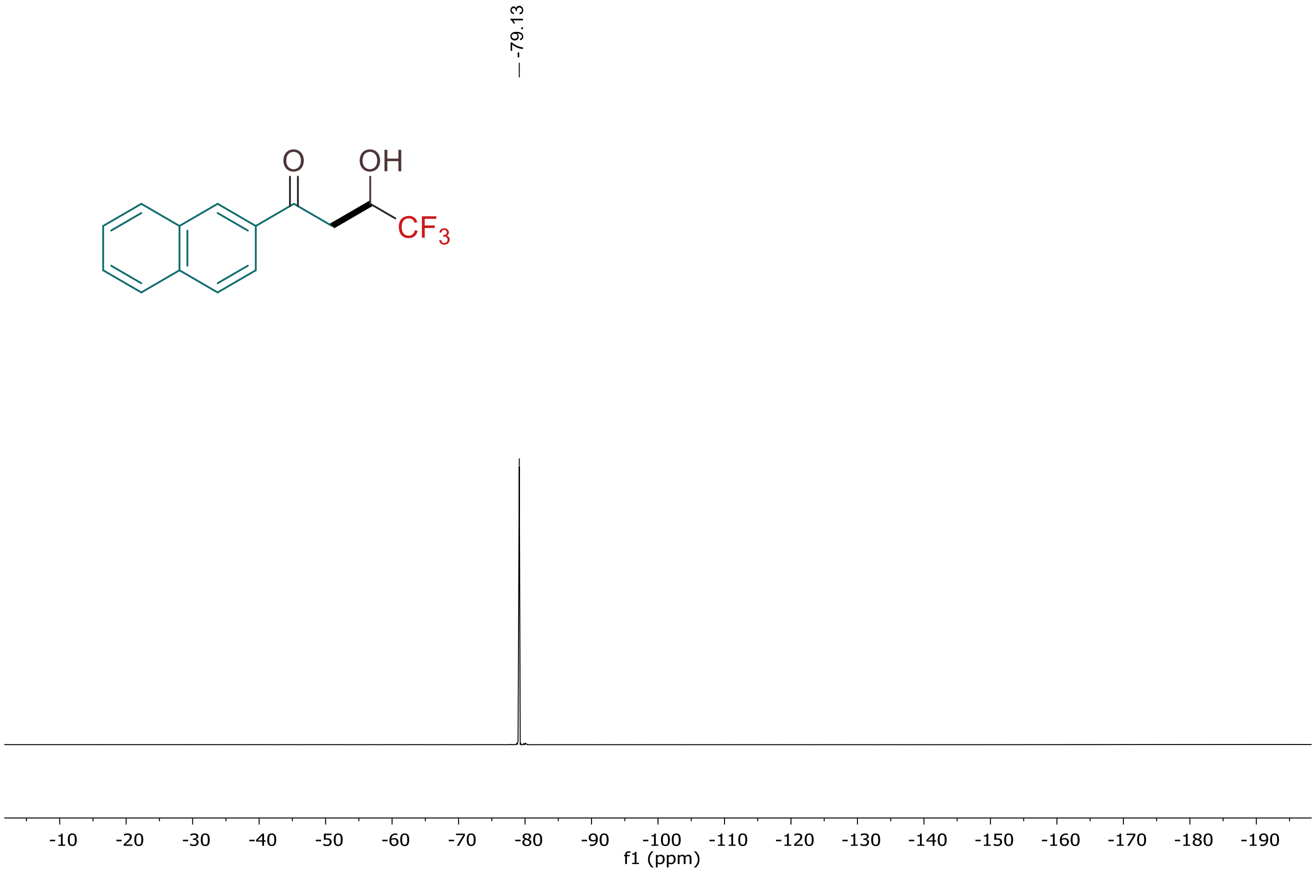

$^{19}F\{^{1}H\}$ NMR (282 MHz, $CDCl_3$) of compound **9**.

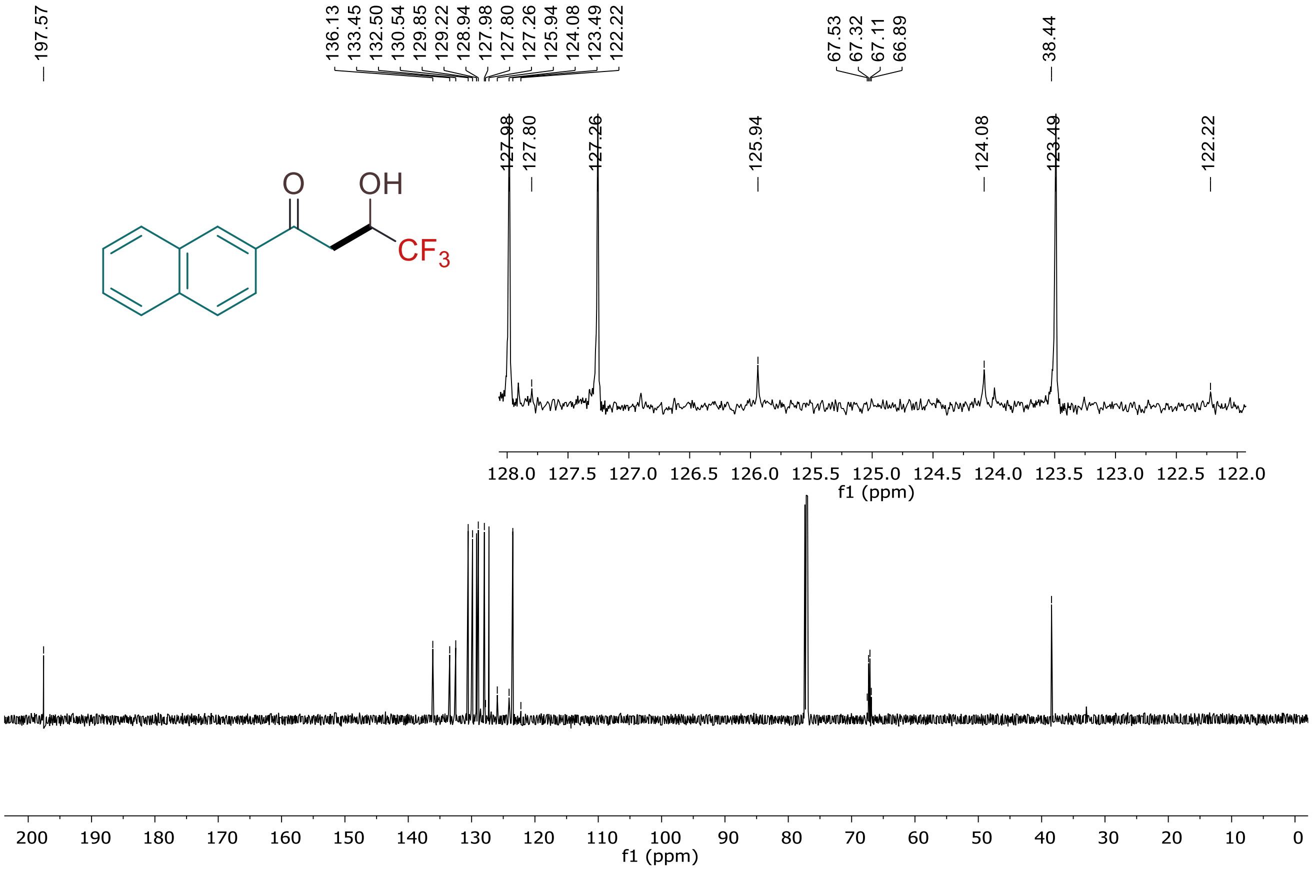

$^{13}C\{^{1}H\}$ NMR (151 MHz, $CDCl_3$) of compound **9**.

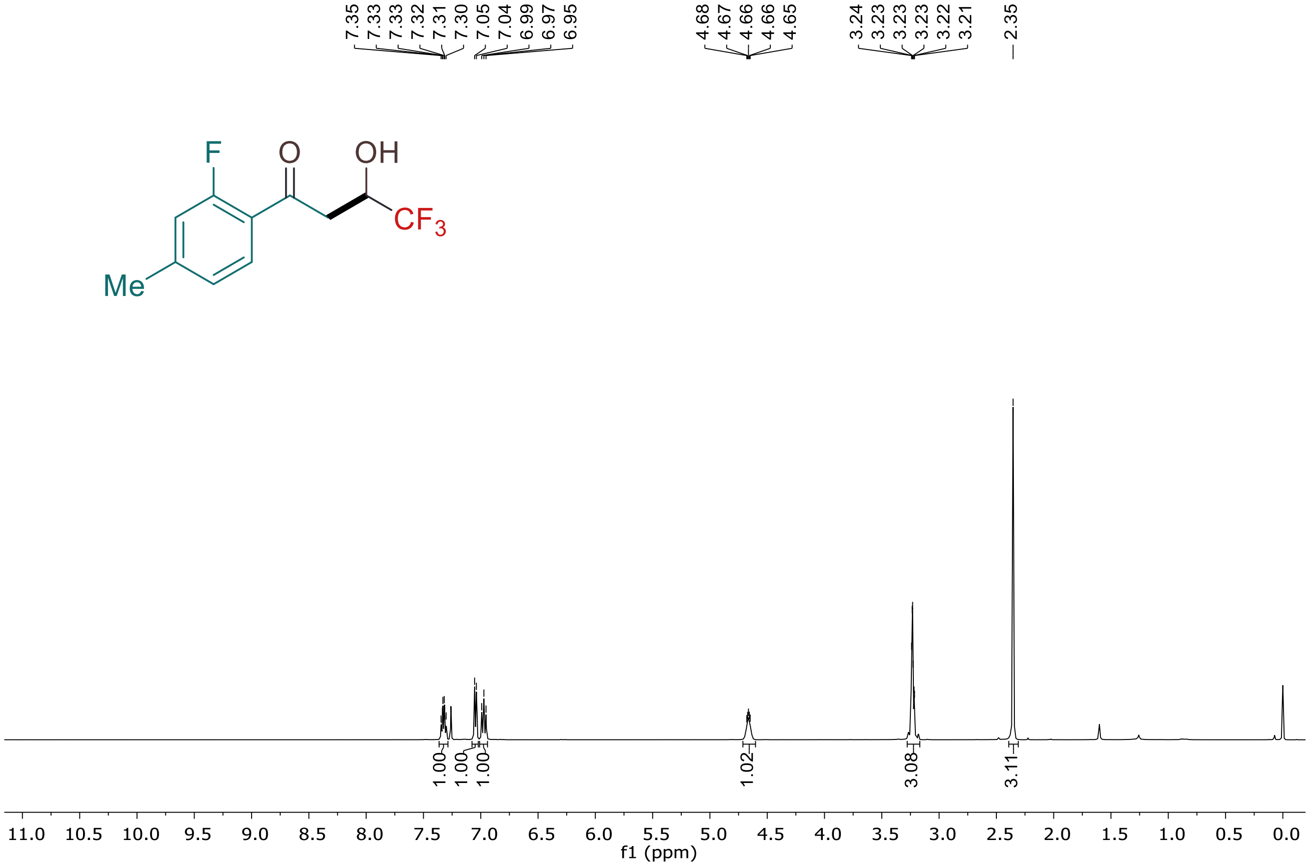

^{1}H NMR (500 MHz, $CDCl_3$) of compound **10**.

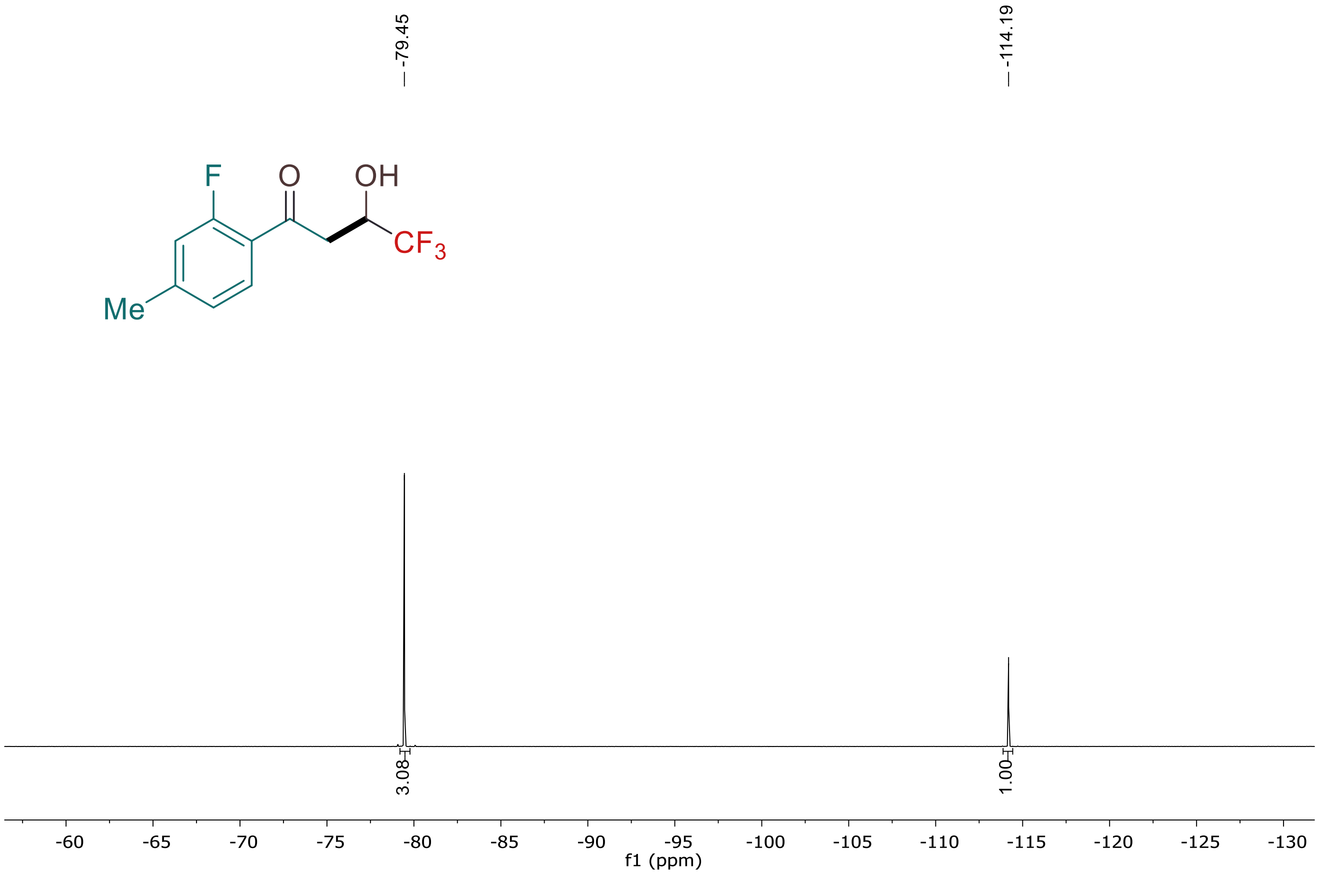

^{19}F{^{1}H} NMR (282 MHz, $CDCl_3$) of compound **10**.

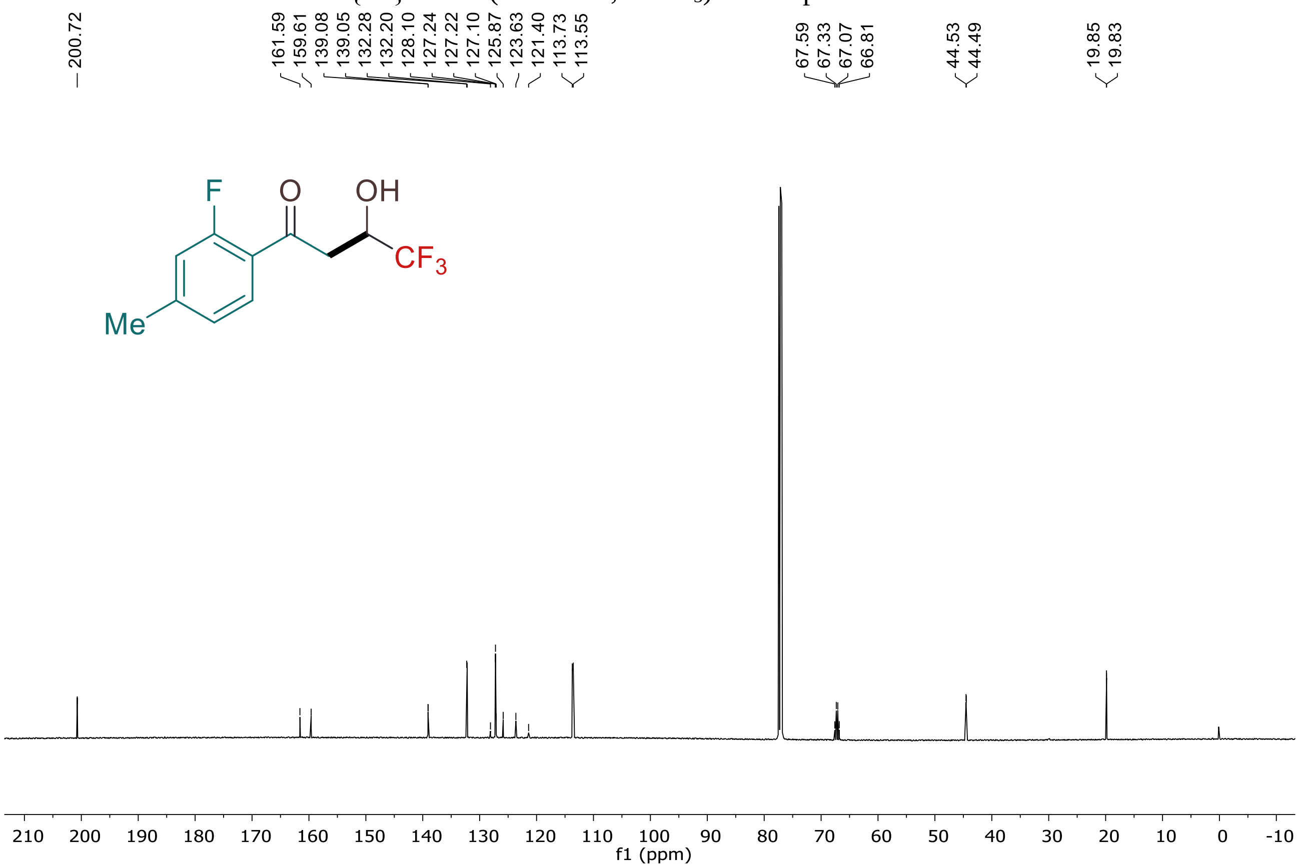

^{13}C{^{1}H} NMR (126 MHz, $CDCl_3$) of compound **10**.

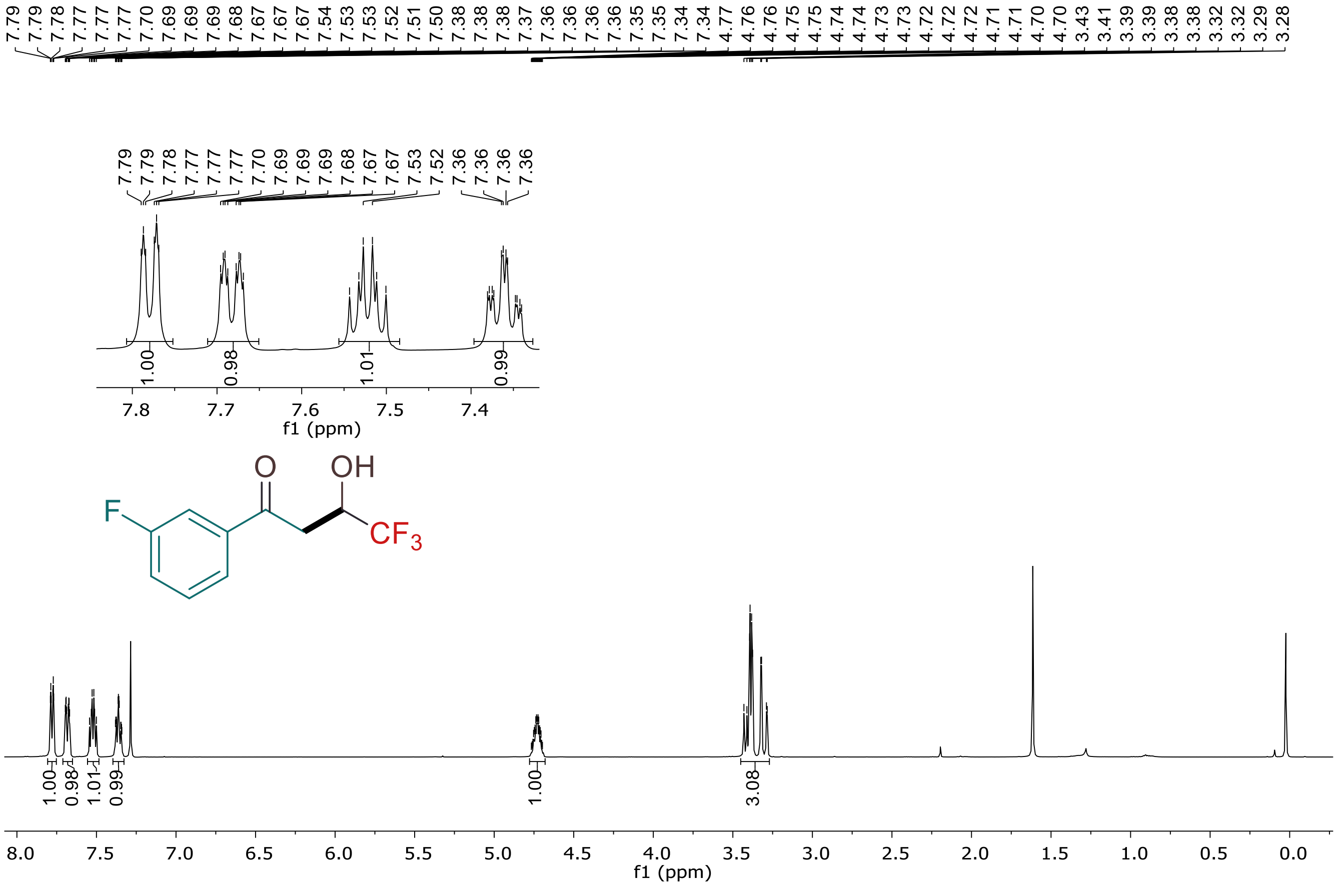

^{1}H NMR (500 MHz, $CDCl_3$) of compound **11**.

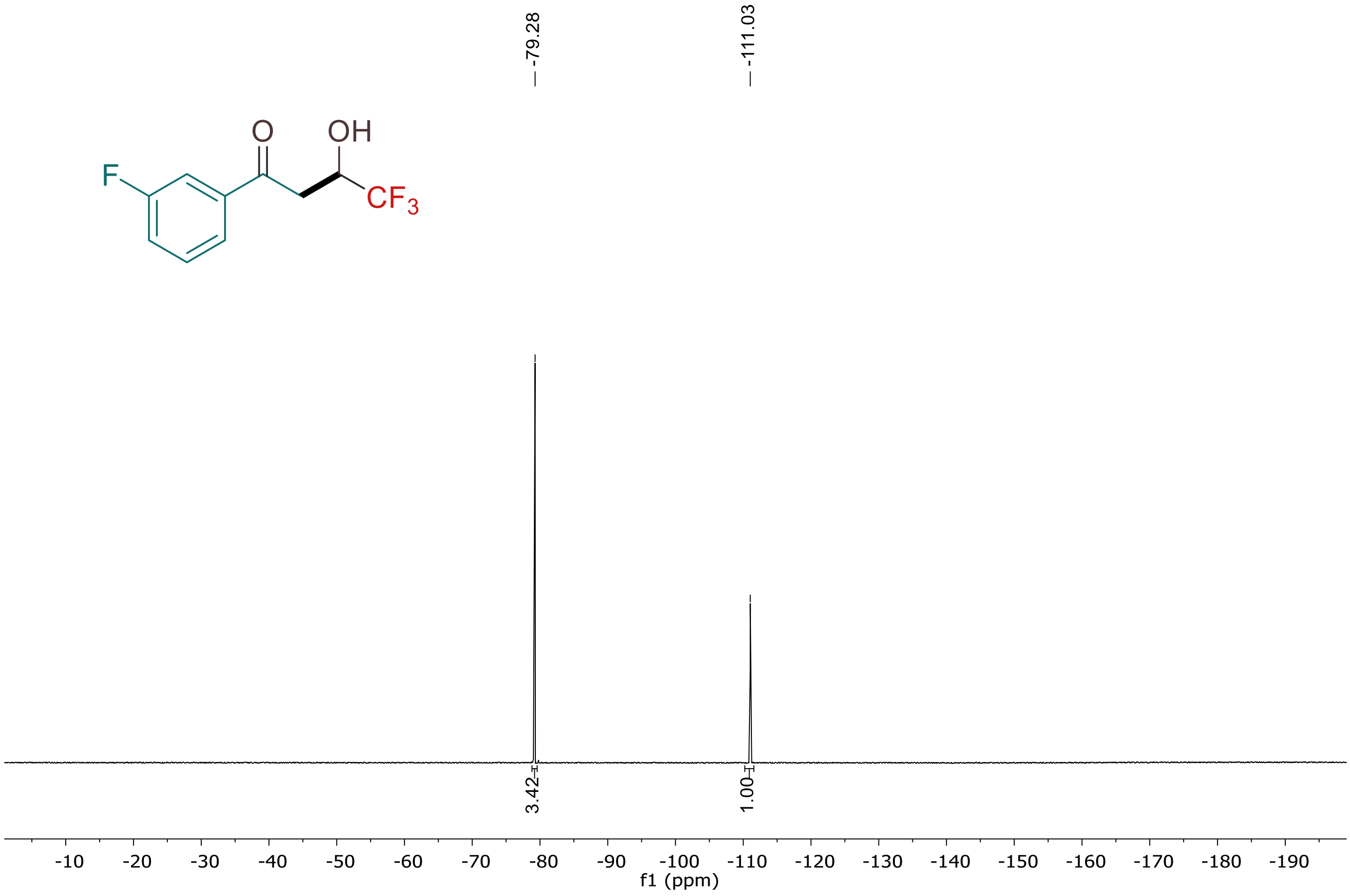

^{19}F{^{1}H} NMR (377 MHz, $CDCl_3$) of compound **11**.

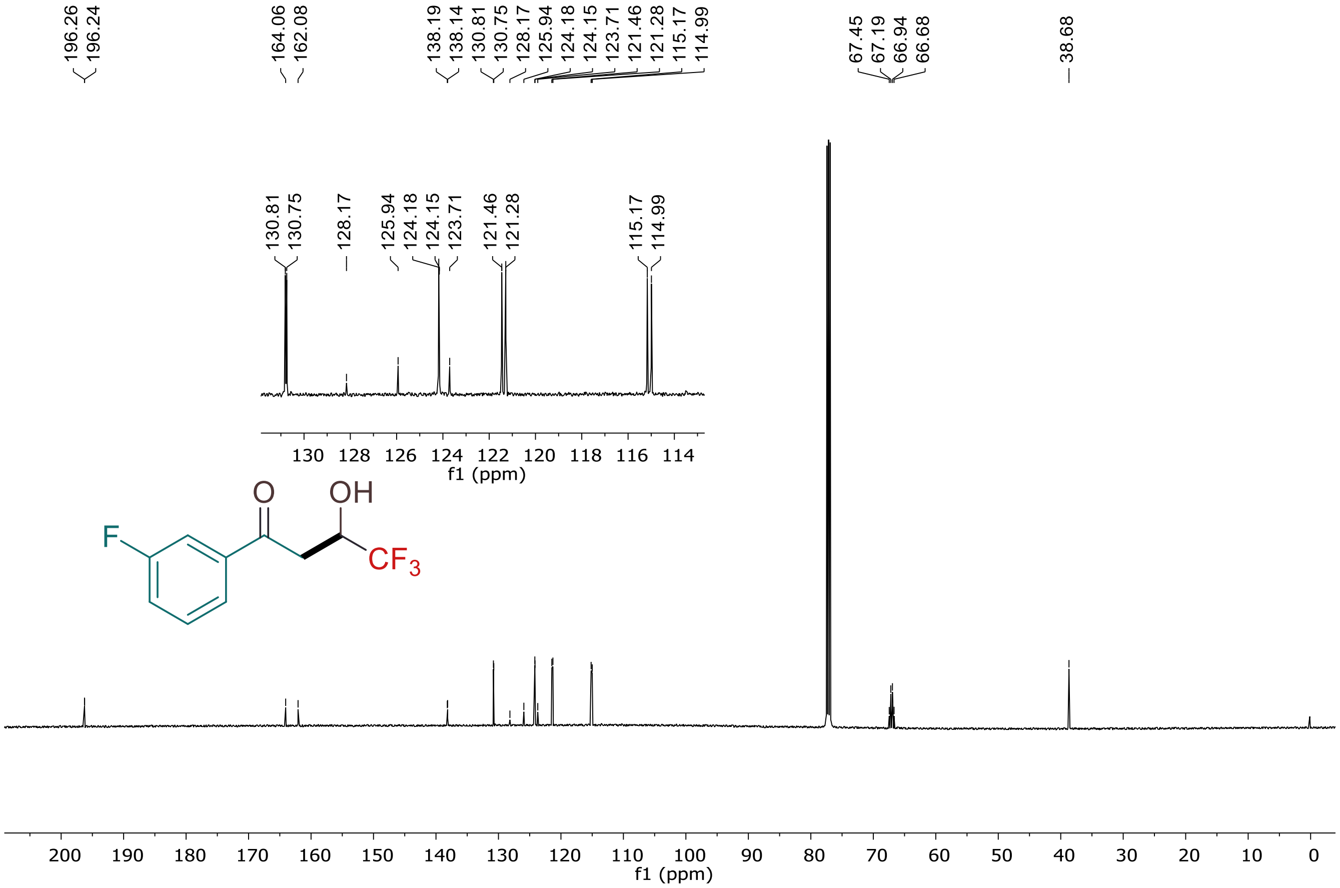

$^{13}C\{^{1}H\}$ NMR (126 MHz, $CDCl_3$) of compound **11**.

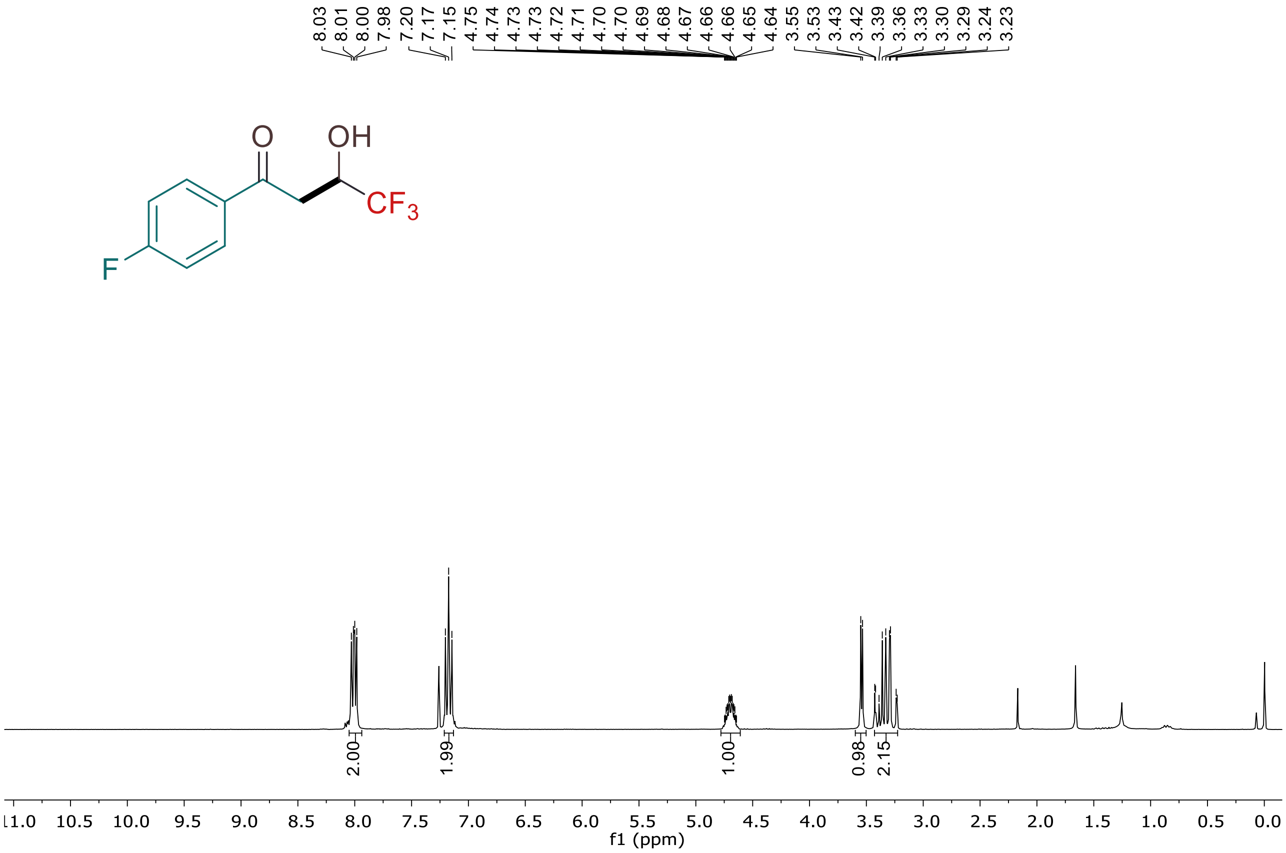

^{1}H NMR (400 MHz, $CDCl_3$) of compound **12**.

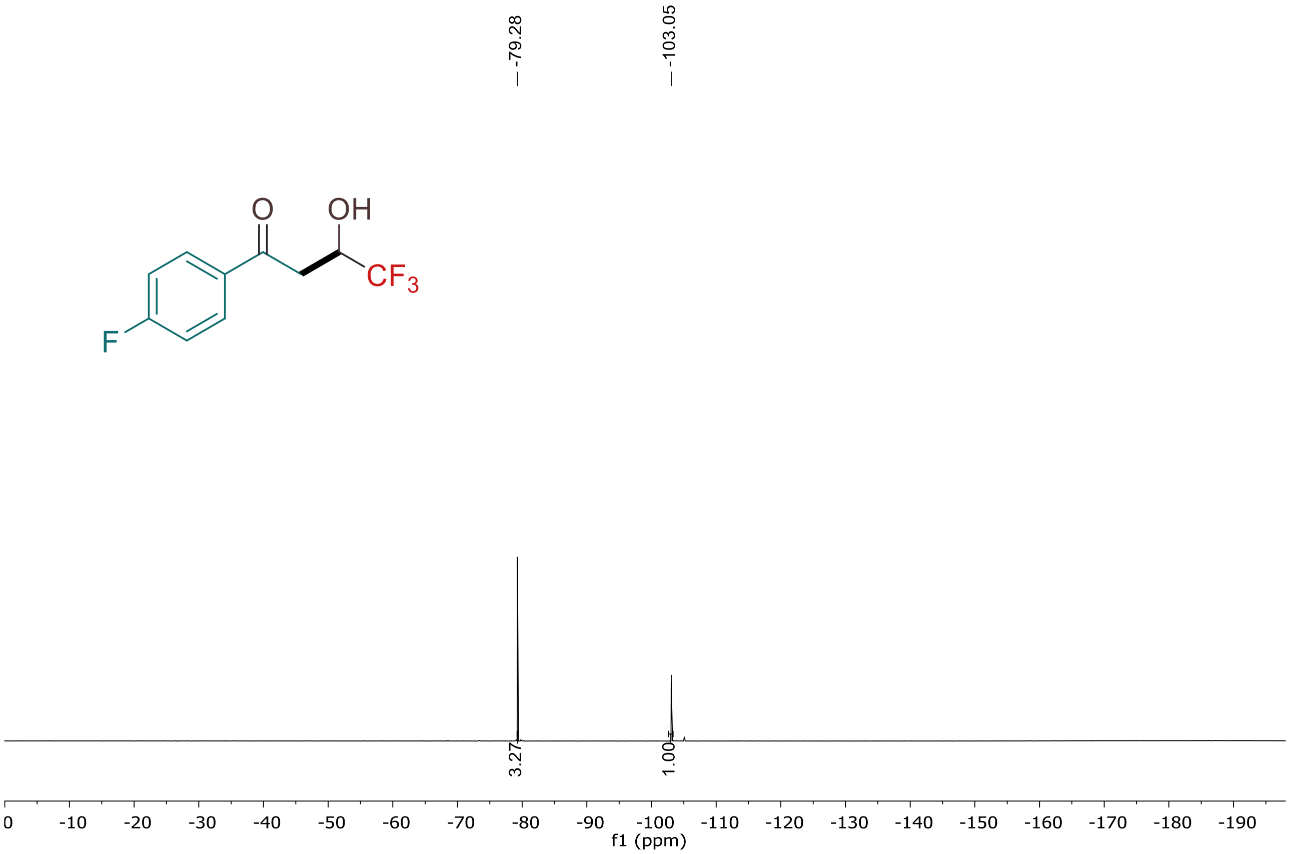

^{19}F{^{1}H} NMR (377 MHz, $CDCl_3$) of compound **12**.

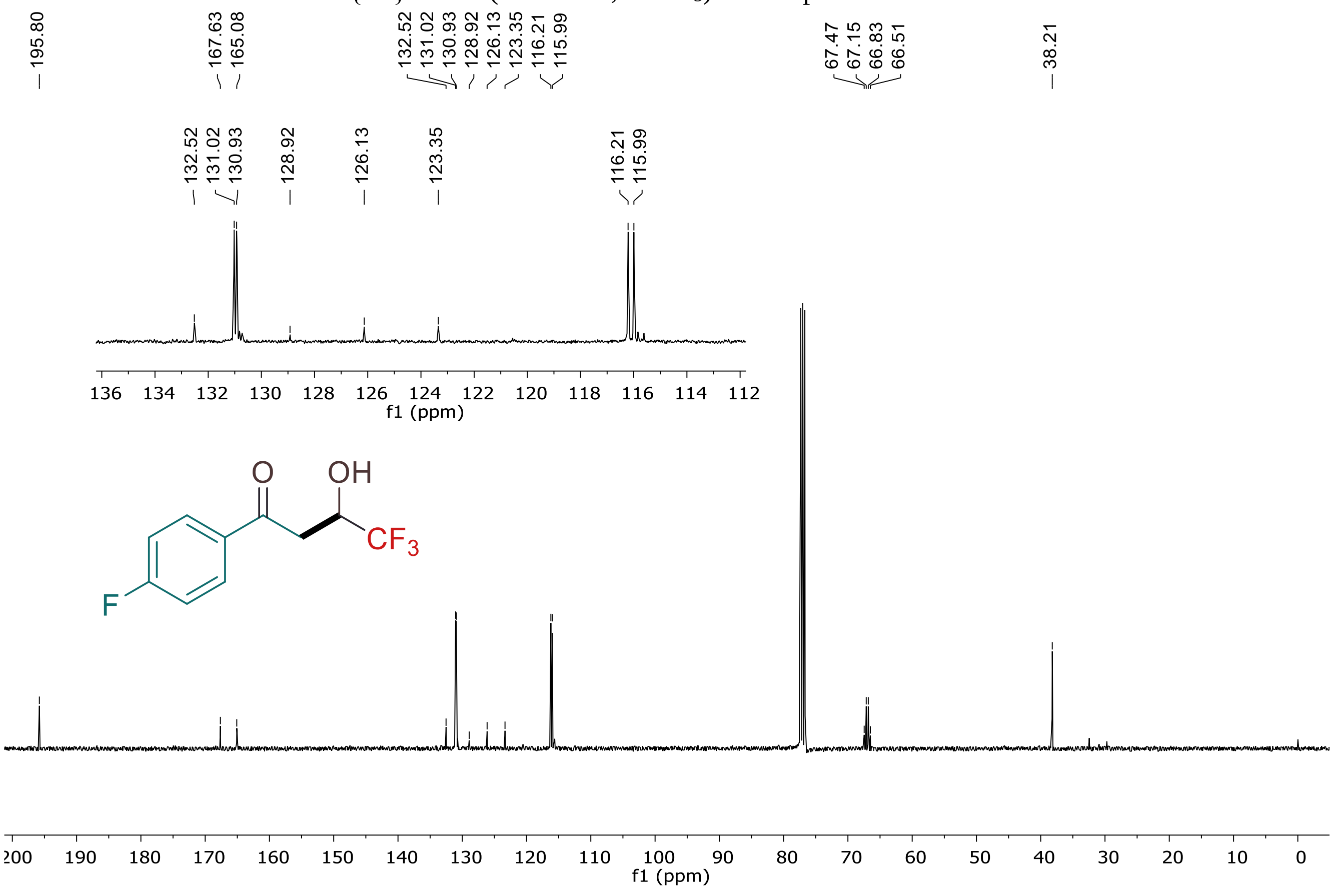

^{13}C{^{1}H} NMR (101 MHz, $CDCl_3$) of compound **12**.

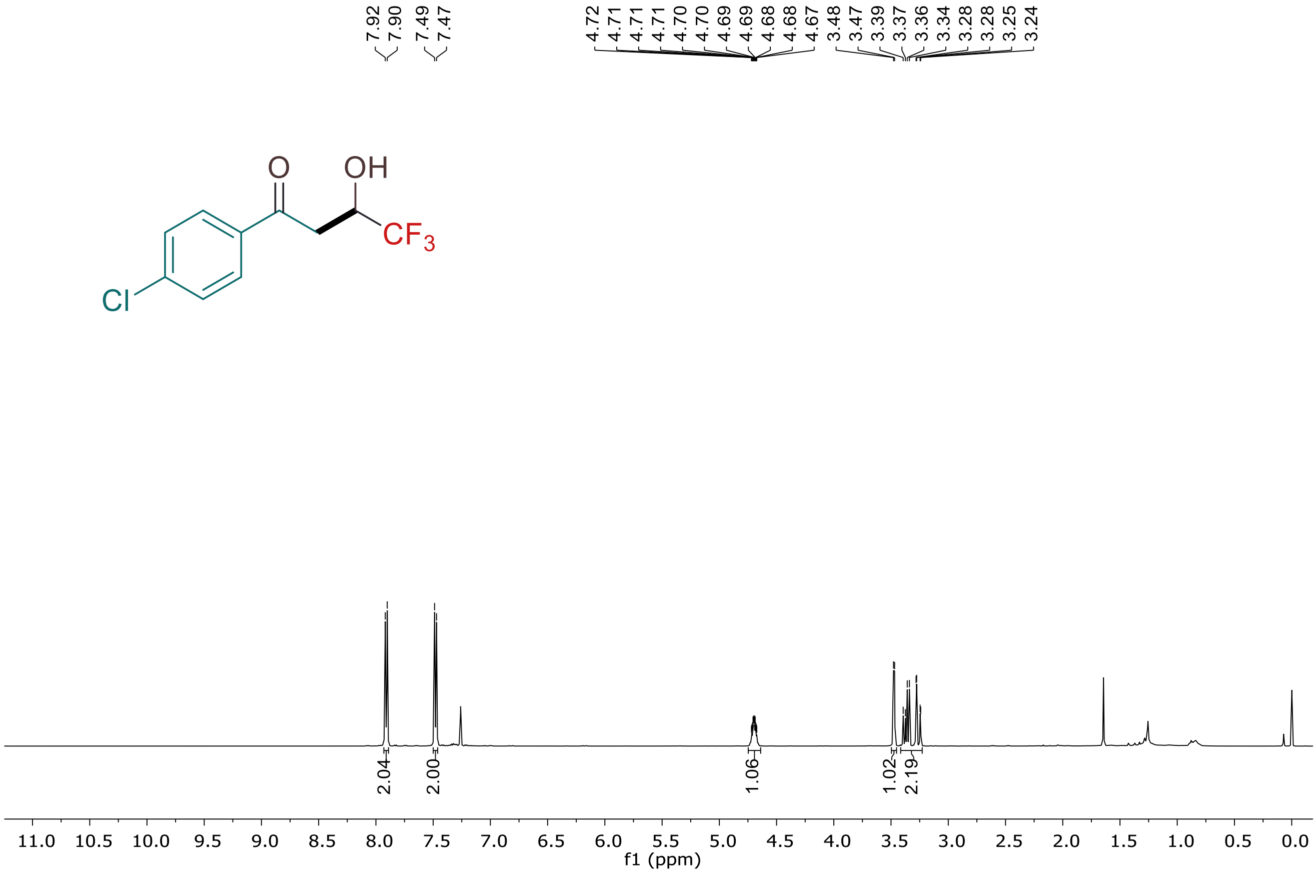

^{1}H NMR (500 MHz, $CDCl_3$) of compound **13**.

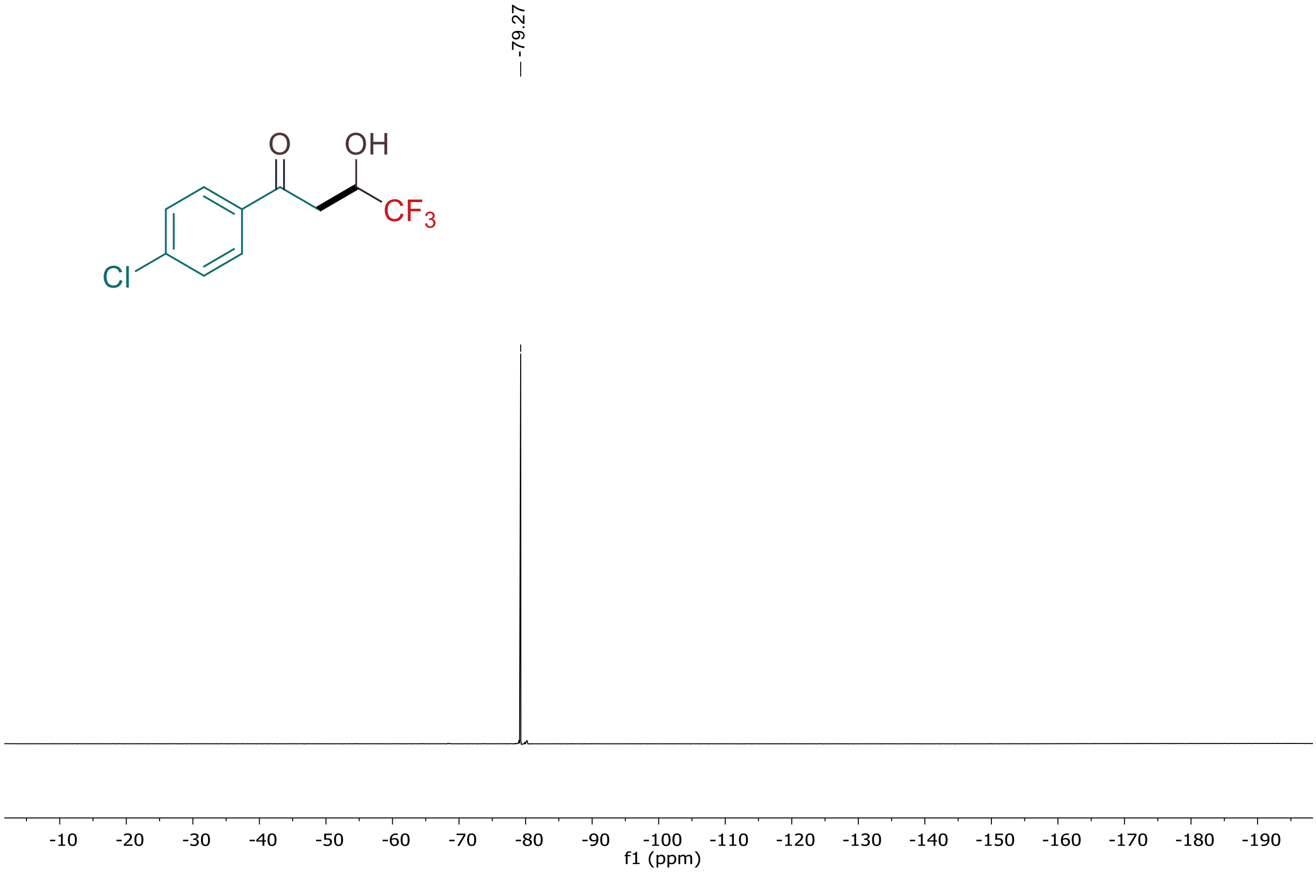

^{19}F{^{1}H} NMR (282 MHz, $CDCl_3$) of compound **13**.

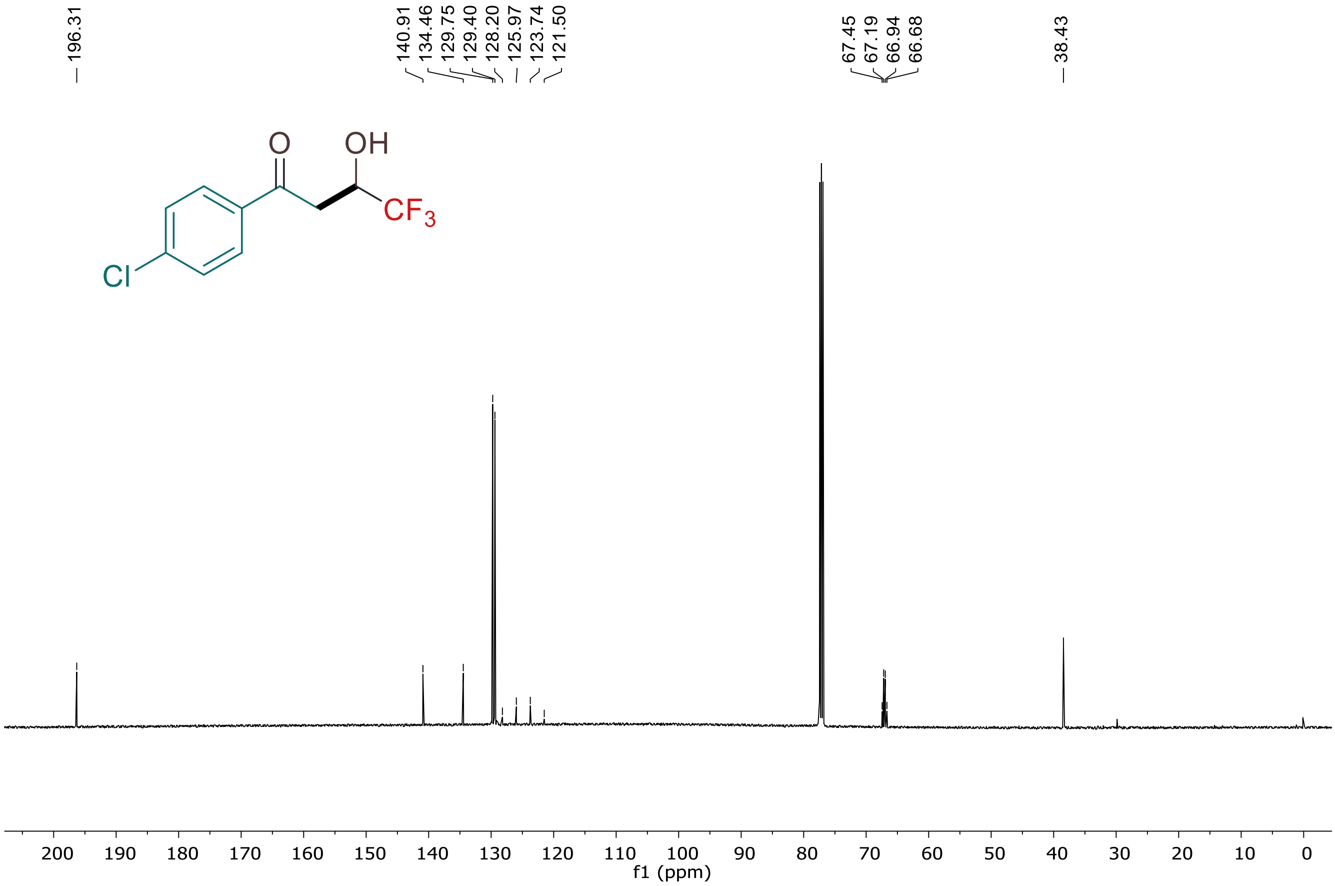

$^{13}C\{^{1}H\}$ NMR (126 MHz, $CDCl_3$) of compound **13**.

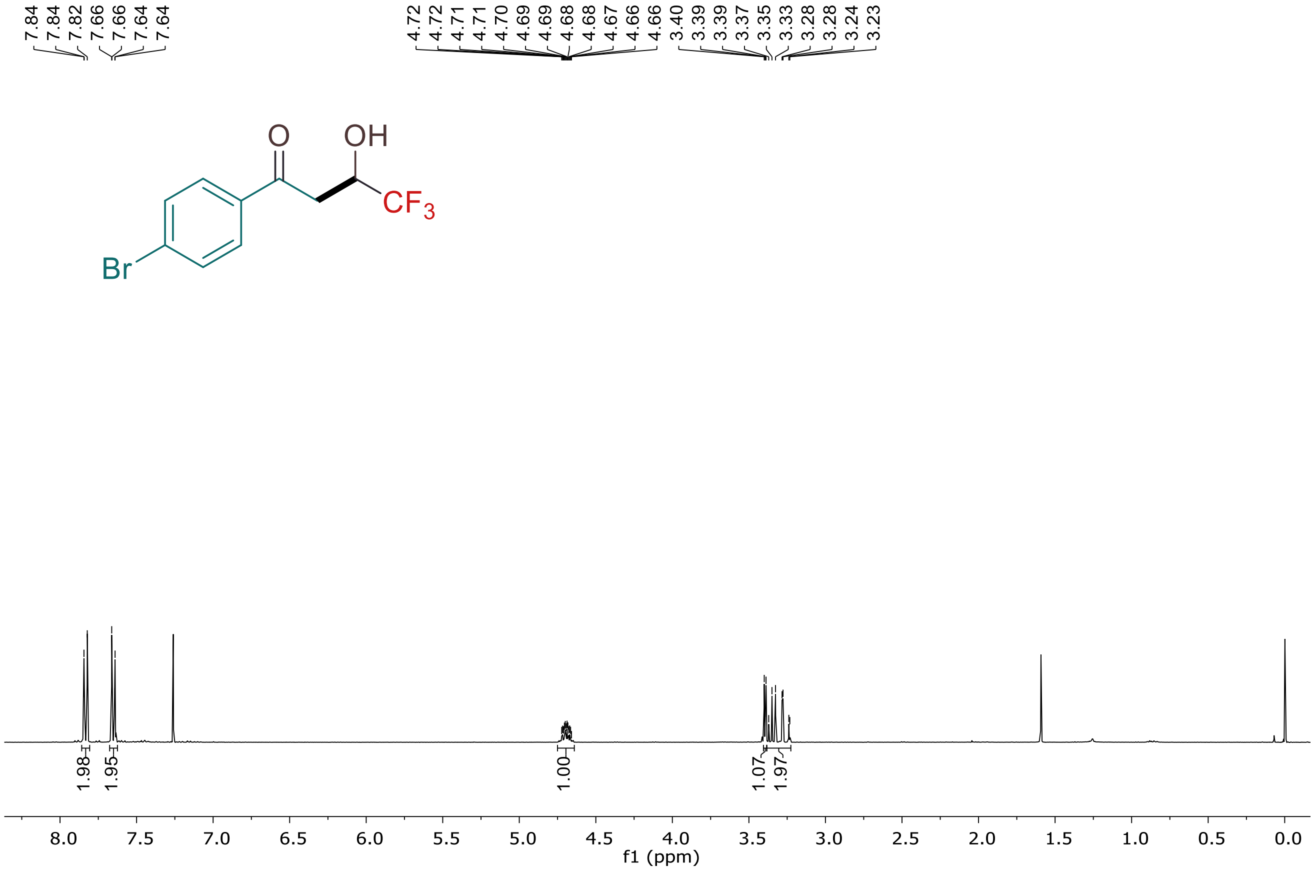

^{1}H NMR (400 MHz, $CDCl_3$) of compound **14**.

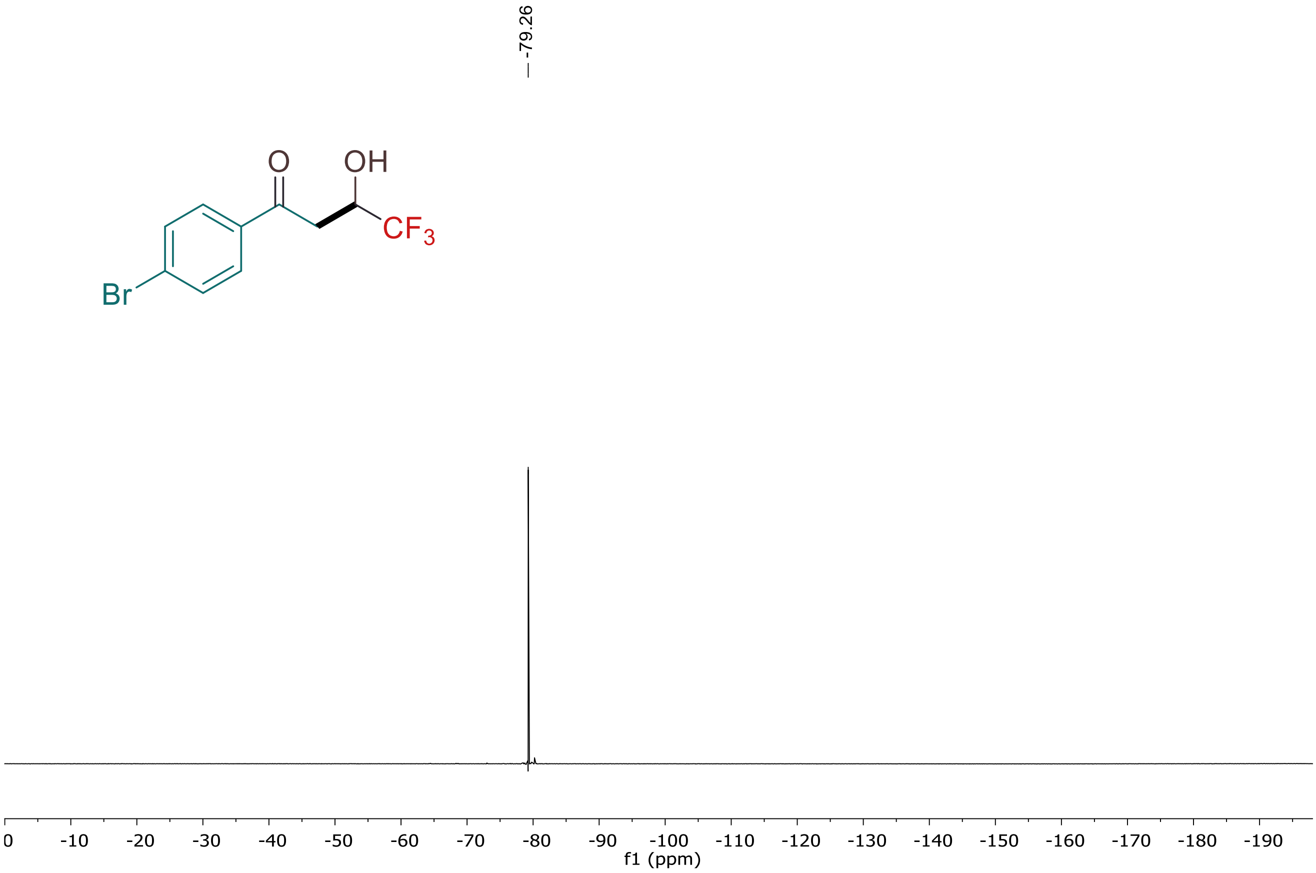

$^{19}F\{^1H\}$ NMR (377 MHz, $CDCl_3$) of compound **14**.

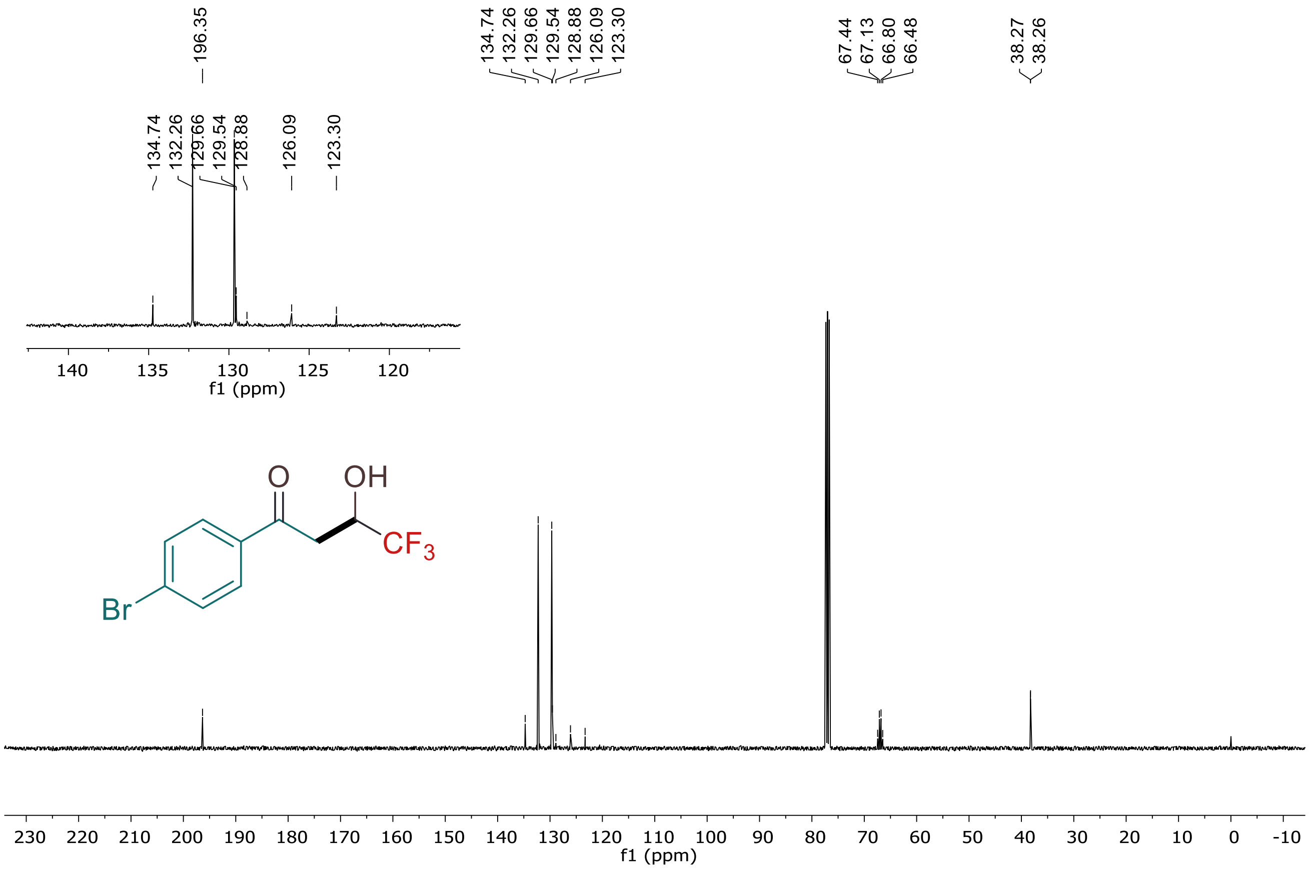

$^{13}C\{^1H\}$ NMR (101 MHz, $CDCl_3$) of compound **14**.

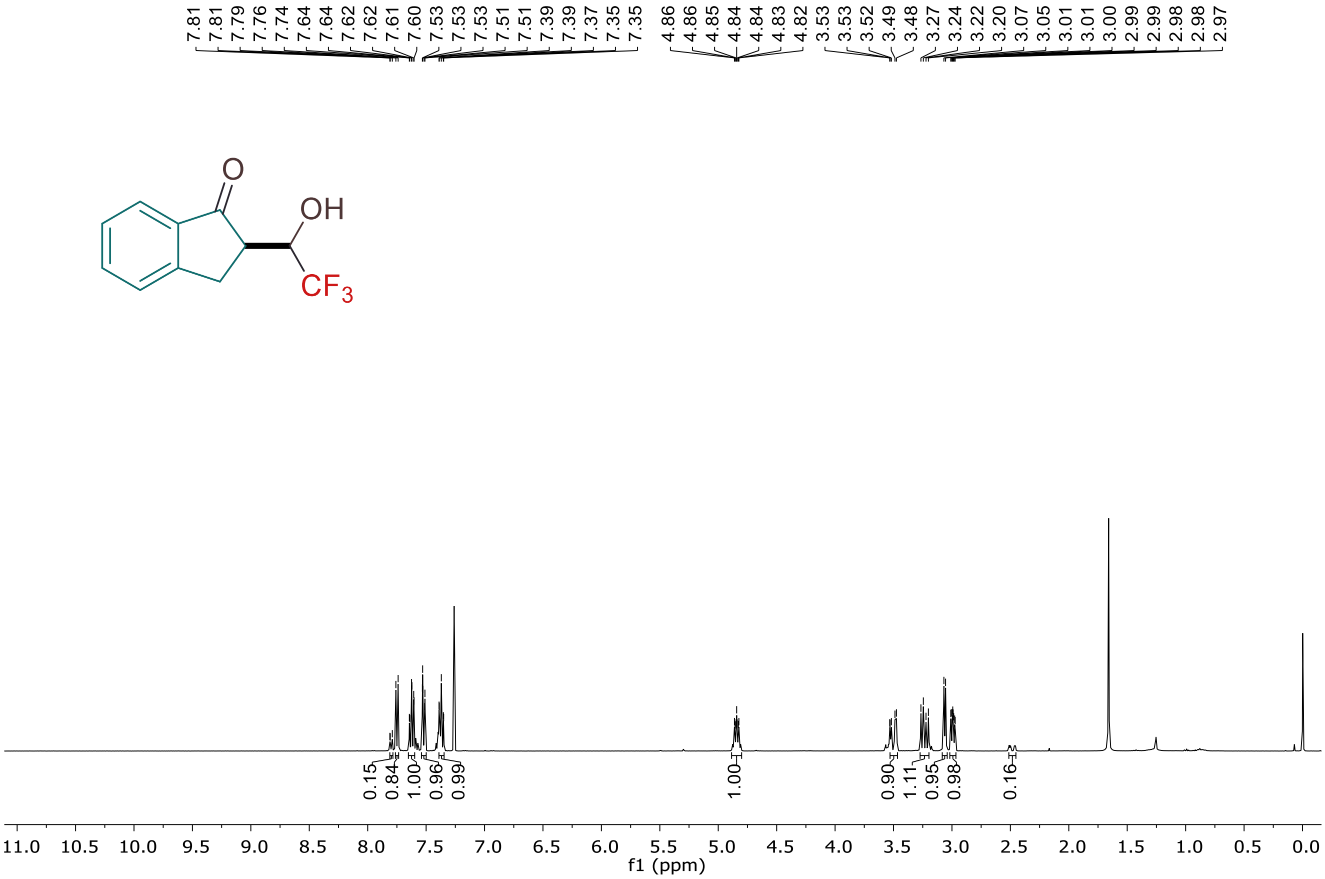

^{1}H NMR (400 MHz, $CDCl_3$) of compound **15**.

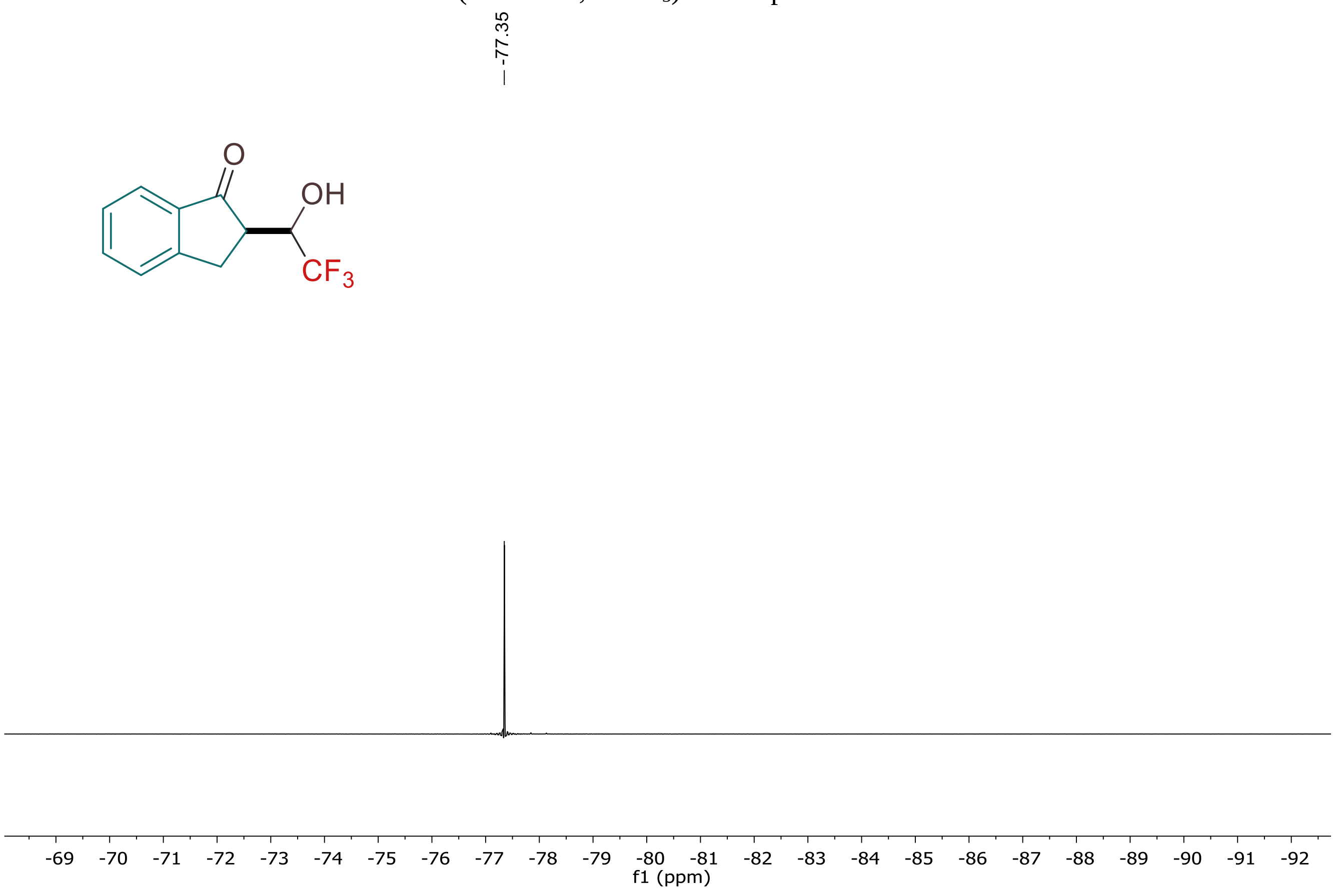

^{19}F{^{1}H} NMR (377 MHz, $CDCl_3$) of compound **15**.

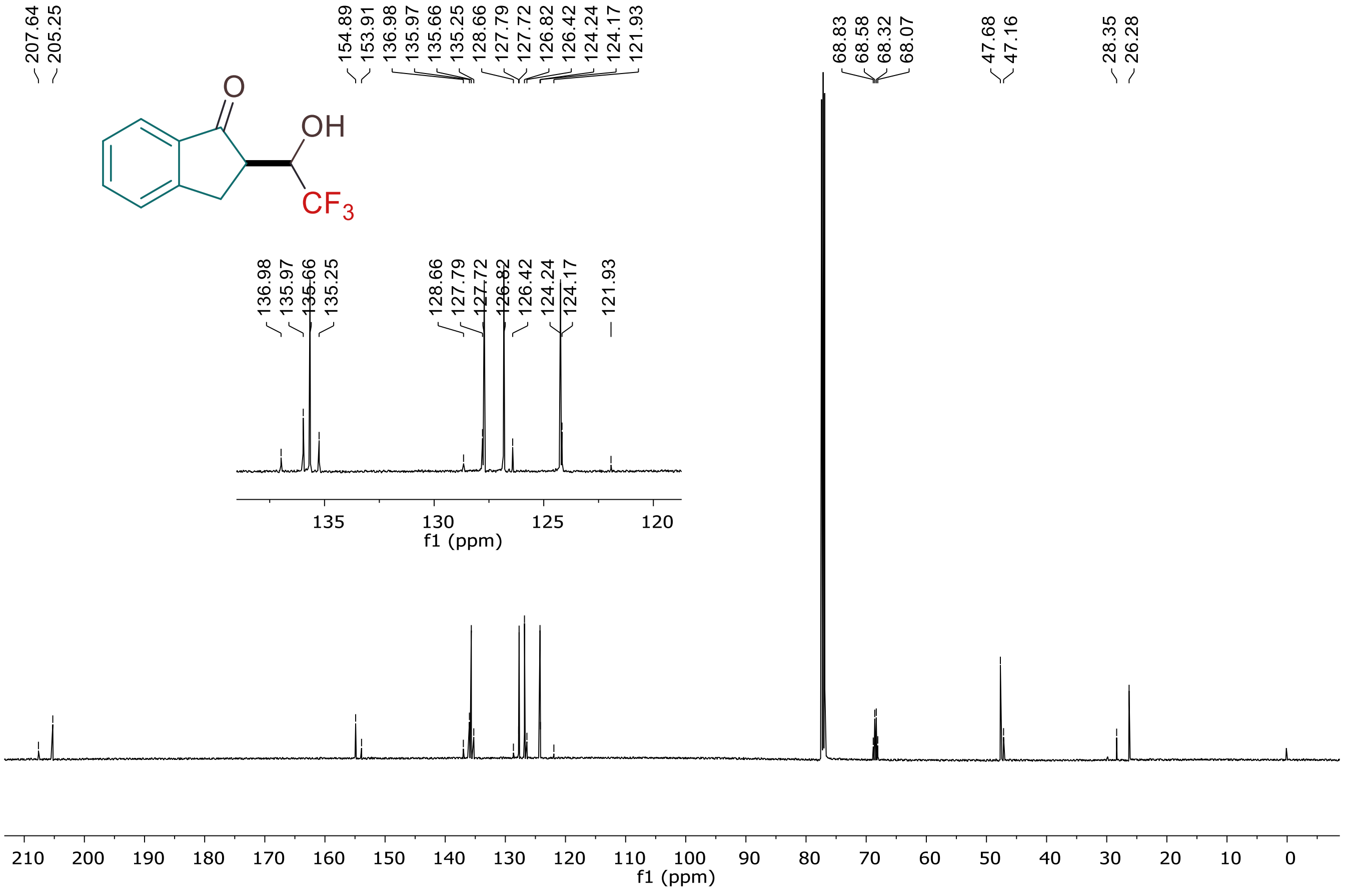

$^{13}C\{^{1}H\}$ NMR (126 MHz, $CDCl_3$) of compound **15**.

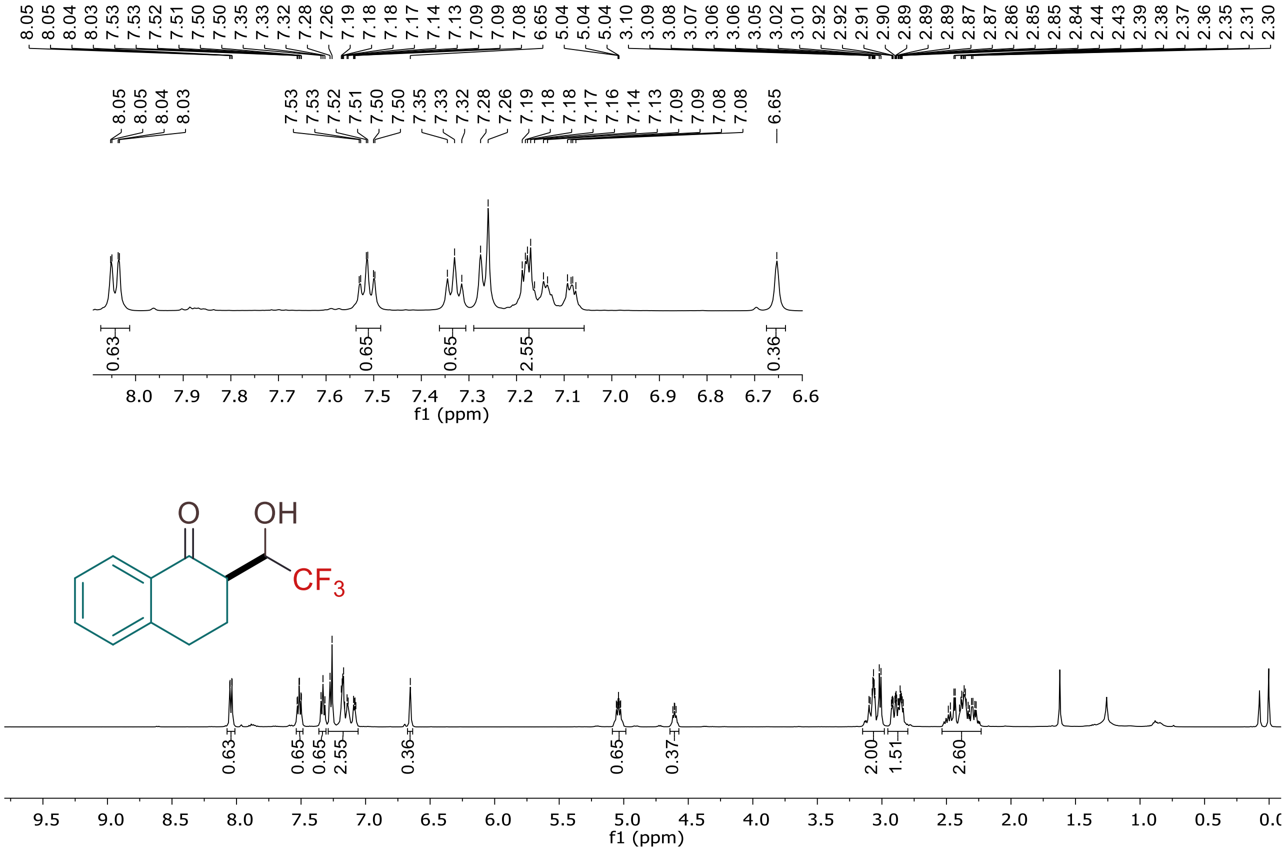

^{1}H NMR (500 MHz, $CDCl_3$) of compound **16**.

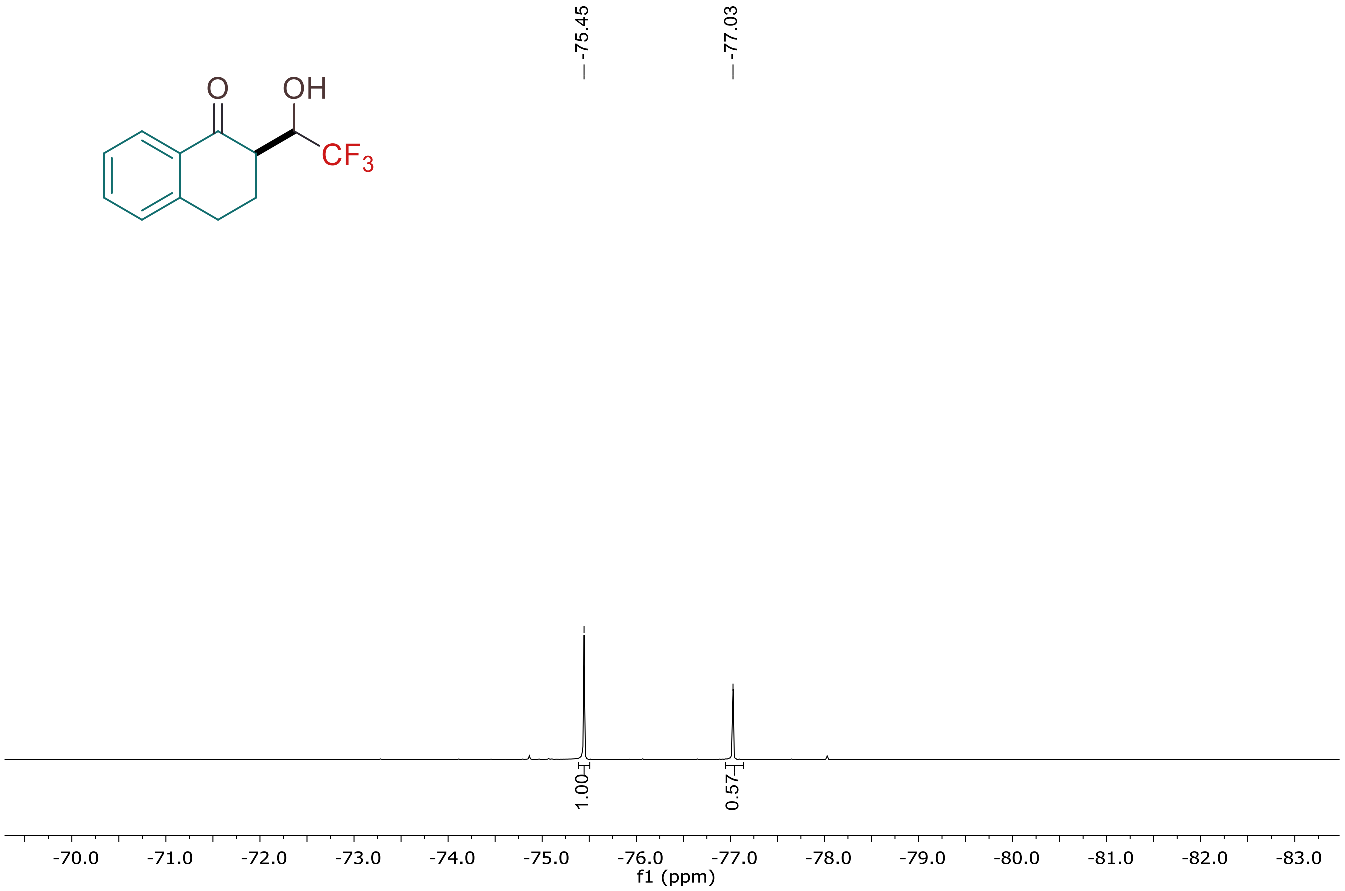

$^{19}F\{^{1}H\}$ NMR (282 MHz, $CDCl_3$) of compound **16**.

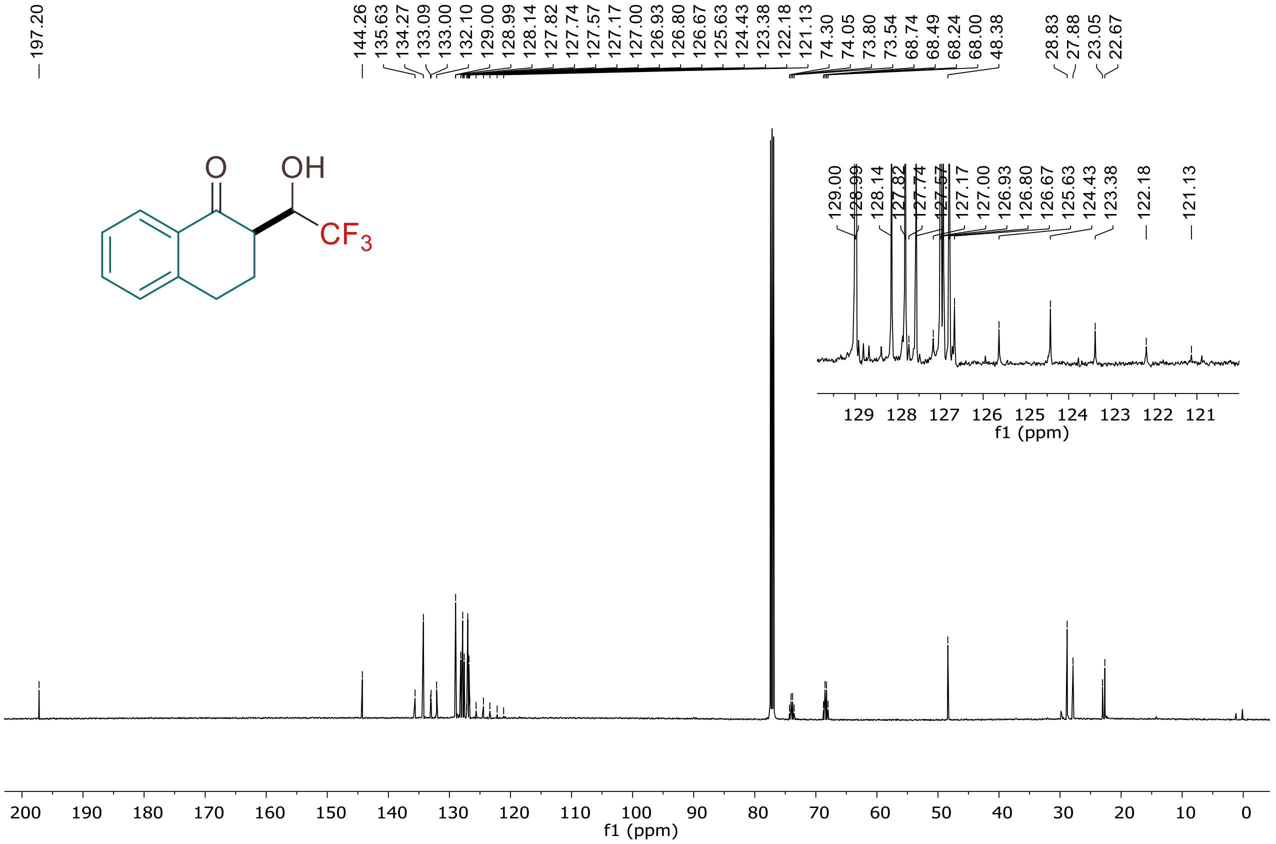

$^{13}C\{^{1}H\}$ NMR (126 MHz, $CDCl_3$) of compound **16**.

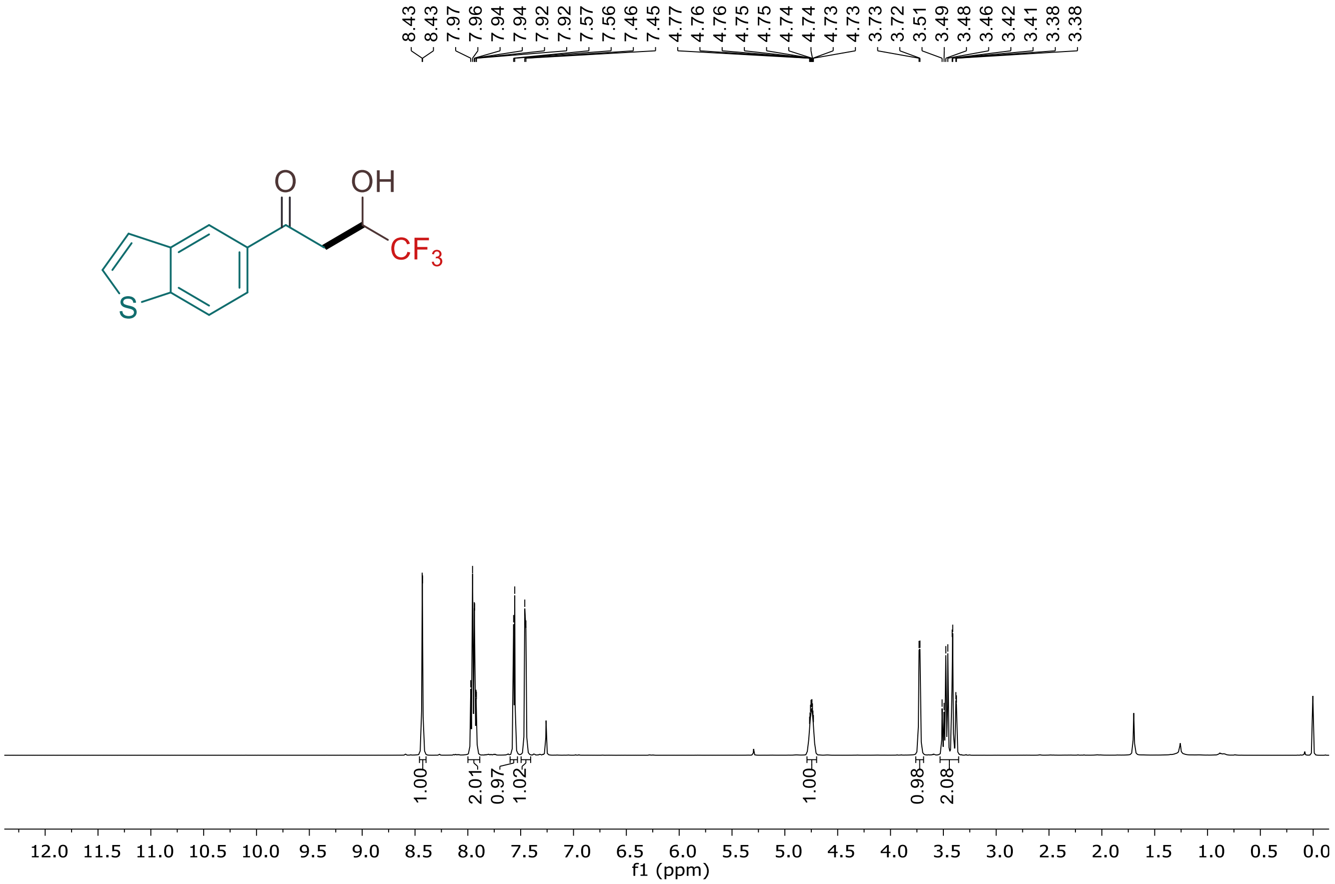

^{1}H NMR (500 MHz, $CDCl_3$) of compound **17**.

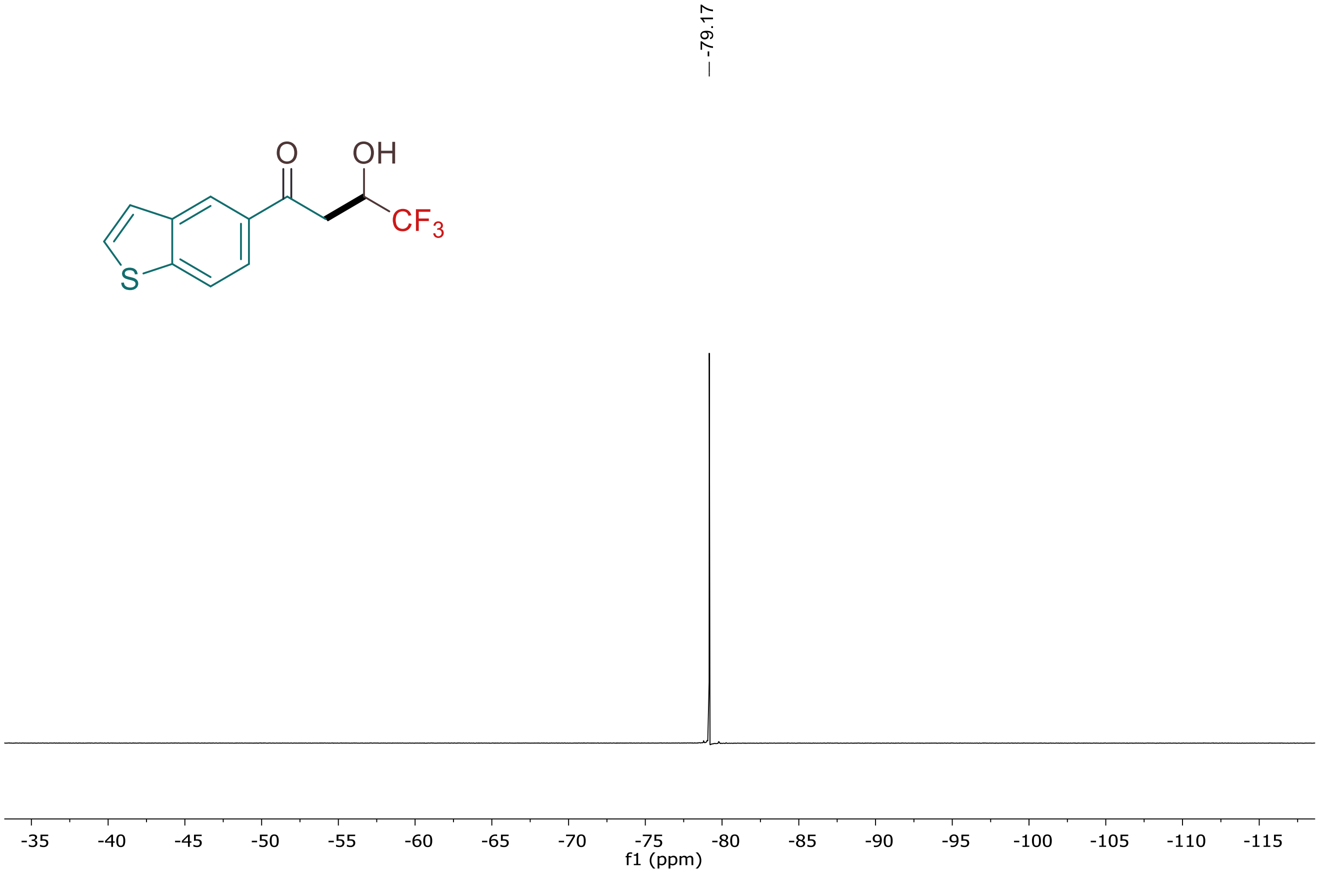

$^{19}F\{^{1}H\}$ NMR (282 MHz, $CDCl_3$) of compound **17**.

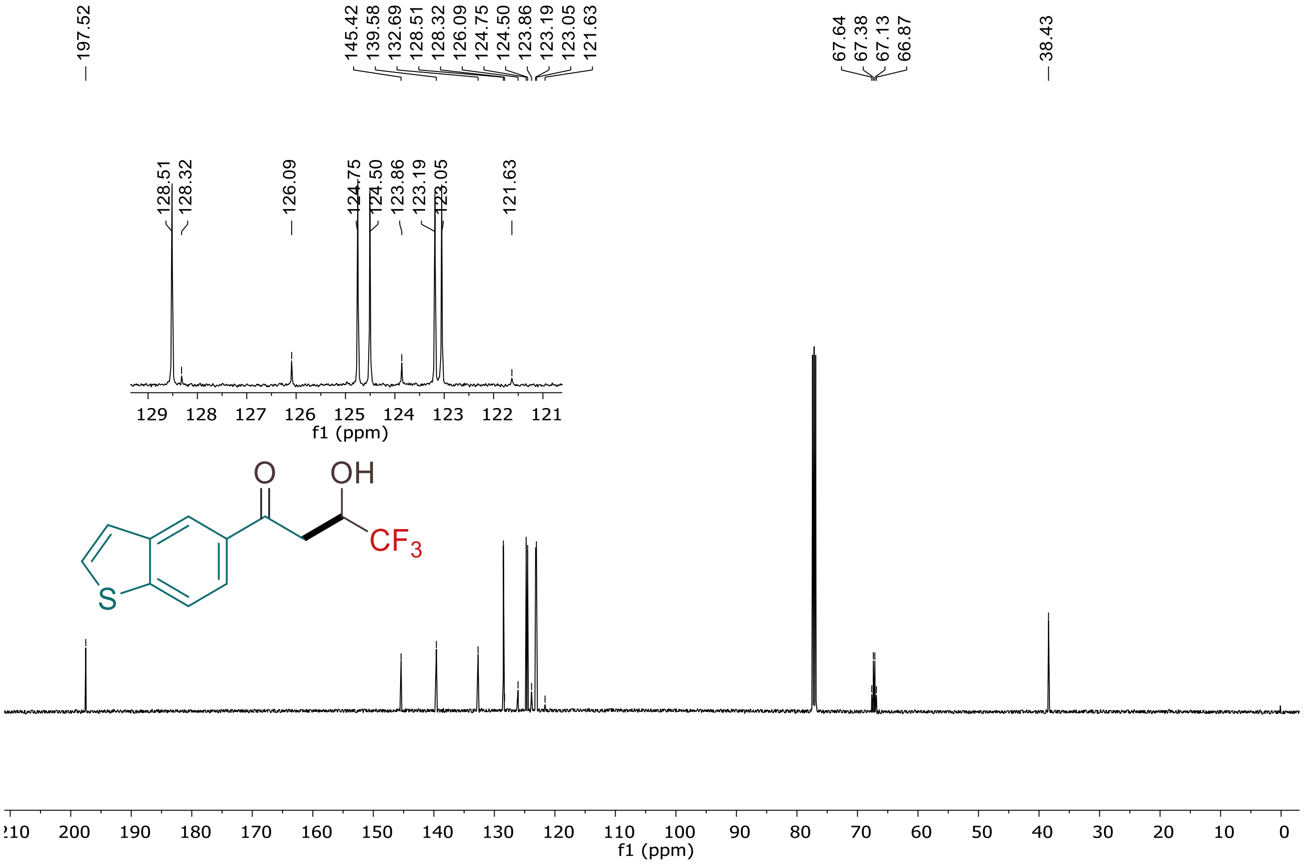

$^{13}C\{^{1}H\}$ NMR (126 MHz, $CDCl_3$) of compound **17**.

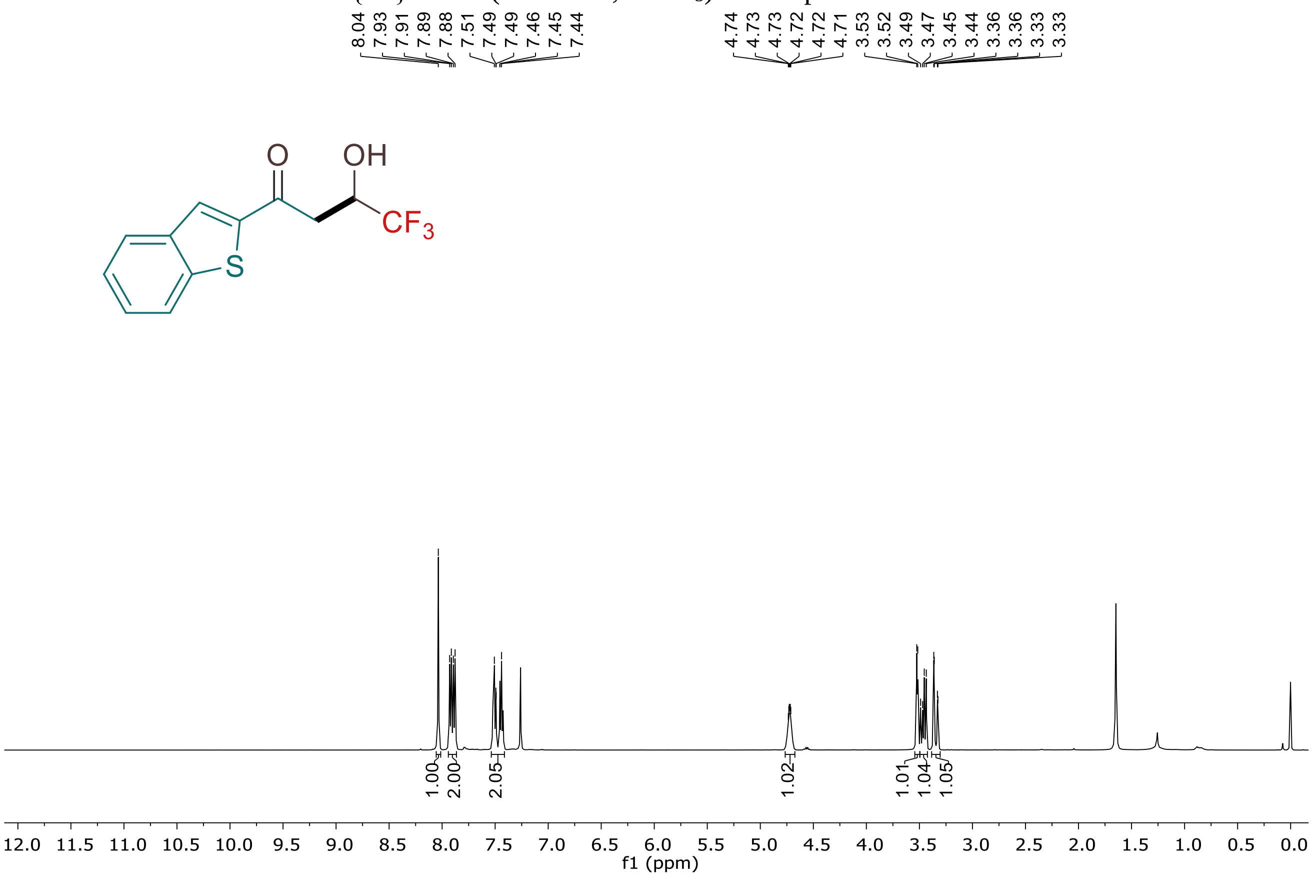

^{1}H NMR (500 MHz, $CDCl_3$) of compound **18**.

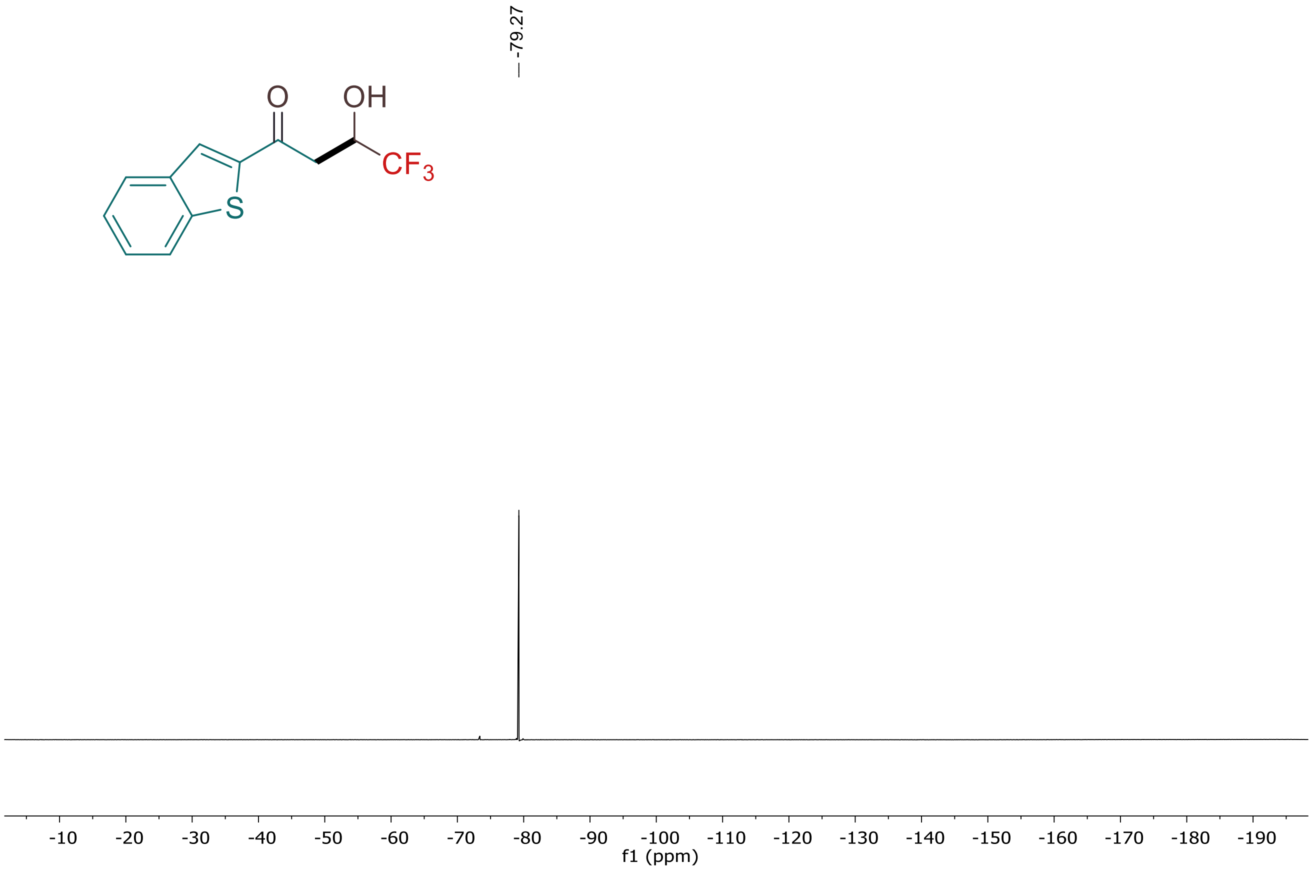

^{19}F{^{1}H} NMR (282 MHz, CDCl$_3$) of compound **18**.

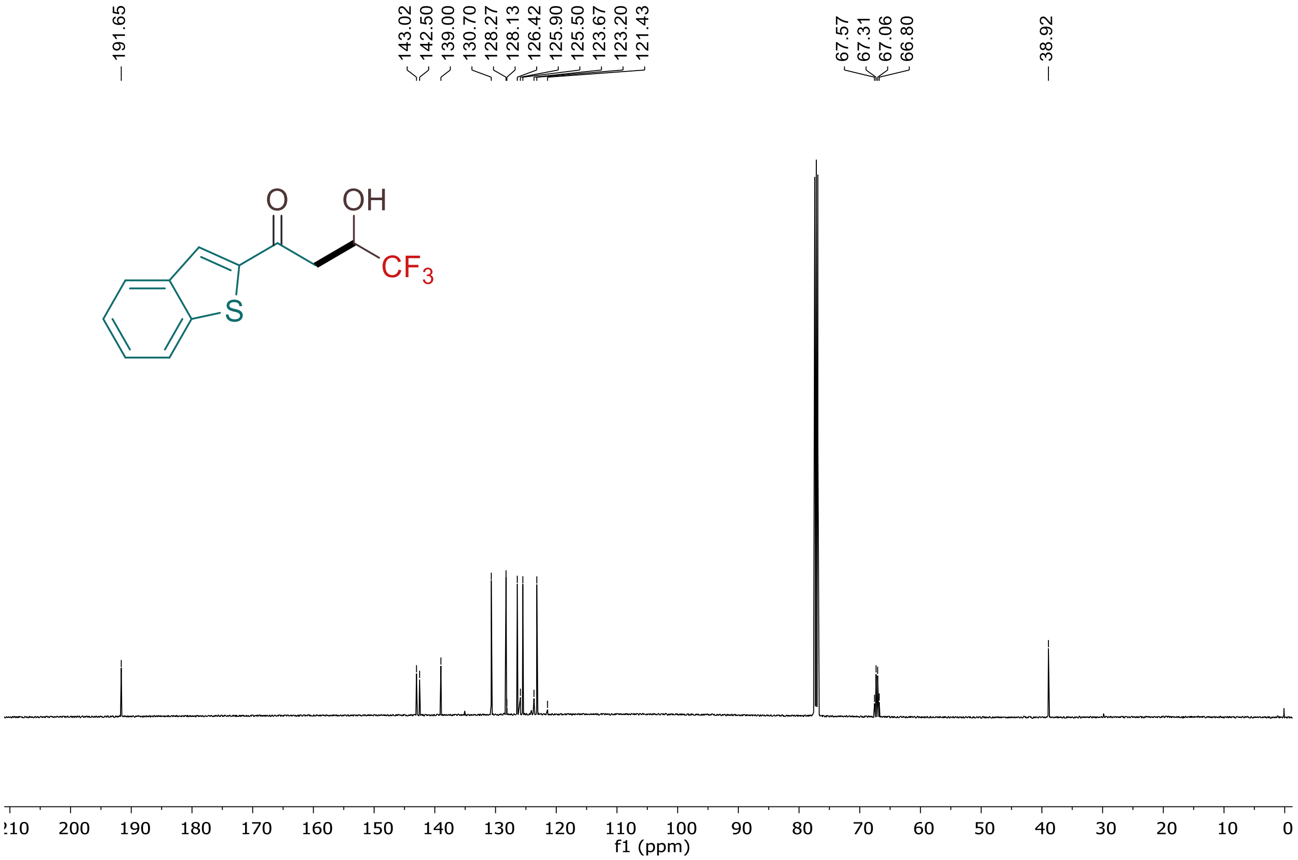

^{13}C{^{1}H} NMR (126 MHz, CDCl$_3$) of compound **18**.

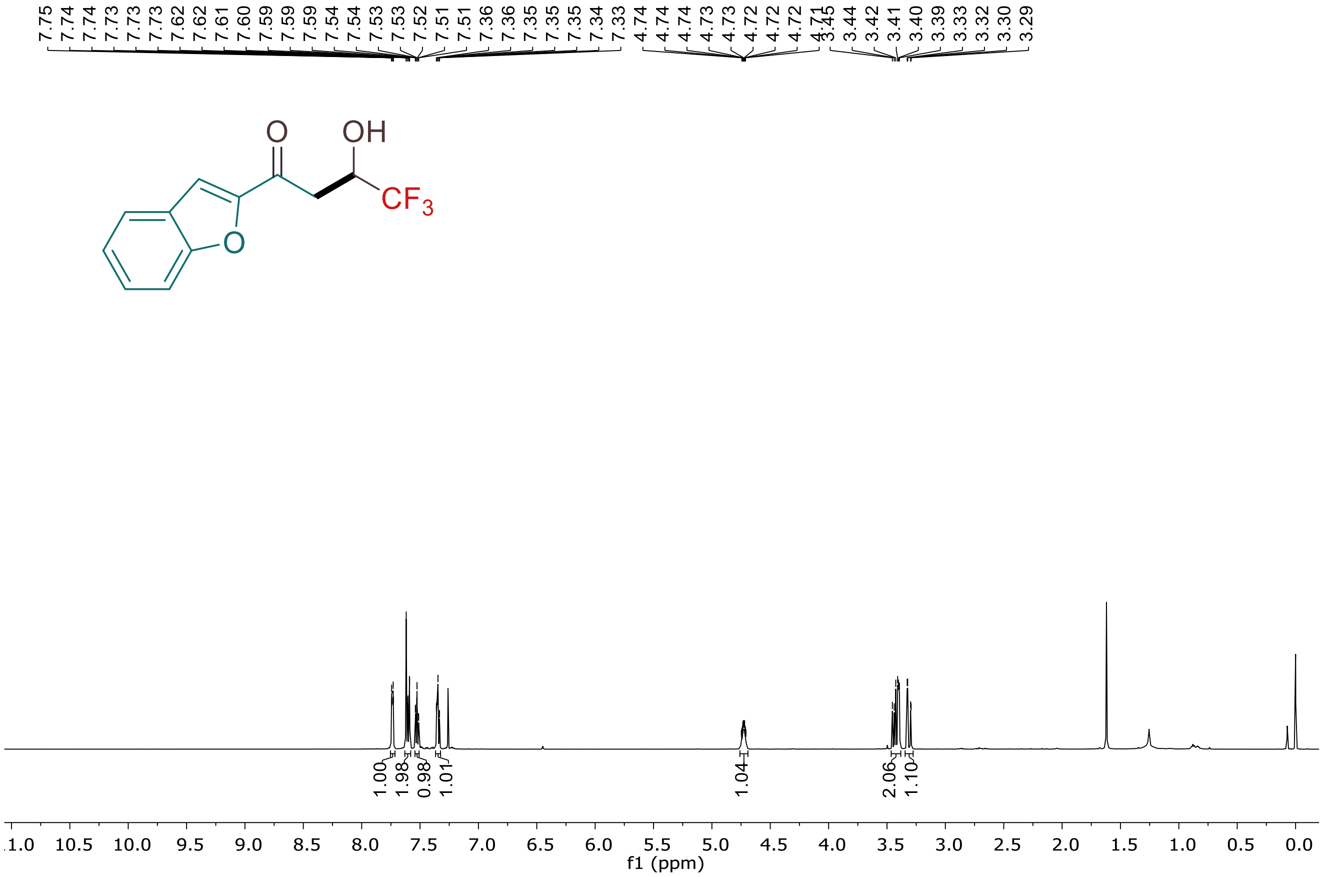

^{1}H NMR (600 MHz, $CDCl_3$) of compound **19**.

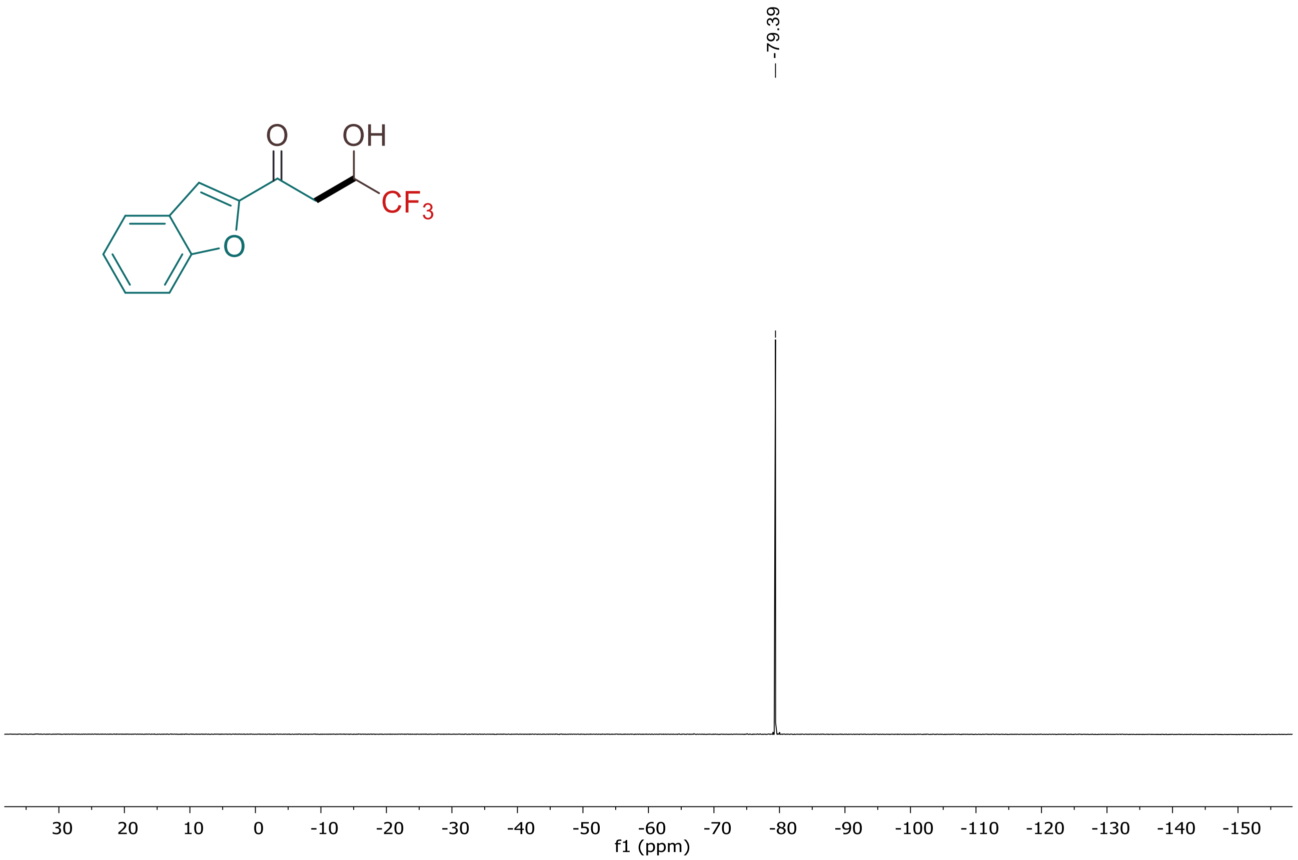

^{19}F{^{1}H} NMR (282 MHz, $CDCl_3$) of compound **19**.

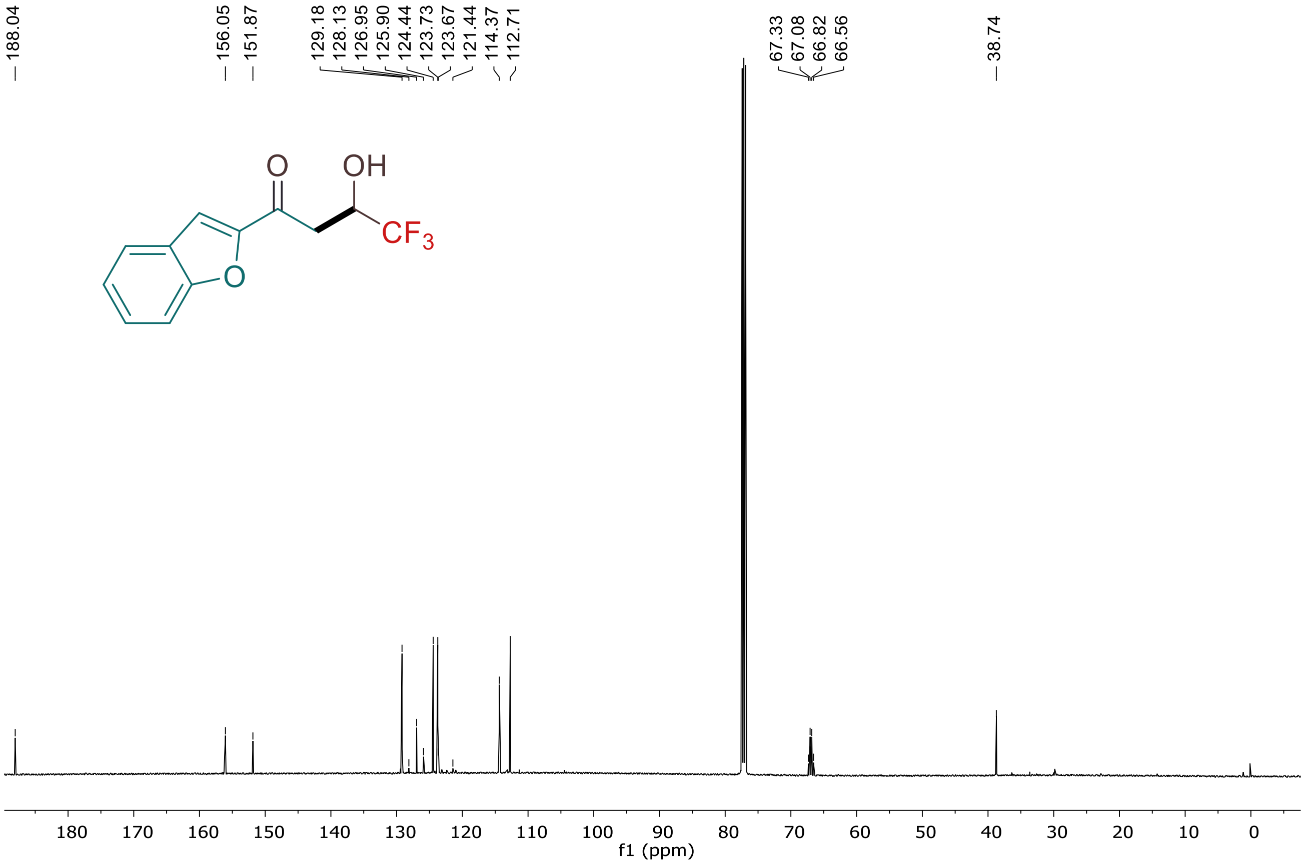

$^{13}C\{^{1}H\}$ NMR (126 MHz, $CDCl_3$) of compound **19**.

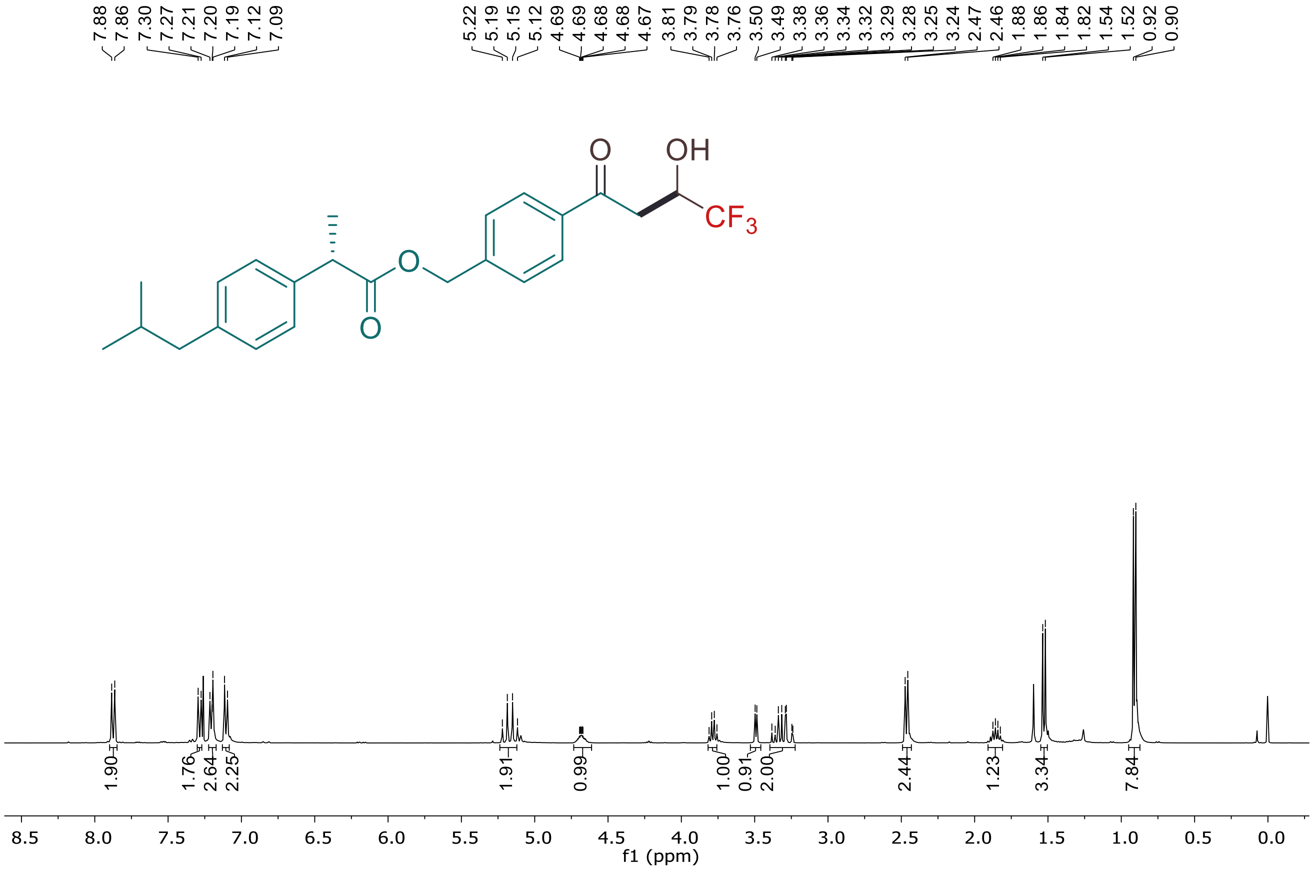

^{1}H NMR (400 MHz, $CDCl_3$) of compound **20**.

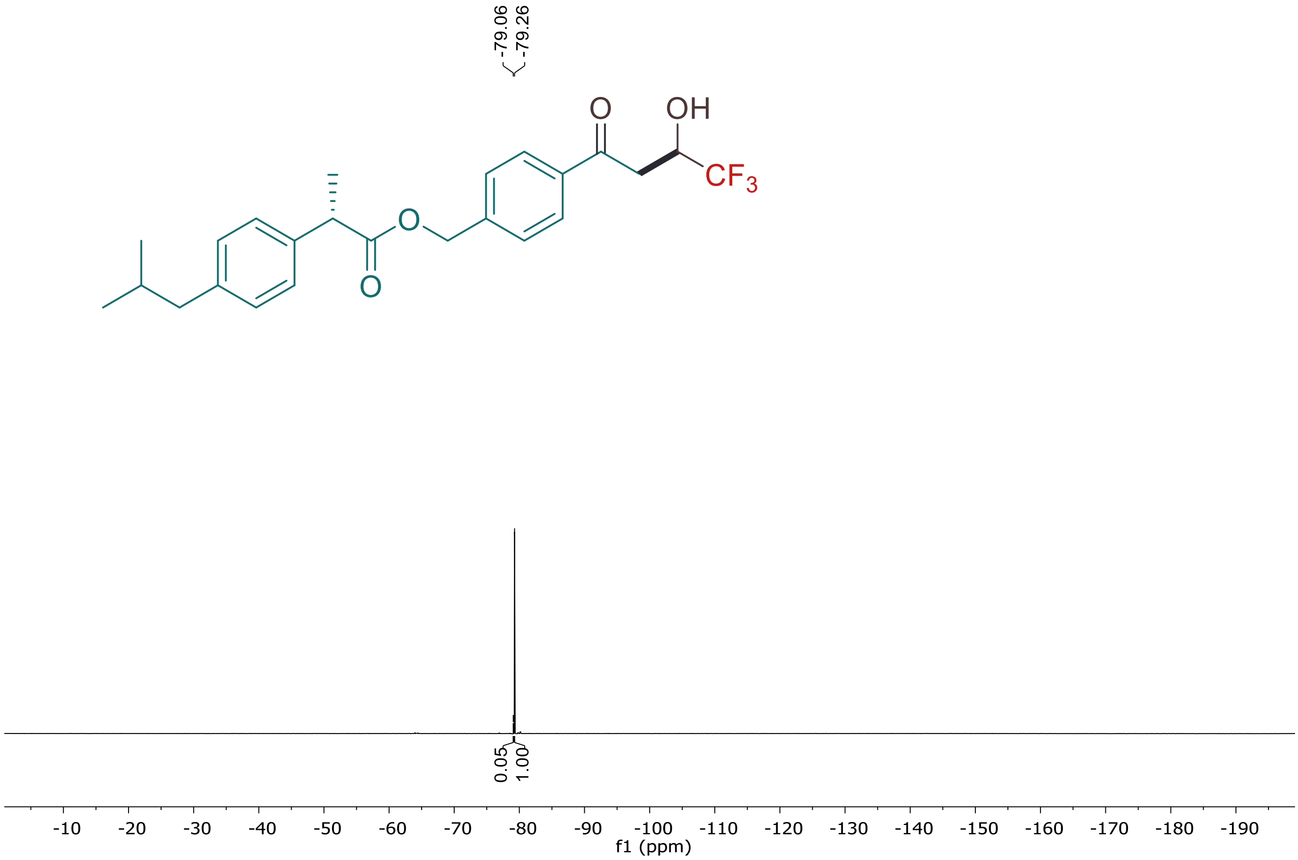

^{19}F{^{1}H} NMR (377 MHz, $CDCl_3$) of compound **20**.

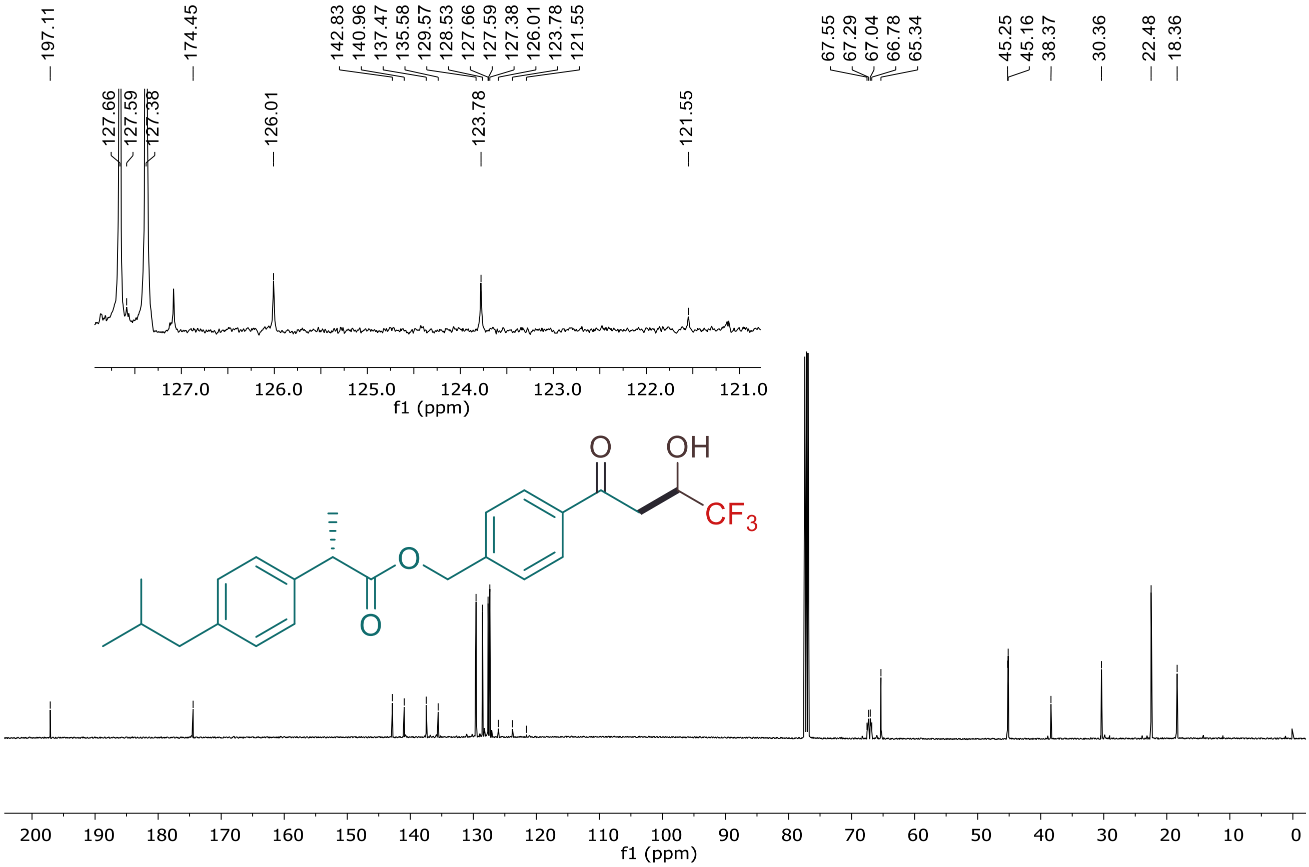

^{13}C{^{1}H} NMR (126 MHz, $CDCl_3$) of compound **20**.

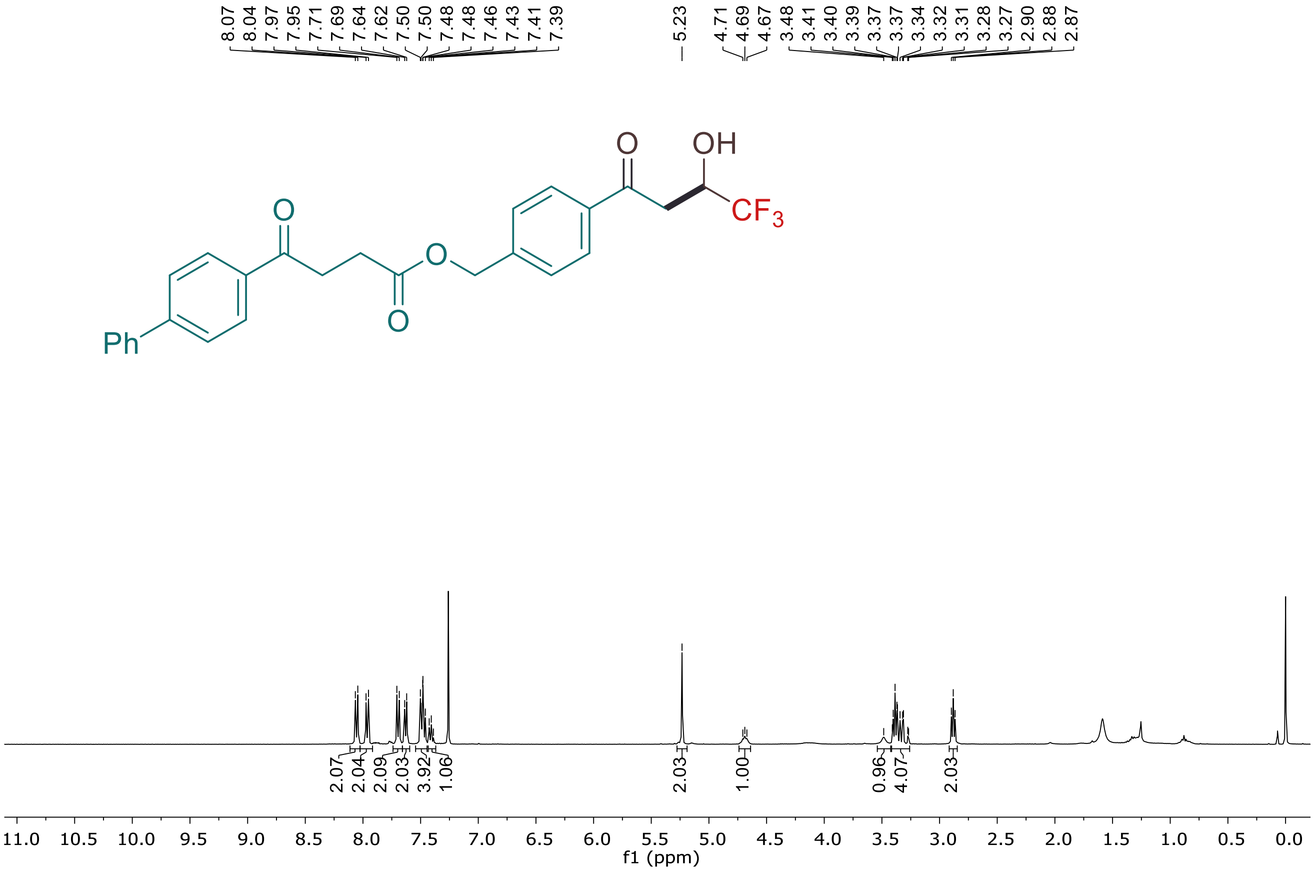

^{1}H NMR (400 MHz, $CDCl_3$) of compound **21**.

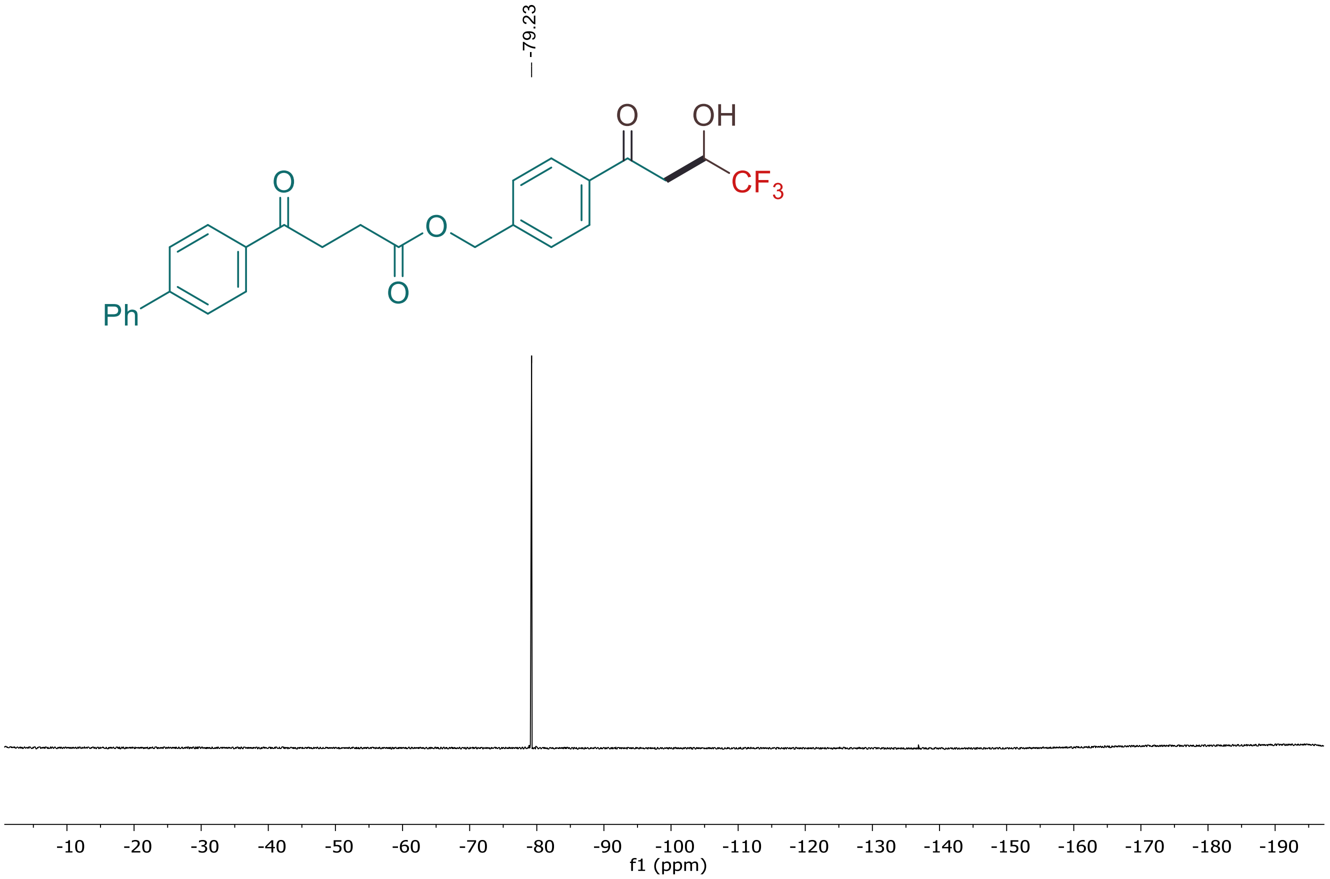

$^{19}F\{^{1}H\}$ NMR (377 MHz, $CDCl_3$) of compound **21**.

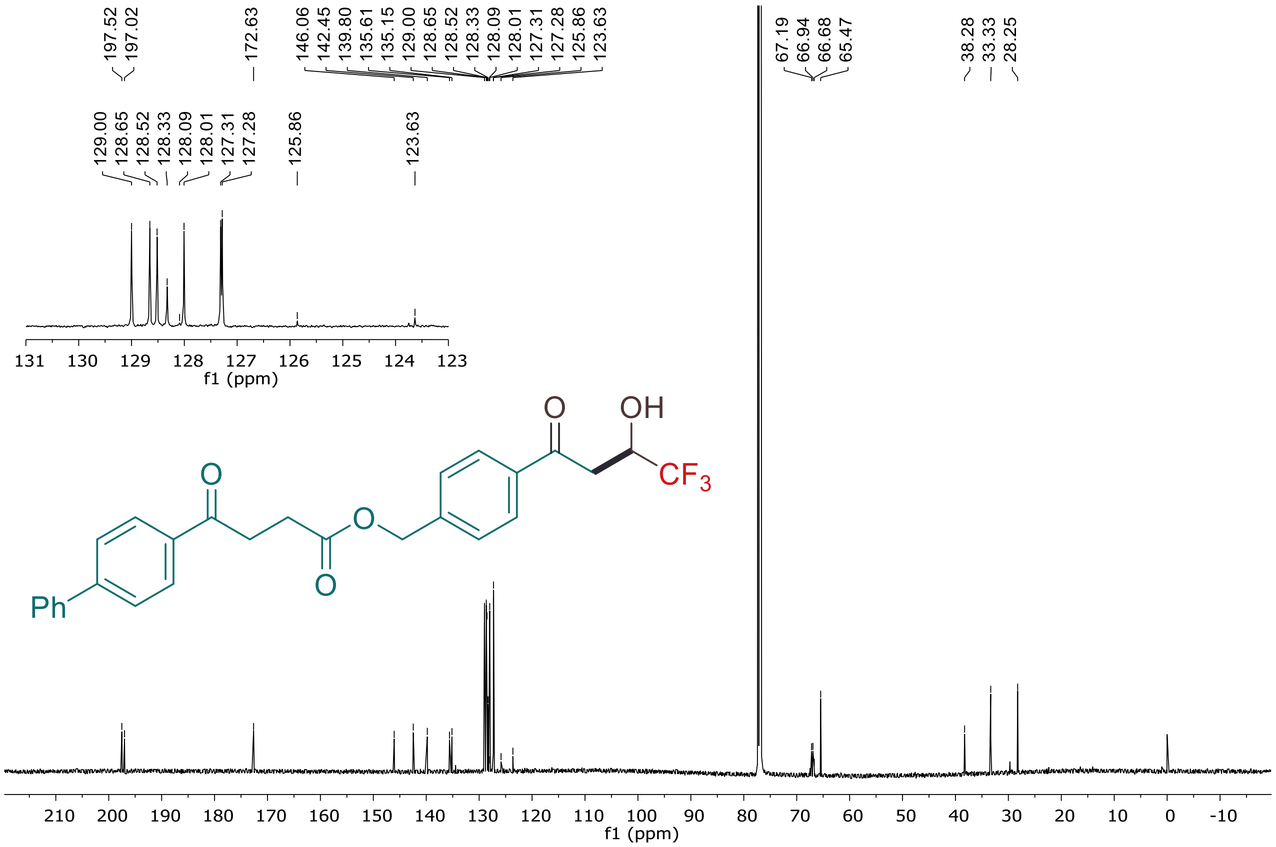

$^{13}C\{^1H\}$ NMR (126 MHz, $CDCl_3$) of compound **21**.

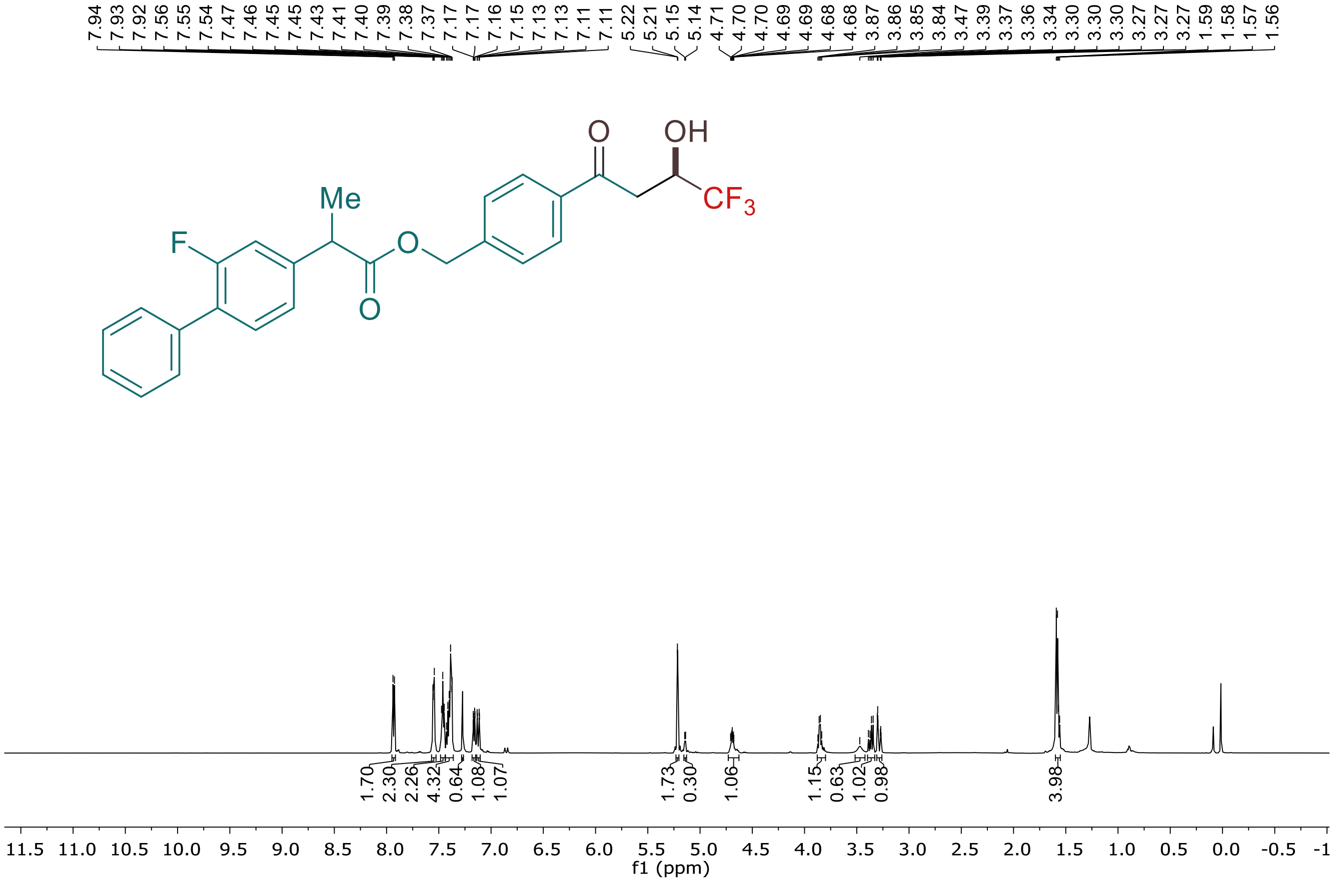

^1H NMR (600 MHz, $CDCl_3$) of compound **22**.

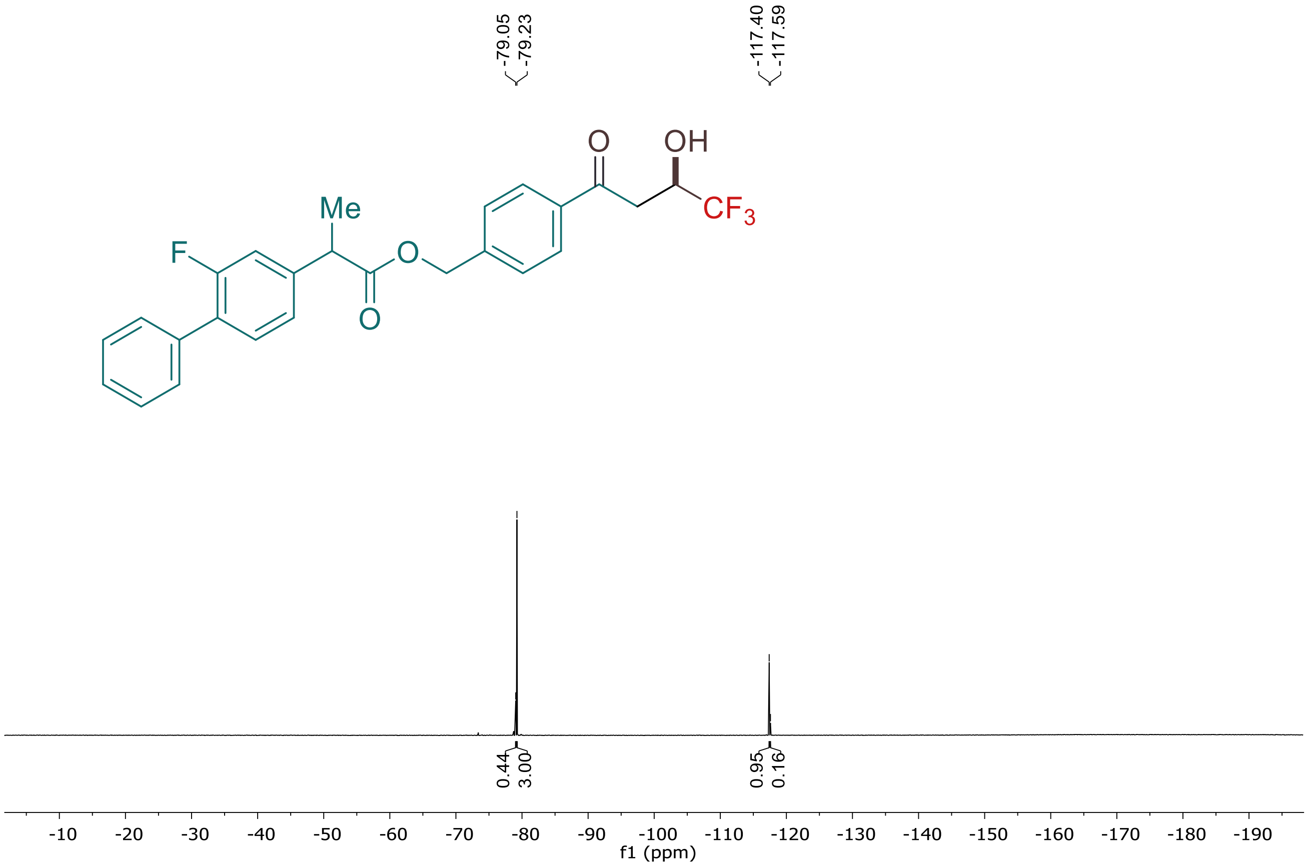

^{19}F{^{1}H} NMR (282 MHz, $CDCl_3$) of compound **22**.

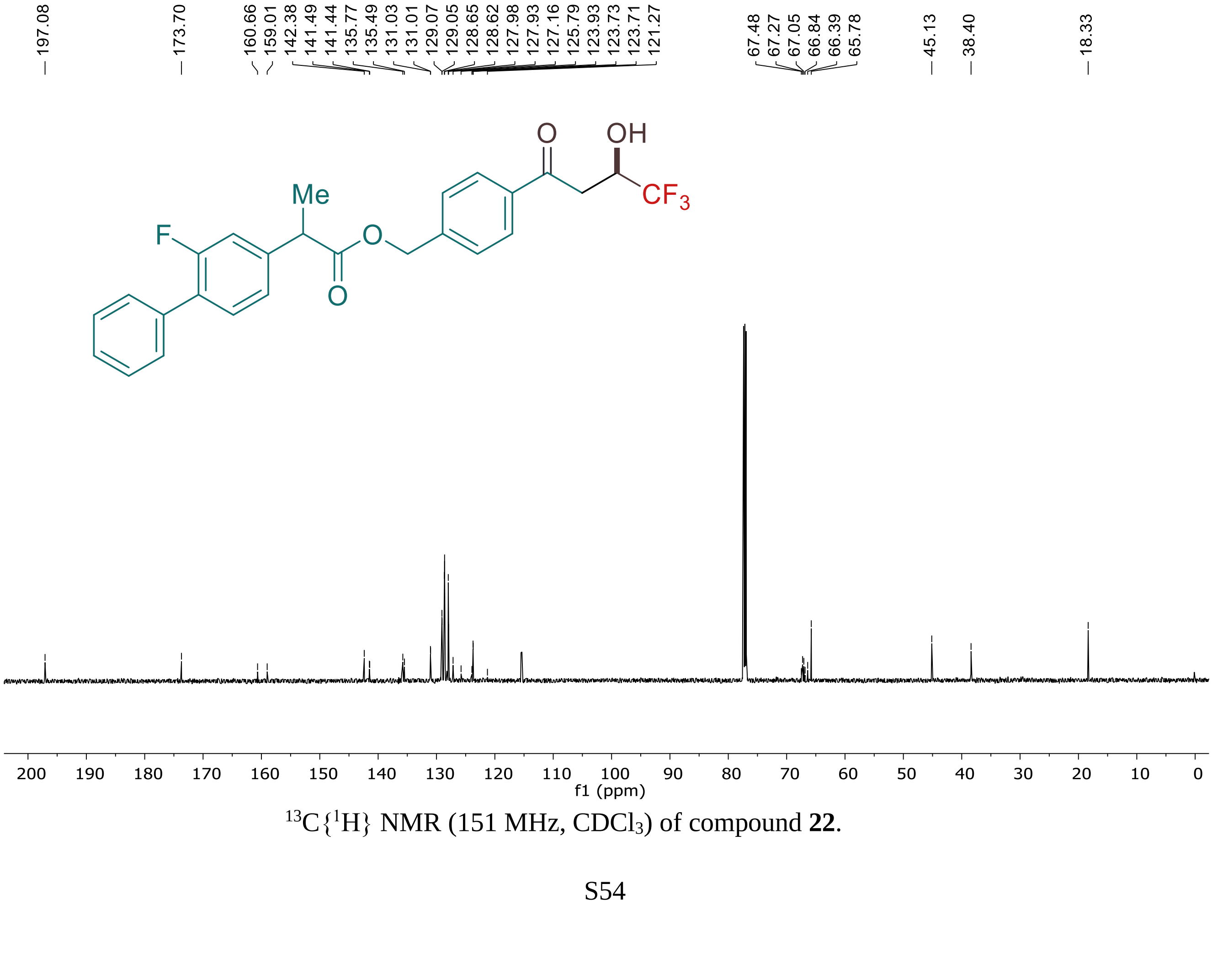

^{13}C{^{1}H} NMR (151 MHz, $CDCl_3$) of compound **22**.

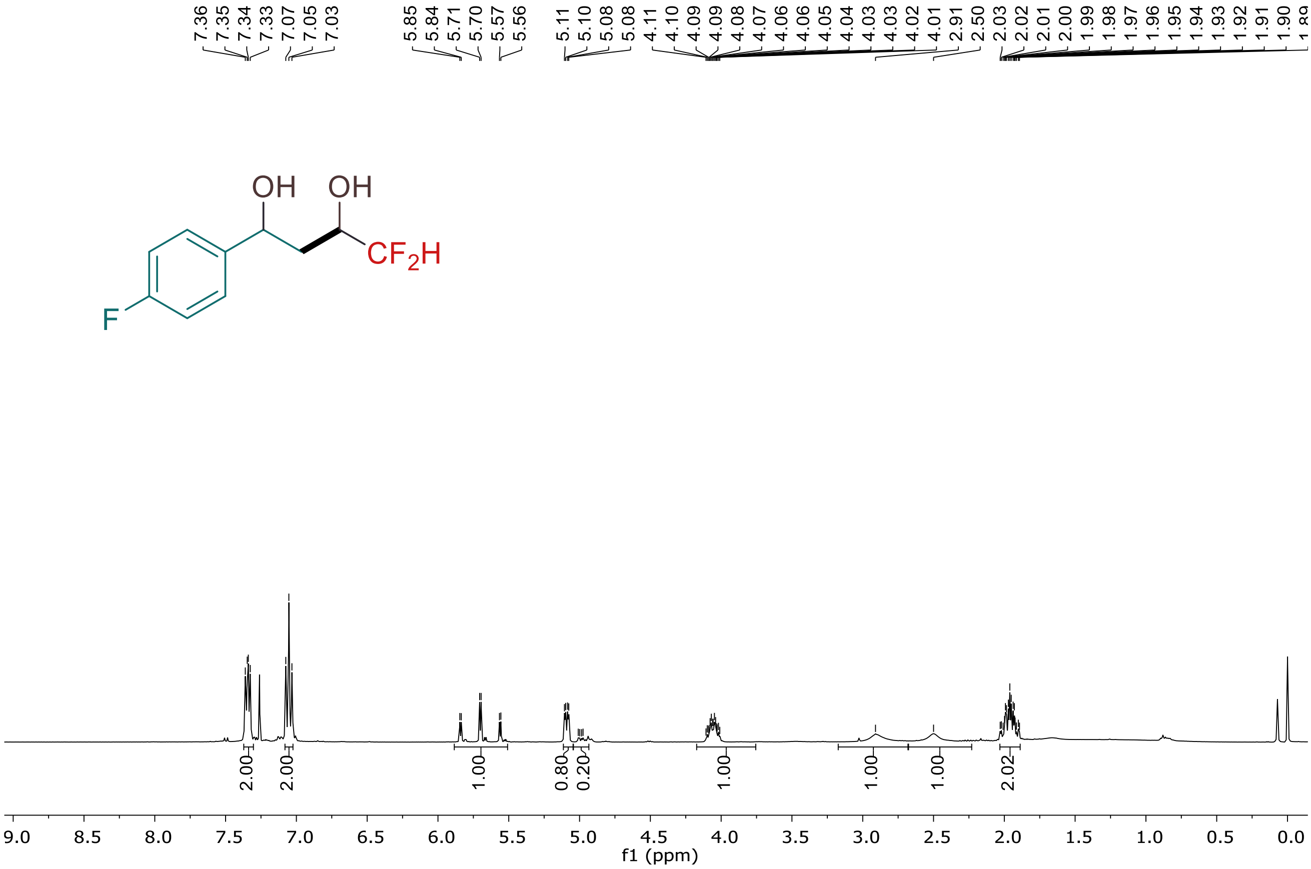

^{1}H NMR (400 MHz, $CDCl_3$) of compound **23**.

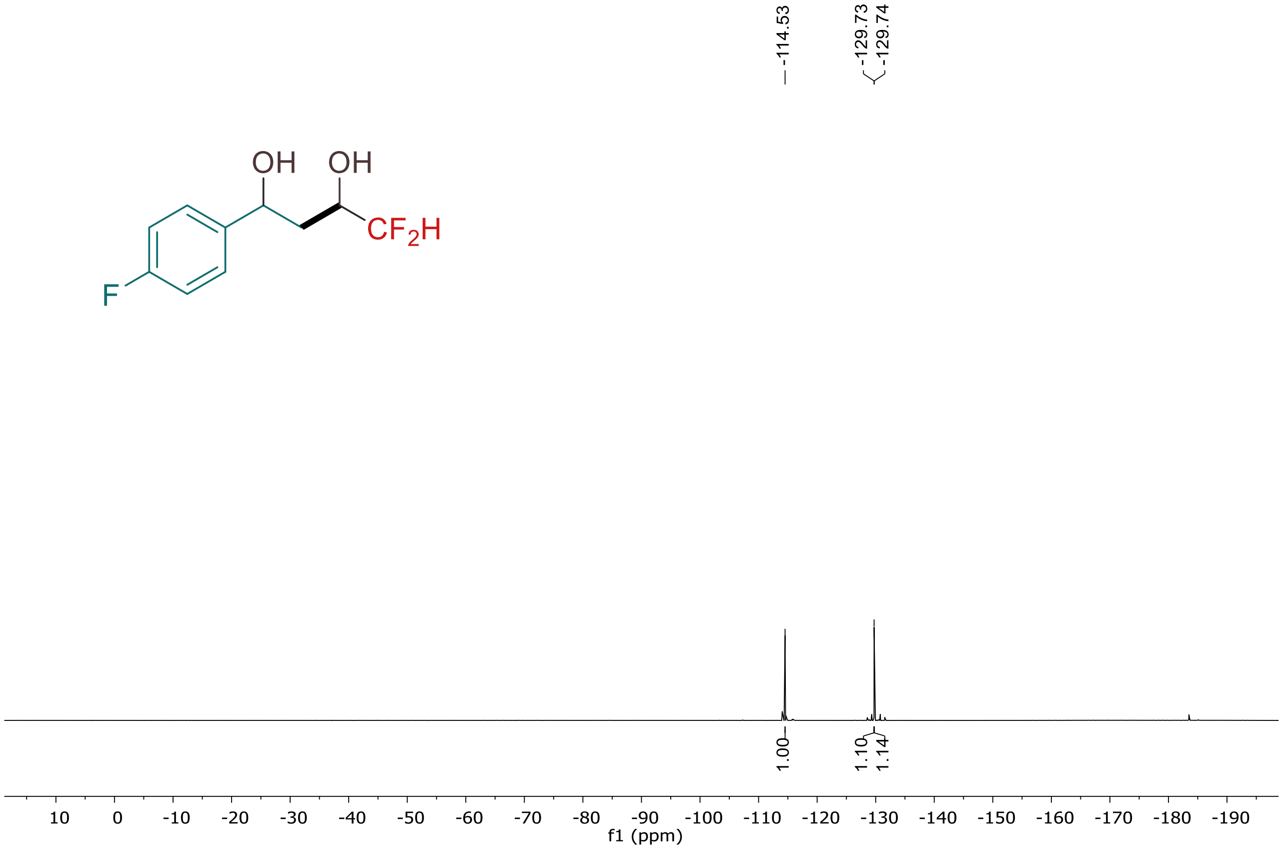

$^{19}F\{^{1}H\}$ NMR (377 MHz, $CDCl_3$) of compound **23**.

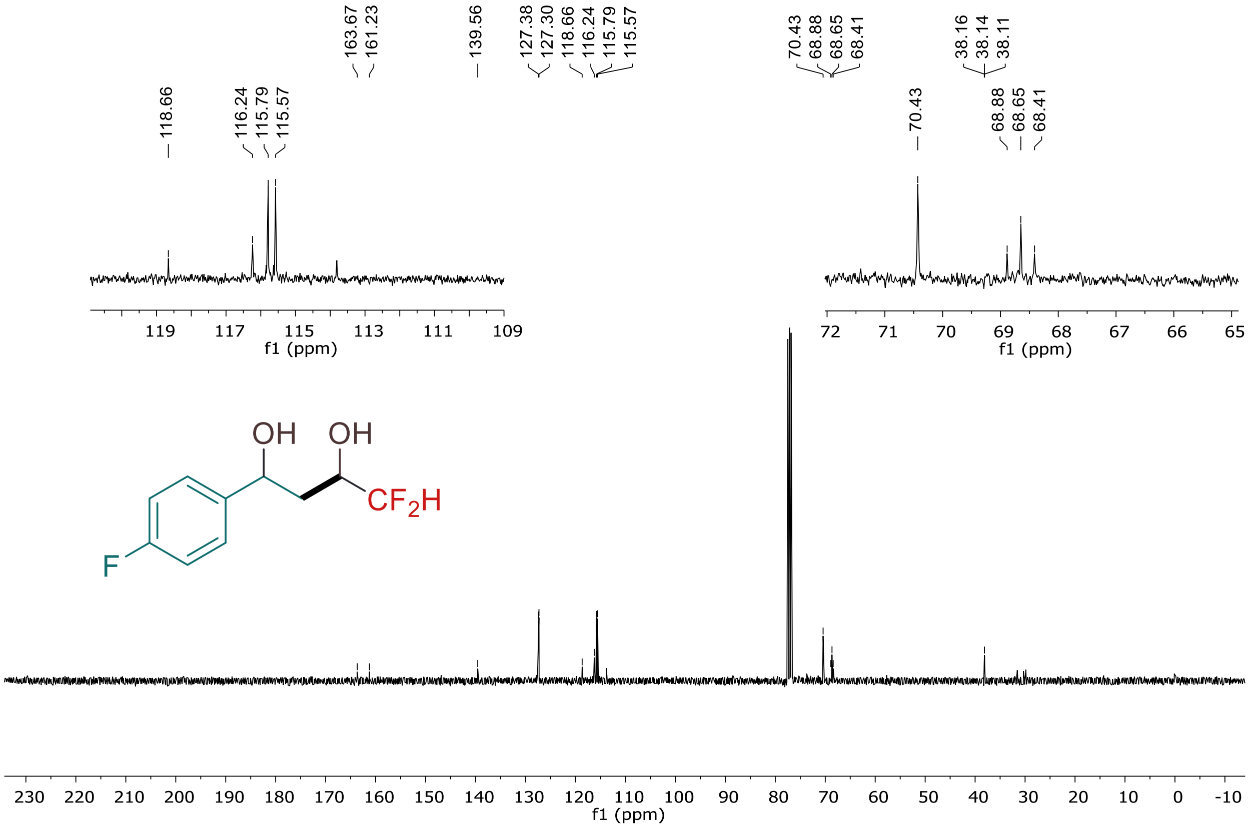

$^{13}C\{^{1}H\}$ NMR (101 MHz, $CDCl_3$) of compound **23**.

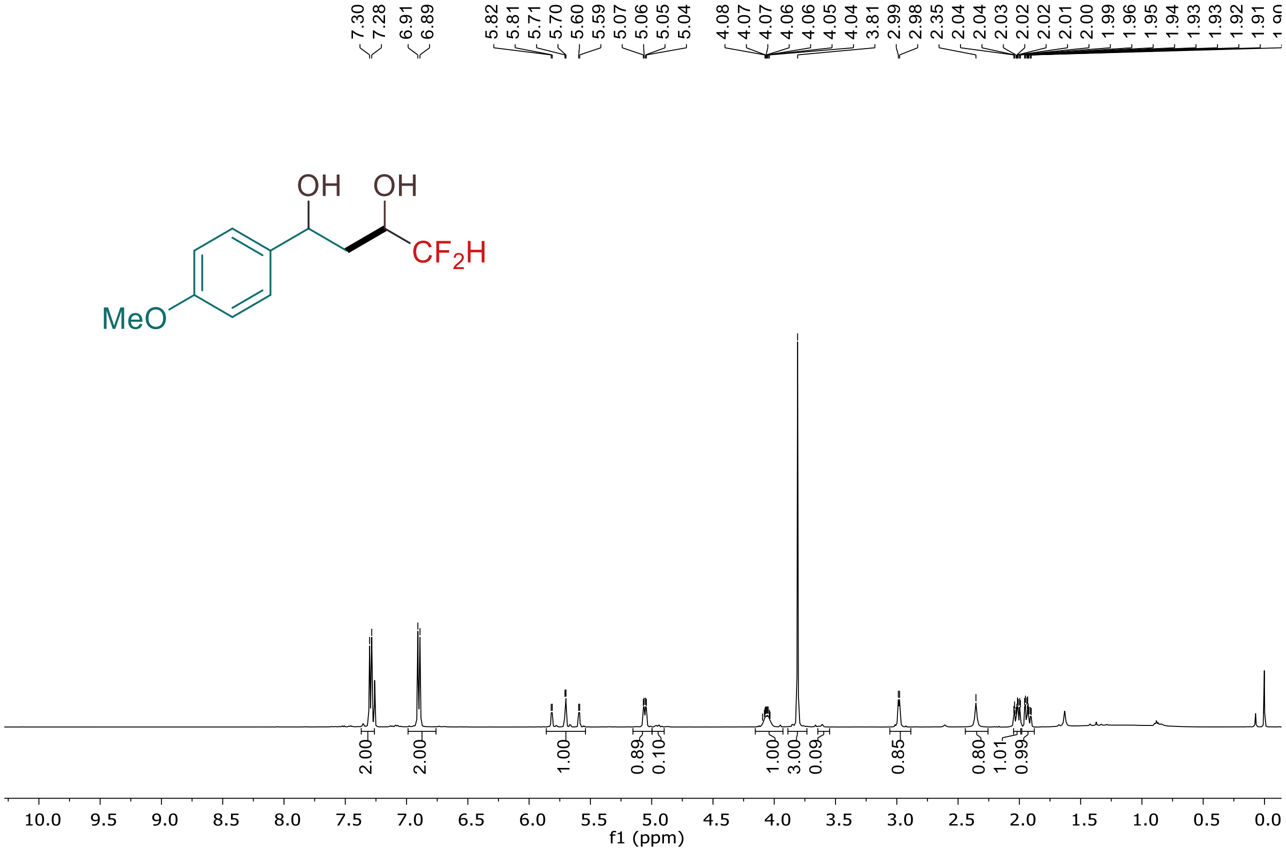

^{1}H NMR (500 MHz, $CDCl_3$) of compound **24**.

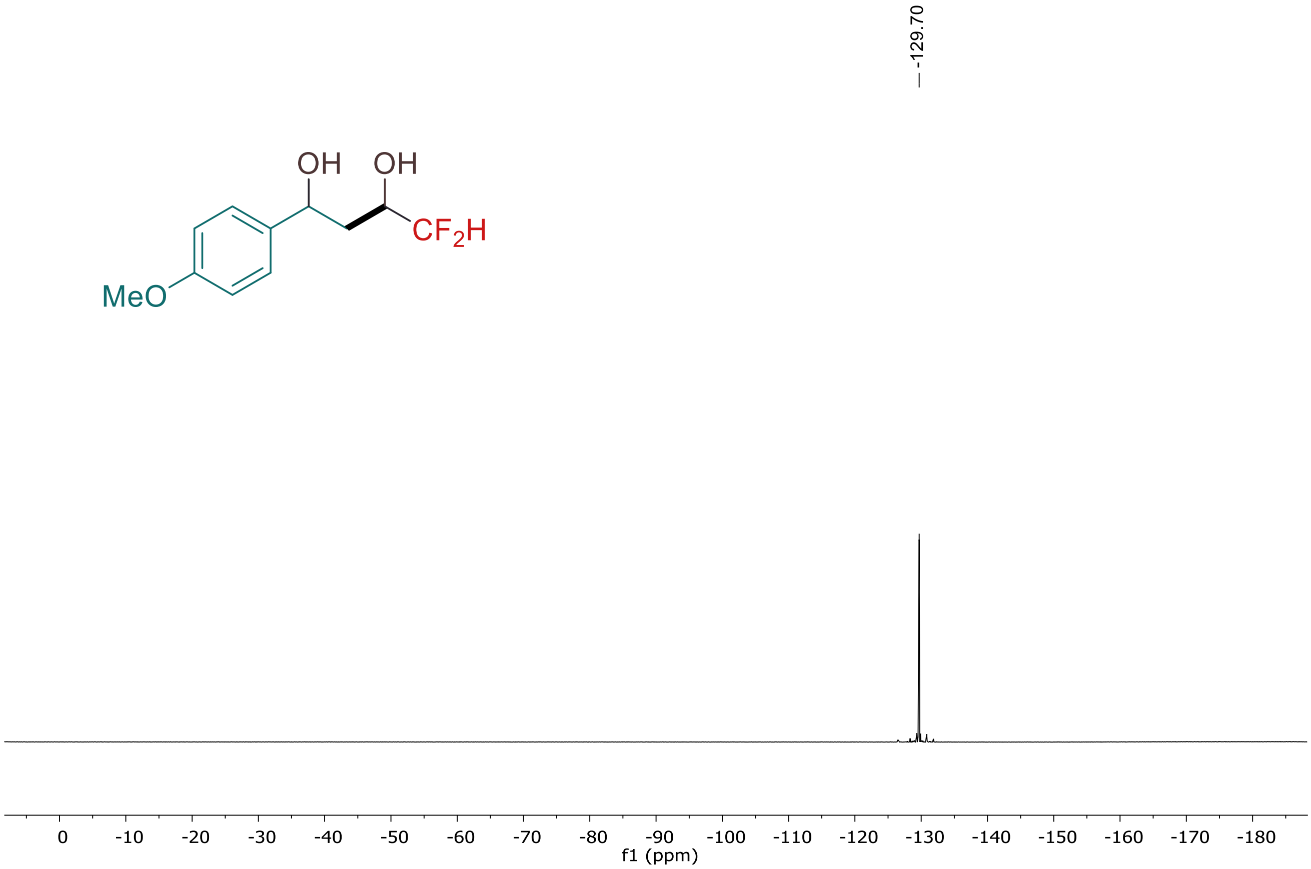

$^{19}F\{^{1}H\}$ NMR (377 MHz, $CDCl_3$) of compound **24**.

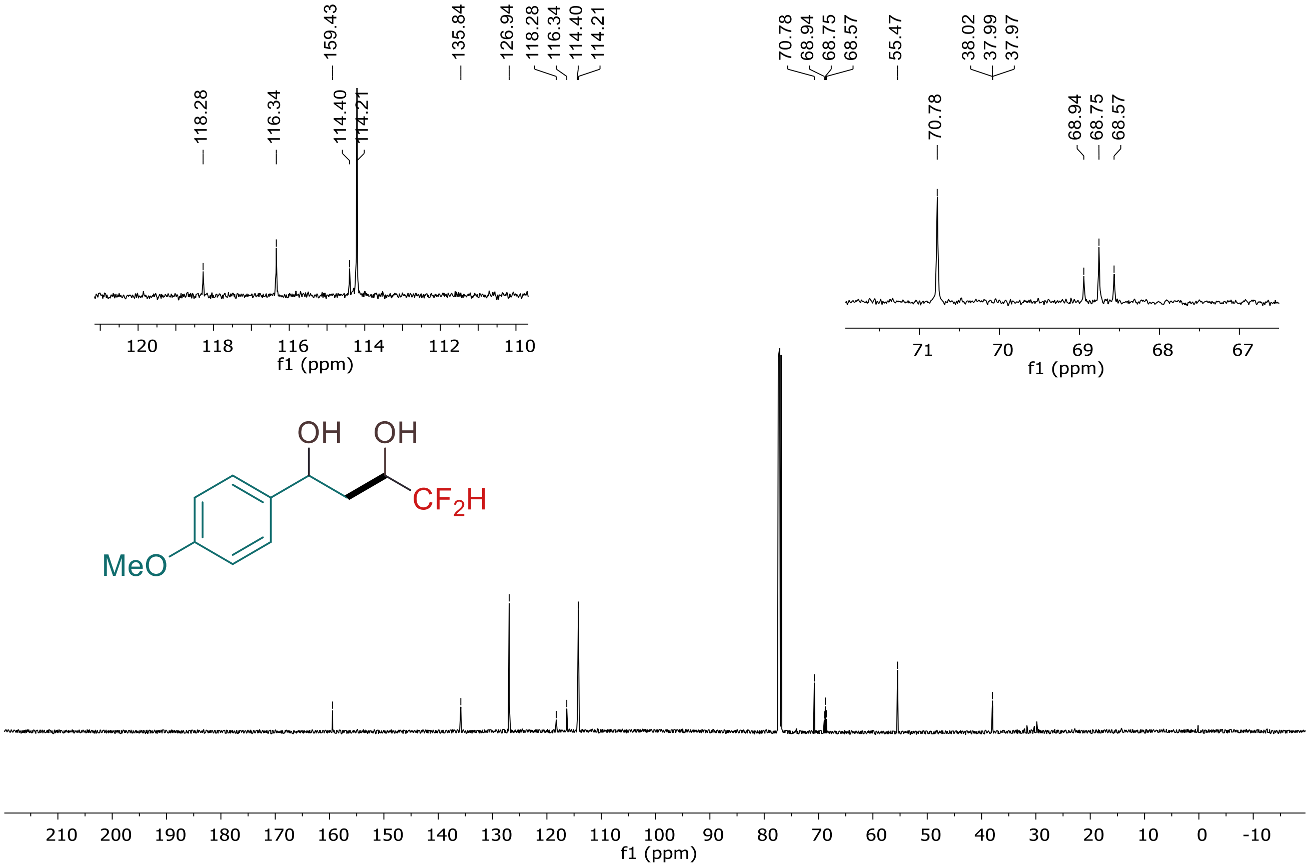

$^{13}C\{^{1}H\}$ NMR (126 MHz, $CDCl_3$) of compound **24**.

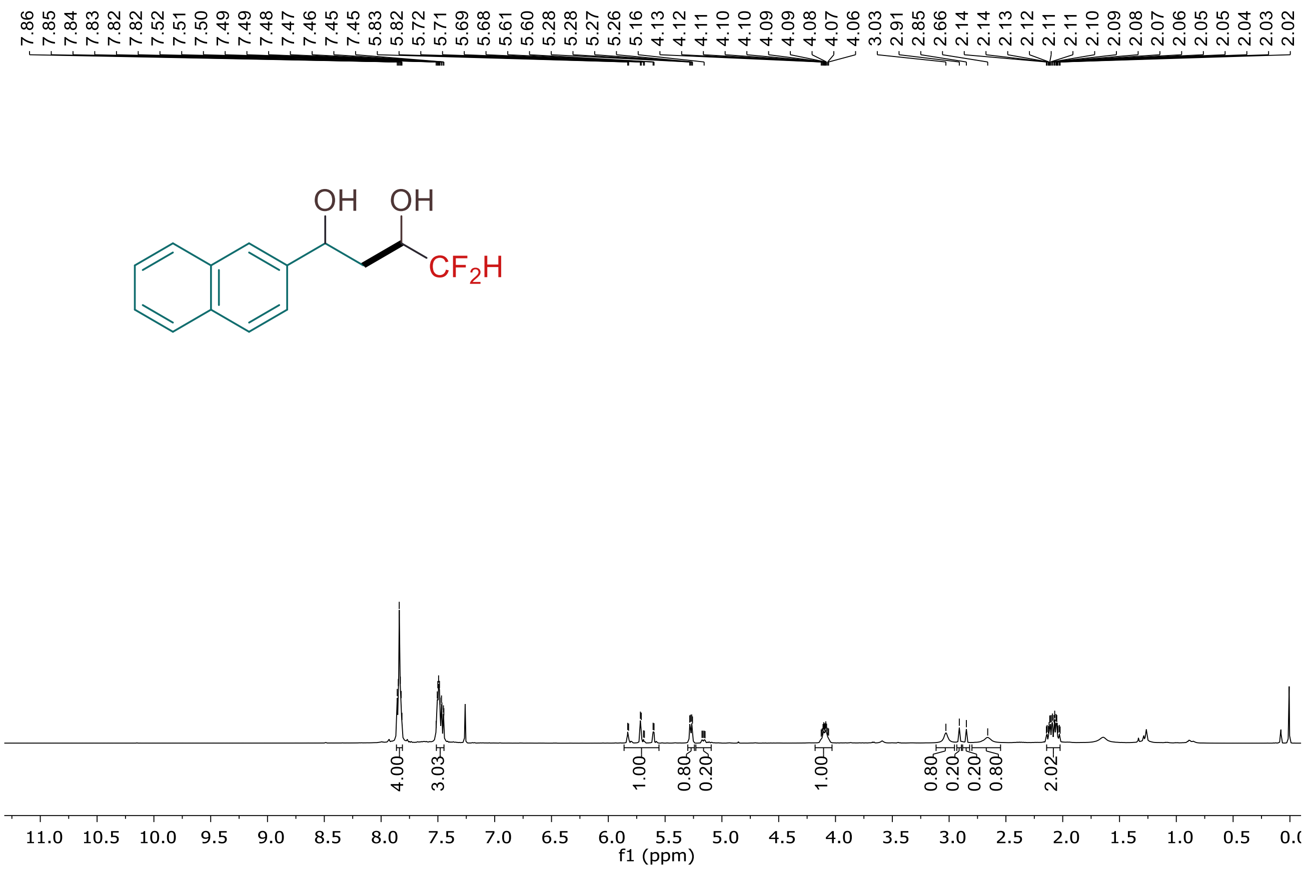

^{1}H NMR (500 MHz, $CDCl_3$) of compound **25**.

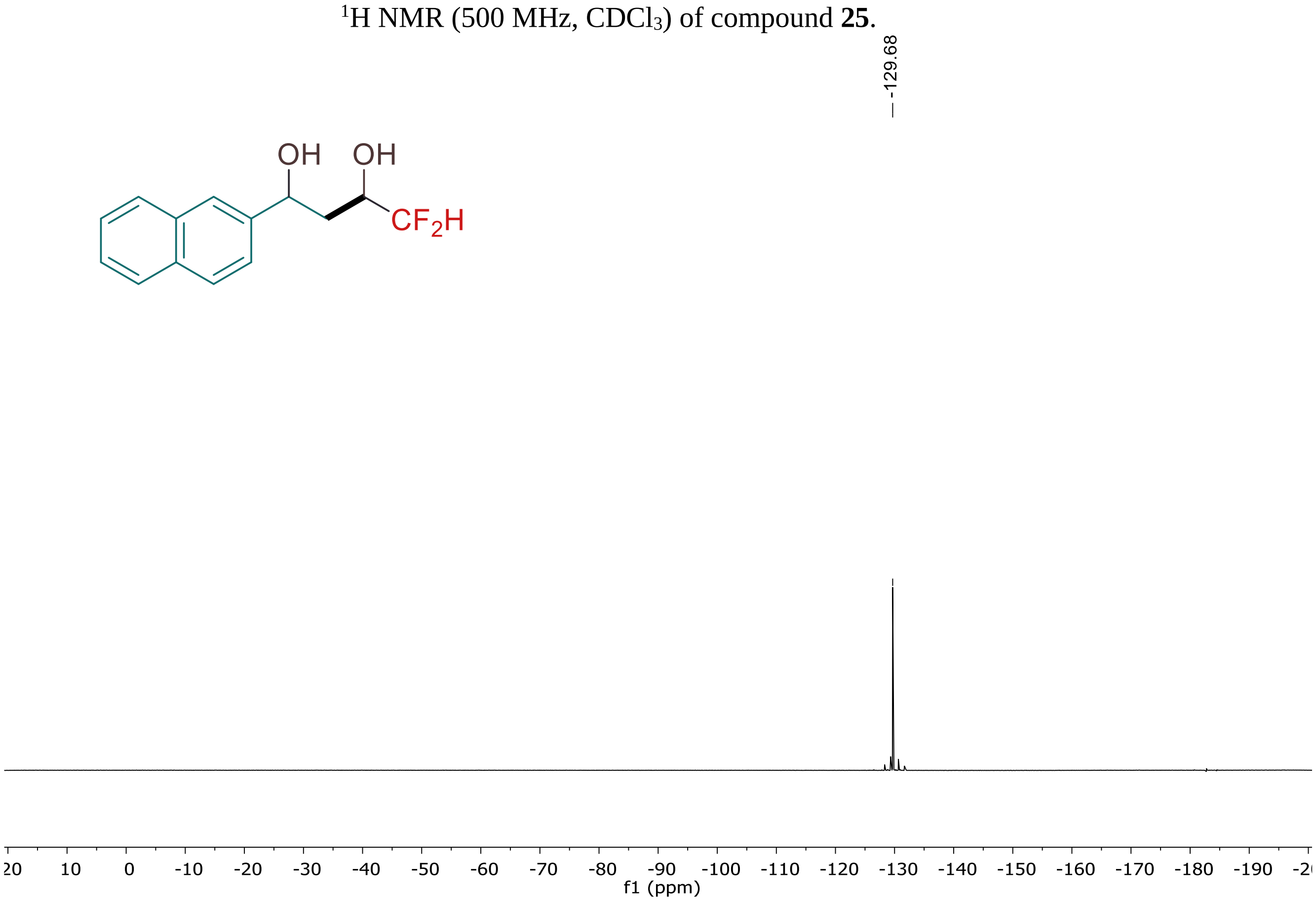

^{19}F{^{1}H} NMR (377 MHz, $CDCl_3$) of compound **25**.

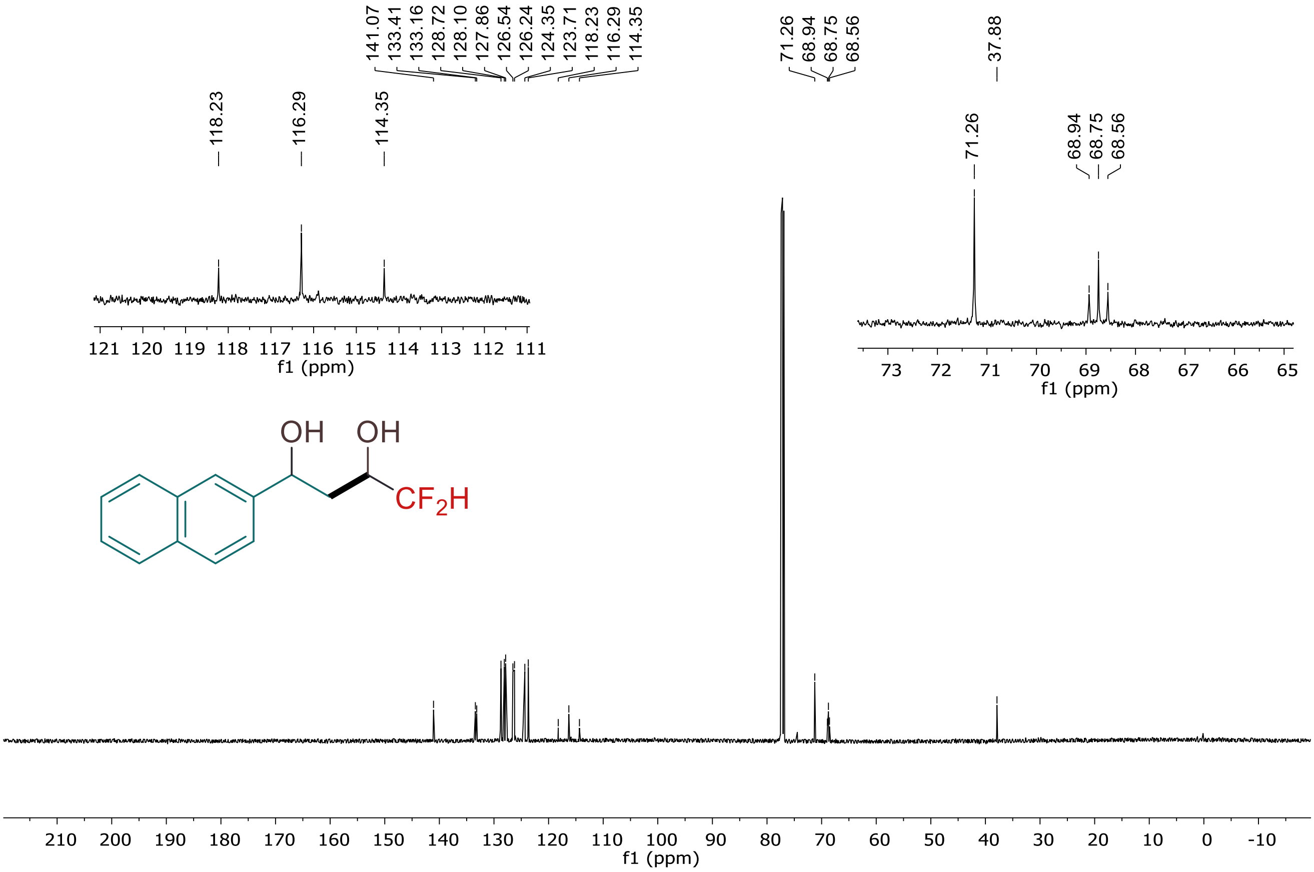

$^{13}C\{^{1}H\}$ NMR (126 MHz, $CDCl_3$) of compound **25**.

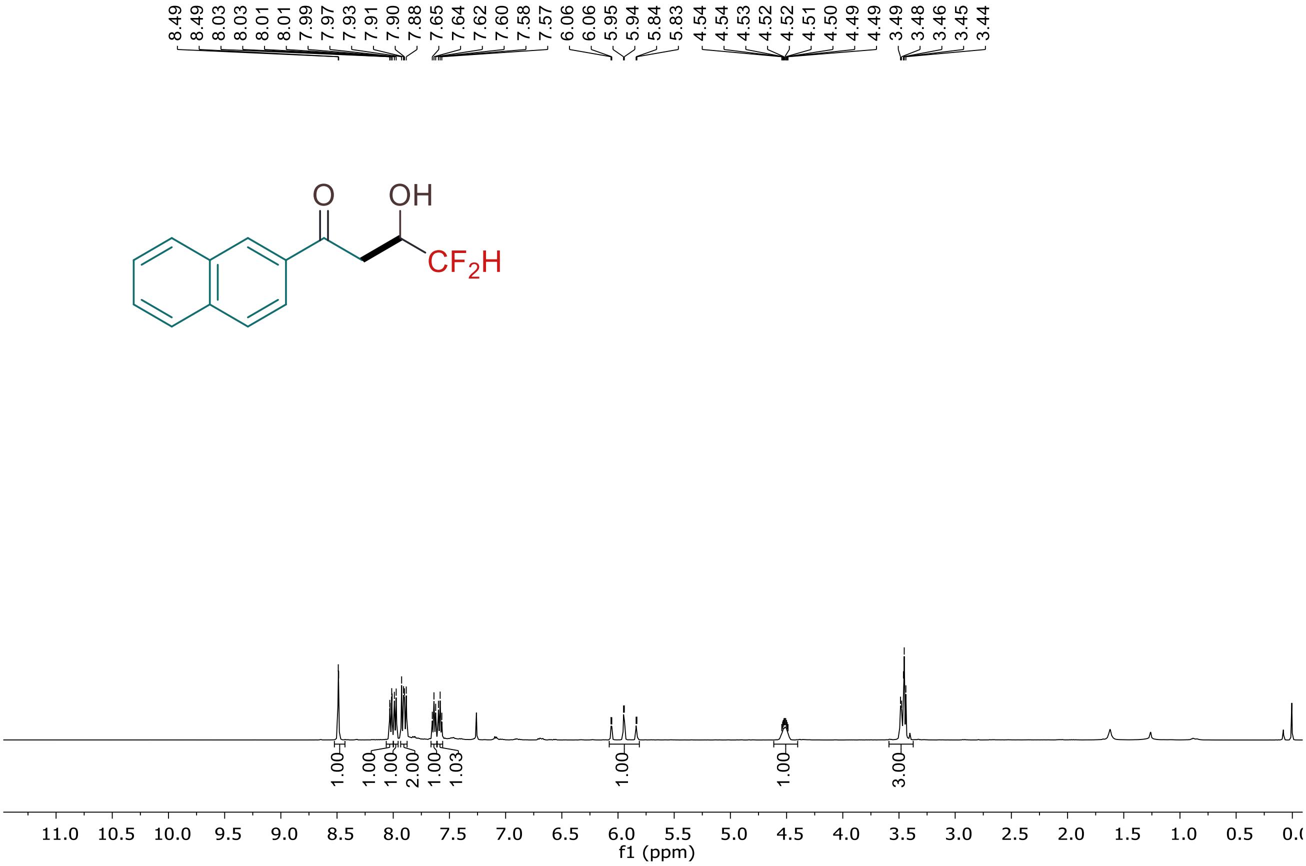

^{1}H NMR (500 MHz, $CDCl_3$) of compound **26**.

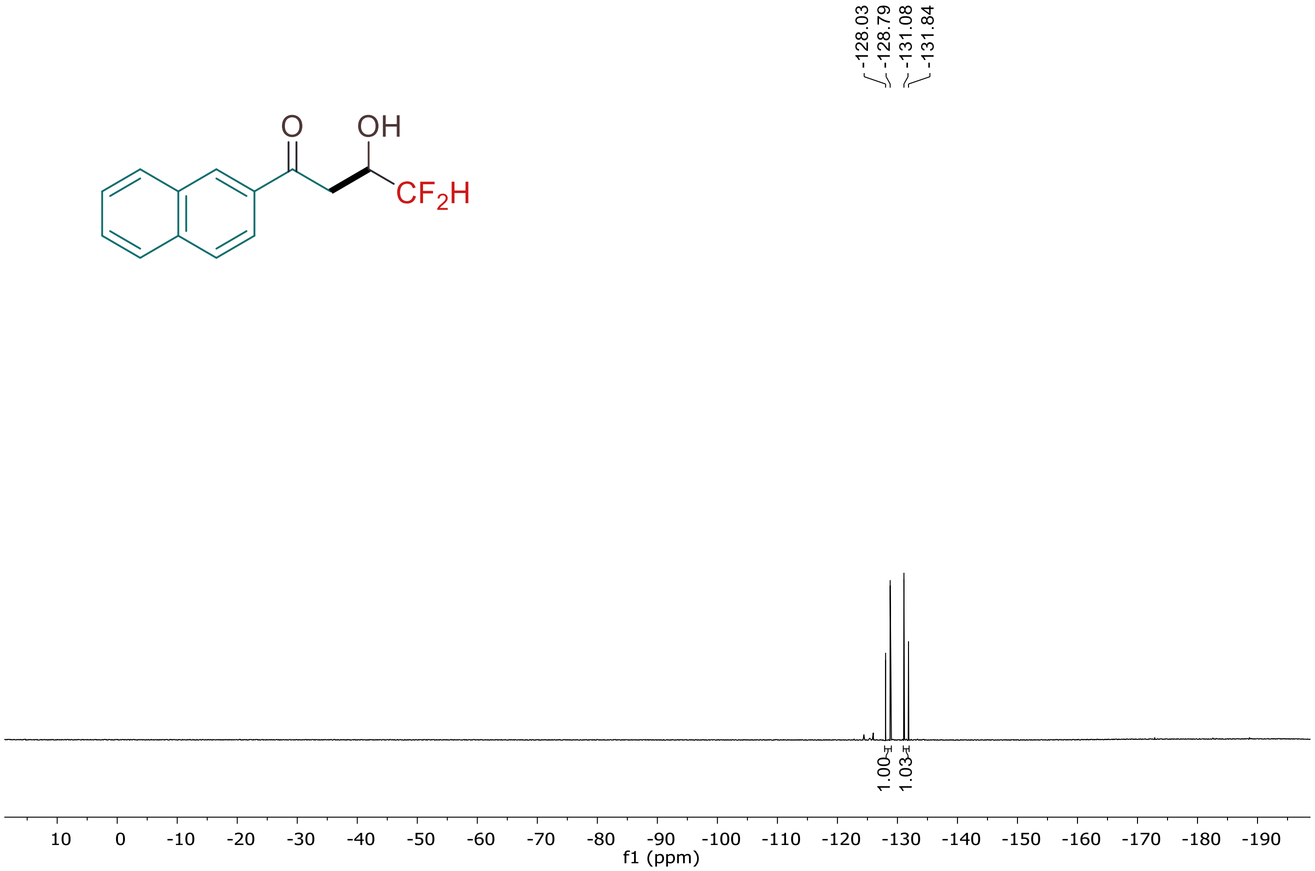

$^{19}F\{^{1}H\}$ NMR (377 MHz, $CDCl_3$) of compound **26**.

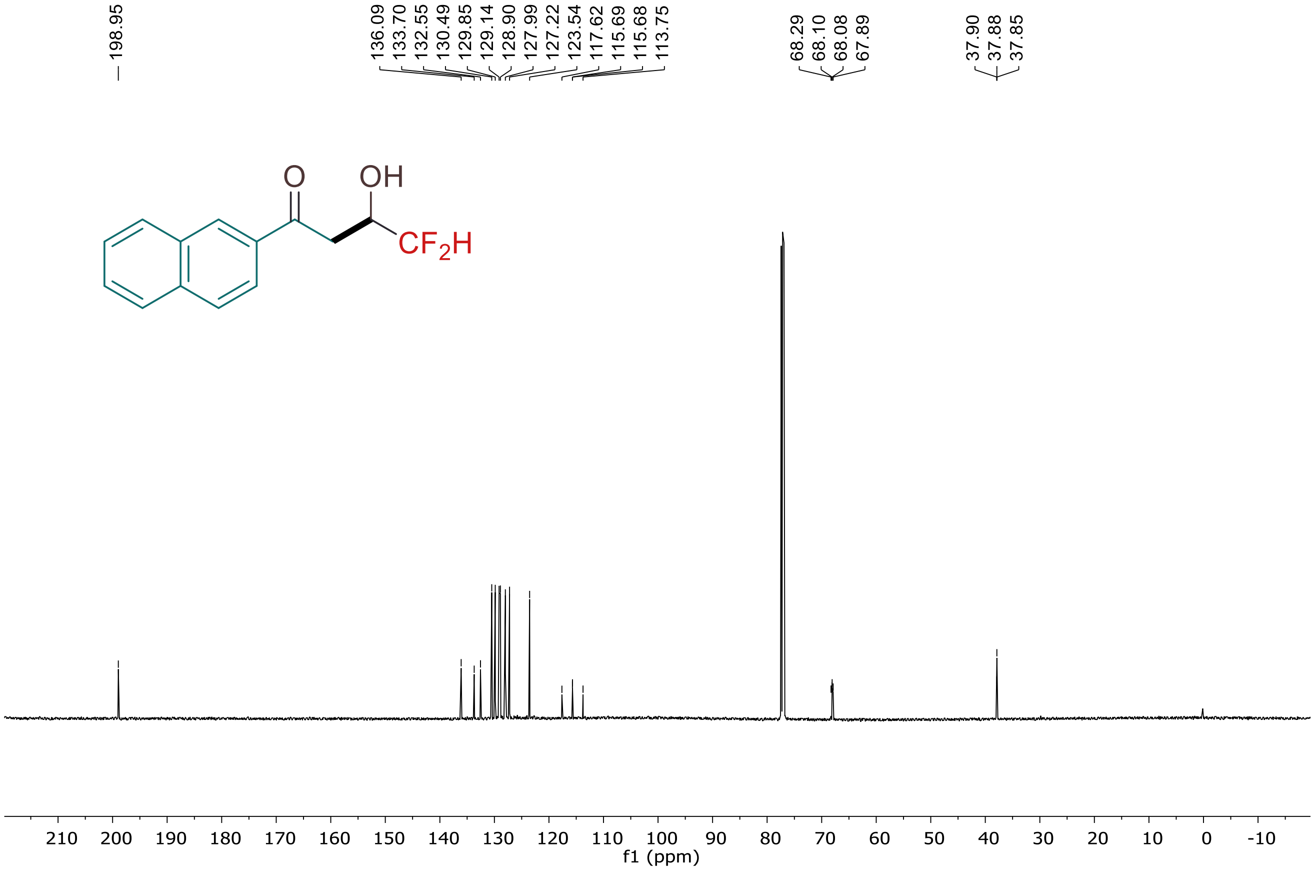

$^{13}C\{^{1}H\}$ NMR (126 MHz, $CDCl_3$) of compound **26**.
